# A multidrug-resistant *Salmonella enterica* Typhimurium DT104 complex lineage circulating among humans and cattle in the USA lost the ability to produce pertussis-like toxin ArtAB

**DOI:** 10.1099/mgen.0.001050

**Published:** 2023-07-04

**Authors:** Laura M. Carroll, Nicolo Piacenza, Rachel A. Cheng, Martin Wiedmann, Claudia Guldimann

**Affiliations:** ^1^​ Department of Clinical Microbiology, SciLifeLab, Umeå University, Umeå, Sweden; ^2^​ Laboratory for Molecular Infection Medicine Sweden (MIMS), Umeå University, Umeå, Sweden; ^3^​ Umeå Centre for Microbial Research, Umeå University, Umeå, Sweden; ^4^​ Integrated Science Lab, Umeå University, Umeå, Sweden; ^5^​ Chair for Food Safety and Analytics, Ludwig-Maximillians-University Munich, Munich, Germany; ^6^​ Department of Food Science and Technology, Virginia Tech, Blacksburg, VA, USA; ^7^​ Department of Food Science, Cornell University, Ithaca, NY, USA

**Keywords:** ArtAB, DT104, Gifsy-1, GogB, prophage, *Salmonella *Typhimurium

## Abstract

*
Salmonella enterica* subsp. *enterica* serotype Typhimurium definitive type 104 (DT104) can infect both humans and animals and is often multidrug-resistant (MDR). Previous studies have indicated that, unlike most *S*. Typhimurium, the overwhelming majority of DT104 strains produce pertussis-like toxin ArtAB via prophage-encoded genes *artAB*. However, DT104 that lack *artAB* have been described on occasion. Here, we identify an MDR DT104 complex lineage circulating among humans and cattle in the USA, which lacks *artAB* (i.e. the ‘U.S. *artAB*-negative major clade’; *n*=42 genomes). Unlike most other bovine- and human-associated DT104 complex strains from the USA (*n*=230 total genomes), which harbour *artAB* on prophage Gifsy-1 (*n*=177), members of the U.S. *artAB*-negative major clade lack Gifsy-1, as well as anti-inflammatory effector *gogB*. The U.S. *artAB*-negative major clade encompasses human- and cattle-associated strains isolated from ≥11 USA states over a 20-year period. The clade was predicted to have lost *artAB*, Gifsy-1 and *gogB* circa 1985–1987 (95 % highest posterior density interval 1979.0–1992.1). When compared to DT104 genomes from other regions of the world (*n*=752 total genomes), several additional, sporadic *artAB*, Gifsy-1 and/or *gogB* loss events among clades encompassing five or fewer genomes were observed. Using phenotypic assays that simulate conditions encountered during human and/or bovine digestion, members of the U.S. *artAB*-negative major clade did not differ from closely related Gifsy-1/*artAB*/*gogB*-harbouring U.S. DT104 complex strains (ANOVA raw *P*>0.05); thus, future research is needed to elucidate the roles that *artAB*, *gogB* and Gifsy-1 play in DT104 virulence in humans and animals.

## Data Summary

Supplementary Data are available under DOI 10.5281/zenodo.7688792, with URL https://doi.org/10.5281/zenodo.7688792, and also uploaded to Microbiology Society figshare: https://doi.org/10.6084/m9.figshare.22194385.v1[1].

**Impact Statement**

Multi-drug resistant (MDR) *Salmonella enterica* serotype Typhimurium definitive type 104 (DT104) was responsible for a global epidemic among humans and animals throughout the 1990s and continues to circulate worldwide. Previous studies have indicated that the vast majority of DT104 produce pertussis-like toxin ArtAB via prophage-encoded *artAB*. Here, we identify a DT104 complex lineage that has been circulating among cattle and humans across ≥11 USA states for over 20 years, which lacks the ability to produce ArtAB (i.e. the ‘U.S. *artAB*-negative major clade’). The common ancestor of all U.S. *artAB*-negative major clade members lost the ability to produce ArtAB in the 1980s; however, the reason for this loss-of-function event within this well-established pathogen remains unclear. The role that ArtAB plays in DT104 virulence remains elusive, and phenotypic assays conducted here indicate that members of the U.S. *artAB*-negative major clade do not have a significant advantage or disadvantage relative to closely related, Gifsy-1/*artAB*/*gogB*-harbouring U.S. DT104 complex strains when exposed to stressors encountered during human and/or bovine digestion *in vitro*. However, ArtAB heterogeneity within the DT104 complex suggests clade-specific selection for or against maintenance of ArtAB. Thus, future studies querying the virulence characteristics of the U.S. *artAB*-negative major clade are needed.

## Introduction

Prophages, which are viruses located within the genomes of bacteria, play important roles in the evolution of their microbial hosts [2–5]. In addition to possessing machinery that is antagonistic to host cell survival (e.g. virion production, lysis of host cells), many prophages encode accessory genes, which may provide the host with a selective advantage [2, 4, 6], including stress tolerance, resistance to antimicrobials and phages, biofilm formation, increased virulence, and evasion of the host immune system [2, 3, 5–9]. While they may persist within a lineage through vertical transmission [6, 7, 10], prophages can undergo gain and loss events within a population over time [2, 4]. Furthermore, integrated prophages can be hotspots for horizontal gene transfer (HGT) and genomic recombination, allowing their bacterial hosts to gain, lose and exchange genetic information [5]. Thus, prophage-mediated HGT may confer novel functions, which allow the bacterial host to survive and compete in its environment, potentially contributing to the emergence of novel epidemic lineages [5, 11].

*Salmonella enterica* subsp. *enterica* serotype Typhimurium (*S*. Typhimurium) is among the *Salmonella* serotypes most commonly isolated from human and animal salmonellosis cases worldwide [12, 13] and is known to host a range of prophages within its chromosome [11]. Of particular concern is *S*. Typhimurium definitive type 104 (DT104), a lineage within *S*. Typhimurium that is known for its typical ampicillin-, chloramphenicol-, streptomycin-, sulfonamide- and tetracycline-resistant (ACSSuT) phenotype, although its antimicrobial resistance (AMR) profile may vary [14]. Multidrug-resistant (MDR) DT104 is predicted to have emerged circa 1972 [14] and rapidly disseminated around the world in the following decades [14–16], culminating in a global epidemic among animals and humans in the 1990s [14–16]. However, despite its rapid global dissemination, DT104 does not appear to be more virulent than non-DT104 *S*. Typhimurium in a classical mouse model [17].

In addition to its characteristic MDR phenotype, DT104 is notable for its ability to produce ArtAB, a pertussis-like toxin that catalyses ADP-ribosylation of host G proteins [18–20]. Treatment of various cell lines with purified ArtAB from DT104 recapitulates some of the phenotypes established for pertussis toxin cytotoxicity [21–23], such as the characteristic ‘cell clustering’ phenotype in CHO-K1 cells [24], increased levels of intracellular cAMP in RAW 264.7 macrophage-like cells [19] and increased serum insulin levels (e.g. insulinaemia); furthermore, intraperitoneal injection of purified toxin in neonatal mice was fatal [19].

**Fig. 1.**
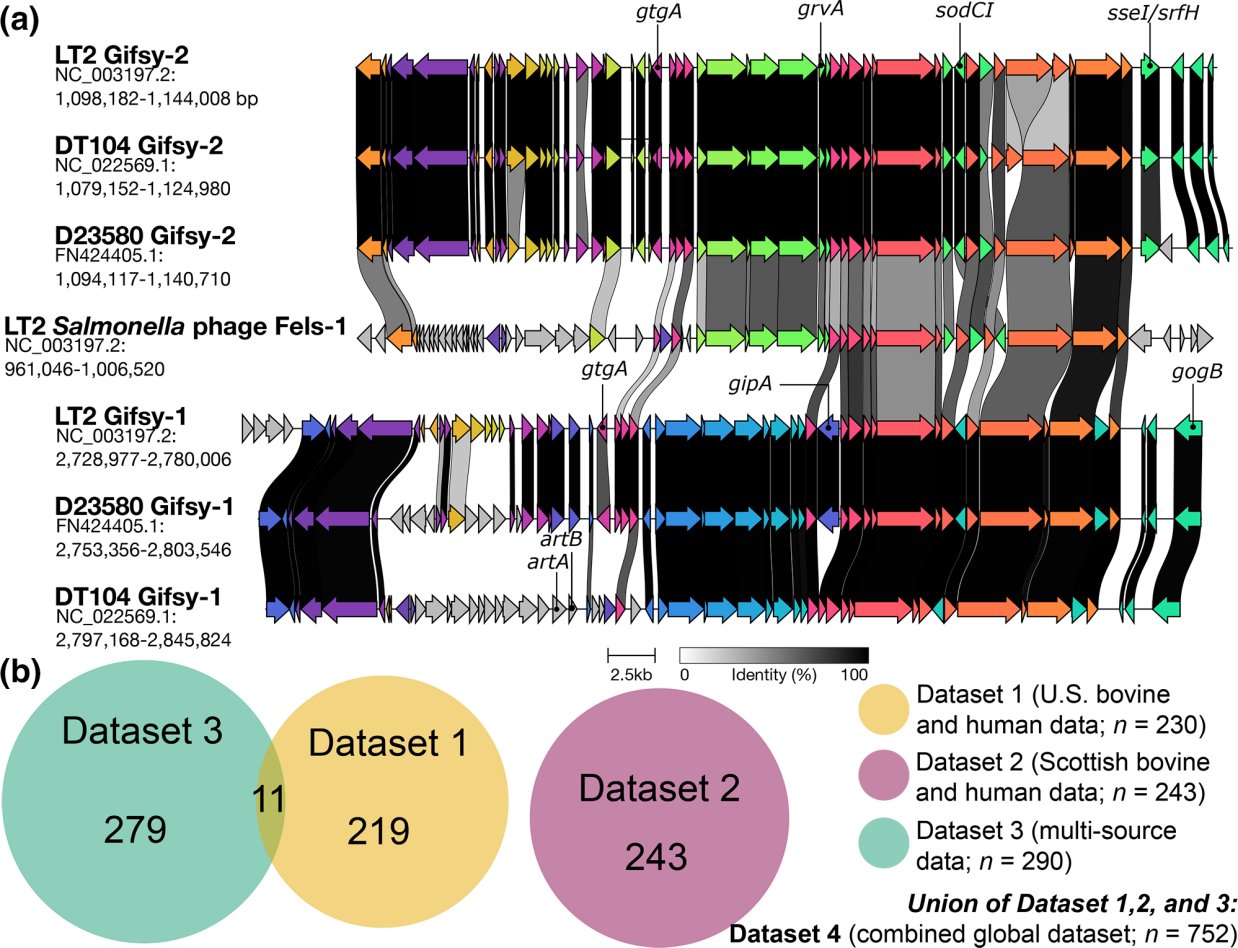
(**a**) Selected prophages that share homology with prophage Gifsy-1, as described in *Salmonella* Typhimurium strains (**i**) LT2, (ii) DT104 and (iii) D23580. Prophage regions were acquired from the PHASTER database and annotated using Prokka. The program clinker was used to compare prophage regions using default settings. Arrows correspond to ORFs, with greyscale links denoting the percentage amino acid identity shared between corresponding ORFs. Selected Gifsy-1 and Gifsy-2 virulence factors [30] are annotated. To view a similar plot constructed using all PHASTER prophage regions in LT2, DT104 and D23580, see Fig. S1. (**b**) Venn diagram showing the relationship between DT104 complex datasets used in this study. Numbers denote the number of genomes within a given dataset or subset of a dataset. For an extended version of this figure, see Fig. S2. For a flow chart with detailed descriptions of the datasets used in this study, see Fig. S3.

The genes *artAB,* which encode ArtAB, have been detected in representative strains of at least 88 *Salmonella* serotypes [25], and previous studies have found that *artAB* can be encoded by prophages (e.g. Gifsy-1, PhInv-1b) [18–20, 26–28]. Within *S*. Typhimurium specifically, *artAB* shares a strong association with DT104 relative to other *S*. Typhimurium lineages: while typically absent in most non-DT104 *S*. Typhimurium strains, the overwhelming majority of DT104 possess *artAB* [19]. In DT104 specifically, *artAB* has been identified within prophage Gifsy-1 (Figs 1a and S1, available in the online version of this article) [11, 20, 26]. Gifsy-1 has been detected in numerous *Salmonella* serotypes [29] and has been shown to harbour virulence factors [30] such as *gogB*, *gipA* and *gtgA* [30–32]; however, *artAB*-harbouring Gifsy-1 has been proposed to be a characteristic feature of DT104 (Figs 1a and S1) [11].

Despite their strong association, DT104 strains that lack *artAB* and, thus, the ability to produce ArtAB toxin, have been described on occasion (referred to hereafter as ‘*artAB*-negative’ strains) [19, 33]. We identified three *artAB*-negative DT104 complex strains in a previous study of *S*. Typhimurium from cattle and humans in New York State (USA) [33]. Because *artAB* tends to be prophage-encoded [18–20, 26, 27], we hypothesized that it may be possible for *artAB* to be gained or lost as an *artAB*-harbouring prophage integrates or excises from a genome, or via HGT within an integrated prophage. However, the extent to which any of these scenarios occur is unknown. Using (i) 230 human- and bovine-associated DT104 complex genomes collected across the USA, plus (ii) 752 DT104 complex genomes collected from a range of sources worldwide, we provide large-scale insight into the dynamics of *artAB* acquisition and loss within the DT104 complex.

## Methods

### Acquisition of USA human- and bovine-associated DT104 complex genomic data and metadata

In a previous study of human- and bovine-associated *S*. Typhimurium from New York State, we identified three closely related, *artAB*-negative DT104 complex genomes from both humans and cattle (out of 14 total DT104 complex genomes from humans and cattle in New York State) [33]. Thus, as a first evaluation of *artAB* presence and absence in the DT104 complex, we compiled a set of DT104 complex genomes from humans and cattle across the USA [referred to hereafter as ‘Dataset 1 (U.S. bovine and human data)’; Figs 1b, S2 and S3].

To construct Dataset 1 (U.S. bovine and human data), we first collected genomic data derived from 14 human- and bovine-associated DT104 complex isolates from New York State, which we had sequenced in a previous study (members of the *S*. Typhimurium Lineage III cluster described in figs S2 and S5 of Carroll *et al*.) [33]. We then aggregated these 14 New York State genomes with 223 human- and bovine-associated DT104 complex genomes from across the USA, as described previously [33]. Briefly, paired-end Illumina short reads associated with 223 *S*. Typhimurium genomes meeting the following criteria were downloaded from the National Center for Biotechnology Information (NCBI) Sequence Read Archive (SRA; https://www.ncbi.nlm.nih.gov/sra, accessed 29 November 2018), using accession numbers provided by Enterobase and the SRA Toolkit version 2.9.3 [34–37]: (i) genomes were serotyped as *S*. Typhimurium *in silico* using the implementation of SISTR [38] in Enterobase; (ii) the country of isolation was the USA; (iii) the isolation source was reported as either ‘Human’ or ‘Bovine’ in the ‘Source Niche’ and ‘Source Type’ fields in Enterobase, respectively; (iv) genomes had an isolation year reported in Enterobase; (v) using RhierBAPS [39], genomes were assigned to the DT104 complex, a well-supported cluster within the larger bovine- and human-associated USA *S*. Typhimurium phylogeny, which clustered among known DT104 genomes from other countries (see figs S2 and S5 of Carroll, *et al*.) [33].

Trimmomatic version 0.36 [40] was used to trim low-quality bases and Illumina adapters from all read sets using the default settings for paired-end reads, and SPAdes version 3.13.0 [41] was used to assemble all genomes using default settings plus the ‘careful’ option. FastQC version 0.11.5 [42] and QUAST version 4.0 [43] were used to assess the quality of each read pair set and assembly, respectively, and MultiQC version 1.6 [44] was used to aggregate all FastQC and QUAST results. Trimmed paired-end read sets/assemblies that were flagged by MultiQC as meeting any of the following conditions were excluded: (i) Illumina adapters present after trimming (*n*=2), (ii) an abnormal per sequence GC content distribution (*n*=3), (iii) an assembly with over 200 contigs (*n*=11) and (iv) a sequence quality histogram flagged as poor quality (*n*=2). After excluding genomes that met these conditions, a set of 219 DT104 complex genomes was produced (Figs 1b, S2 and S3).

**Fig. 2.**
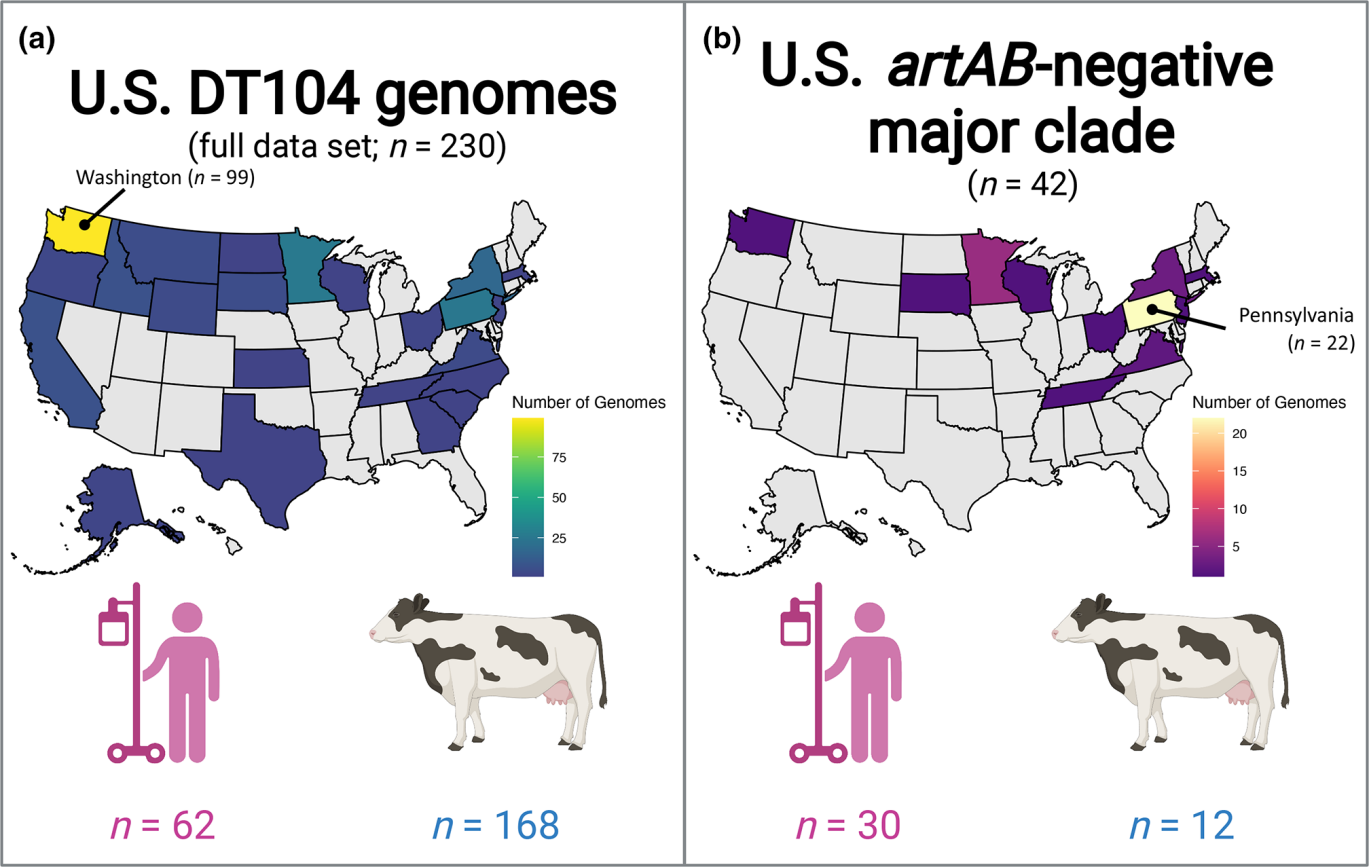
Geographical and source origins (i.e. human or bovine) of (**a**) all 230 human- and bovine-associated U.S. DT104 complex genomes queried in this study [i.e. Dataset 1 (U.S. bovine and human data)], and (**b**) 42 Gifsy-1/*artAB*/*gogB*-negative genomes assigned to the U.S. *artAB*-negative major clade. States shown in grey did not contribute any genomes to the respective data set. The state that contributed the most genomes to its respective data set is labelled. The figure was created using BioRender (https://biorender.com/) and the ‘plot_usmap’ function in the usmap version 0.6.0 R package [147].

Finally, the 219 U.S. human- and bovine-associated DT104 complex genomes identified here were supplemented with 11 U.S. bovine- and human-associated DT104 genomes from a previous study [14], which did not have metadata available in Enterobase at the time and were thus not included in the initial set of 219 bovine- and human-associated U.S. DT104 complex genomes. Overall, the search conducted here produced a set of 230 bovine- and human-associated U.S. DT104 complex genomes, which were used in subsequent steps [i.e. Dataset 1 (U.S. bovine and human data); Figs 1b, 2a, S2 and S3, Tables S1 and S2).

### *In silico* detection of prophages, antimicrobial resistance genes, plasmid replicons and virulence factors

To identify putative prophage regions in all 230 genomes in Dataset 1 (U.S. bovine and human data), each assembly was submitted to the PHASTER web server (https://phaster.ca/) via the URL API [45, 46], with the ‘contigs’ option set to ‘1’ (suggested by PHASTER for multi-contig files in multi-FASTA format, https://phaster.ca/instructions#urlapi, accessed 13 May 2023; Table S3). To compare prophage regions identified in Dataset 1 (U.S. bovine and human data) genomes to previously described prophages in well-characterized *S*. Typhimurium strains, prophages in the following *S*. Typhimurium strains were obtained from the PHASTER prophage database (accessed 18 September 2020): (i) LT2 (NCBI Nucleotide accession NC_003197.2), (ii) DT104 (NCBI Nucleotide accession NC_022569.1), (iii) D23580 (NCBI Nucleotide accession FN424405.1) and (iv) SL1344 (NCBI Nucleotide accession NC_016810.1; Figs 1a and S1). All prophage regions were annotated using Prokka version 1.14.6 [47], using default settings and the ‘Viruses’ kingdom database. The resulting GFF and FNA files produced by Prokka were supplied to clinker version 0.0.26 [48], which was used to perform pairwise alignments of all genes within prophage regions using default settings.

ABRicate version 0.8 [49] was used to detect antimicrobial resistance (AMR) genes, plasmid replicons and virulence factors in each assembled DT104 complex genome using NCBI’s National Database of Antibiotic Resistant Organisms (NDARO) [50], the PlasmidFinder database [51] and the Virulence Factor Database (VFDB) [52], respectively, using minimum nucleotide identity and coverage thresholds of 75 and 50 %, respectively (all databases accessed 10 December 2020; Table S3). The aforementioned ABRicate analyses were repeated, using a minimum coverage threshold of 0 % (e.g. to confirm that virulence factors discussed in this paper were absent from genomes in which they were not initially detected).

Each assembled genome was additionally queried for the presence of selected virulence factors, which have previously been associated with prophages in *Salmonella* [30]: (i) *artAB* (NCBI Nucleotide accession AB104436.1), (ii) *gogA* [European Nucleotide Archive (ENA) accession EAA7850902.1], (iii) *gtgA* (ENA accession PVI70081.1) and (iv) *gipA* (ENA accession CAI93790.1). Assembled genomes were queried for selected virulence factors using the command-line implementation of nucleotide blast (blastn) version 2.11.0 [53], using default settings plus a minimum coverage threshold of 40 % (Table S3). To confirm that the aforementioned genes were absent from genomes in which they were not initially detected, all genomes were queried again (i) as described above, with the coverage threshold lowered to 0 %; and (ii) using translated nucleotide blast (tblastx; Tables S4 and S5). ARIBA version 2.14.6 [54] was used to further confirm *artAB* and *gogB* presence/absence in all genomes with associated paired-end Illumina reads (Table S6 and Supplementary Text).

### Variant calling and maximum likelihood phylogeny reconstruction within Dataset 1 (U.S. bovine and human data)

Core SNPs were identified among all 230 genomes within Dataset 1 (U.S. bovine and human data), using the default pipeline implemented in Snippy version 4.6.0 (https://github.com/tseemann/snippy; Tables S1 and S2 and Supplementary Text) [55–68]. The closed DT104 chromosome (NCBI Nucleotide accession NC_022569.1) was used as a reference, and core SNPs identified in regions of the DT104 chromosome predicted to belong to phages were masked (Supplementary Text). Gubbins version 2.4.1 [69] was used to identify and remove recombination events in all genomes using default settings, and snp-sites was used to query the resulting recombination-free alignment for core SNPs (i.e. using the ‘-c’ option).

A maximum likelihood (ML) phylogeny was constructed with IQ-TREE version 1.5.4 [70], using (i) the resulting core SNPs as input, (ii) the optimal nucleotide substitution model selected using ModelFinder [71, 72], (iii) an ascertainment bias correction to account for the use of solely variant sites and (iv) 1000 replicates of the ultrafast bootstrap approximation (Supplementary Text) [73, 74]. TempEst version 1.5.3 [75] was used to assess the temporal structure of the resulting unrooted ML phylogeny, using the best-fitting root and the *R* ^2^ function (*R* ^2^=0.33, slope=3.05×10^−7^ substitutions per site per year, *x*-intercept=1988.1). The unrooted ML phylogeny was additionally rooted and time scaled using LSD2 version 1.4.2.2 [76], using tip dates corresponding to the year of isolation reported for each genome (Supplementary Text). The resulting rooted, time-scaled ML phylogeny was viewed using FigTree version 1.4.4 [77] (Supplementary Data).

### Dataset 1 (U.S. bovine and human data) Bayesian time-scaled phylogeny reconstruction

In addition to constructing a time-scaled ML phylogeny [see section ‘Variant calling and maximum likelihood phylogeny construction within Dataset 1 (U.S. bovine and human data) above], a Bayesian approach was additionally employed to construct a time-scaled phylogeny, using a subset of 146 Dataset 1 (U.S. bovine and human data) genomes (Fig. S4 and Supplementary Text). All aforementioned SNP calling and ML phylogeny reconstruction steps were repeated within the 146-genome Dataset 1 (U.S. bovine and human data) subset, and the resulting ML phylogeny was time-scaled using TempEst and LSD2 as described above [see section ‘Variant calling and maximum likelihood phylogeny construction within Dataset 1 (U.S. bovine and human data)’ above; Table S7, Supplementary Data, and Supplementary Text].

**Fig. 3.**
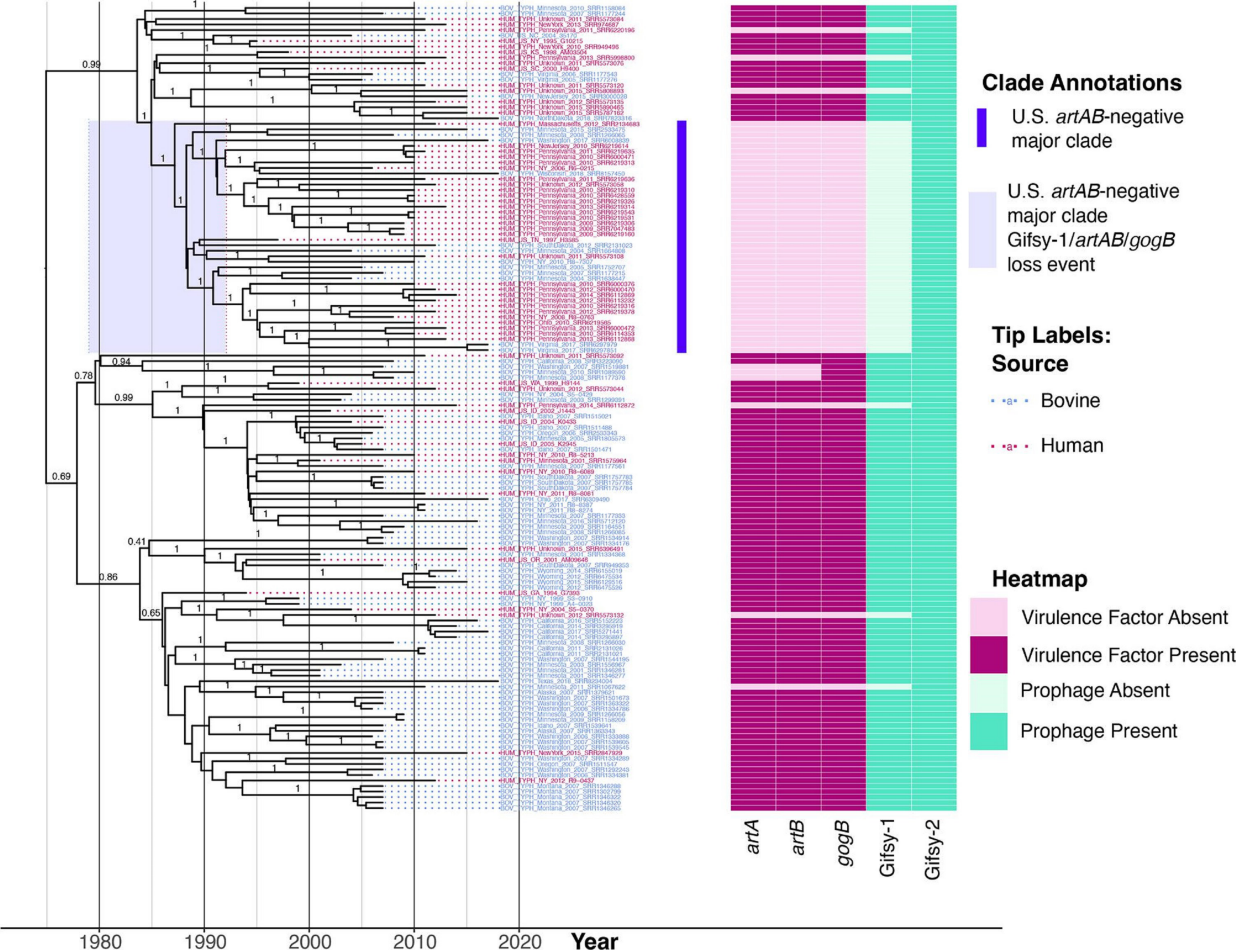
Bayesian time-scaled phylogeny reconstructed using 146 human- and bovine-associated DT104 complex genomes collected in the USA [i.e. a subset of genomes from Dataset 1 (U.S. bovine and human data)]. Tip label colours denote the isolation source reported for each genome (human or bovine in pink and blue, respectively). The heatmap to the right of the phylogeny denotes the presence and absence of (**i**) selected virulence factors (dark and light pink, respectively) and (ii) prophages (dark and light green, respectively). The U.S. *artAB*-negative major clade is denoted by the bright purple bar; light purple shading around the node of the U.S. *artAB*-negative major clade denotes the 95 % highest posterior density (HPD) interval, in which Gifsy-1/*artAB*/*gogB* were predicted to have been lost. The phylogeny was reconstructed and rooted using beast2. Time in years is plotted along the *x*-axis, while branch labels correspond to posterior probabilities of branch support (selected for readability). For extended versions of this figure, see Figs S6–S8.

**Fig. 4.**
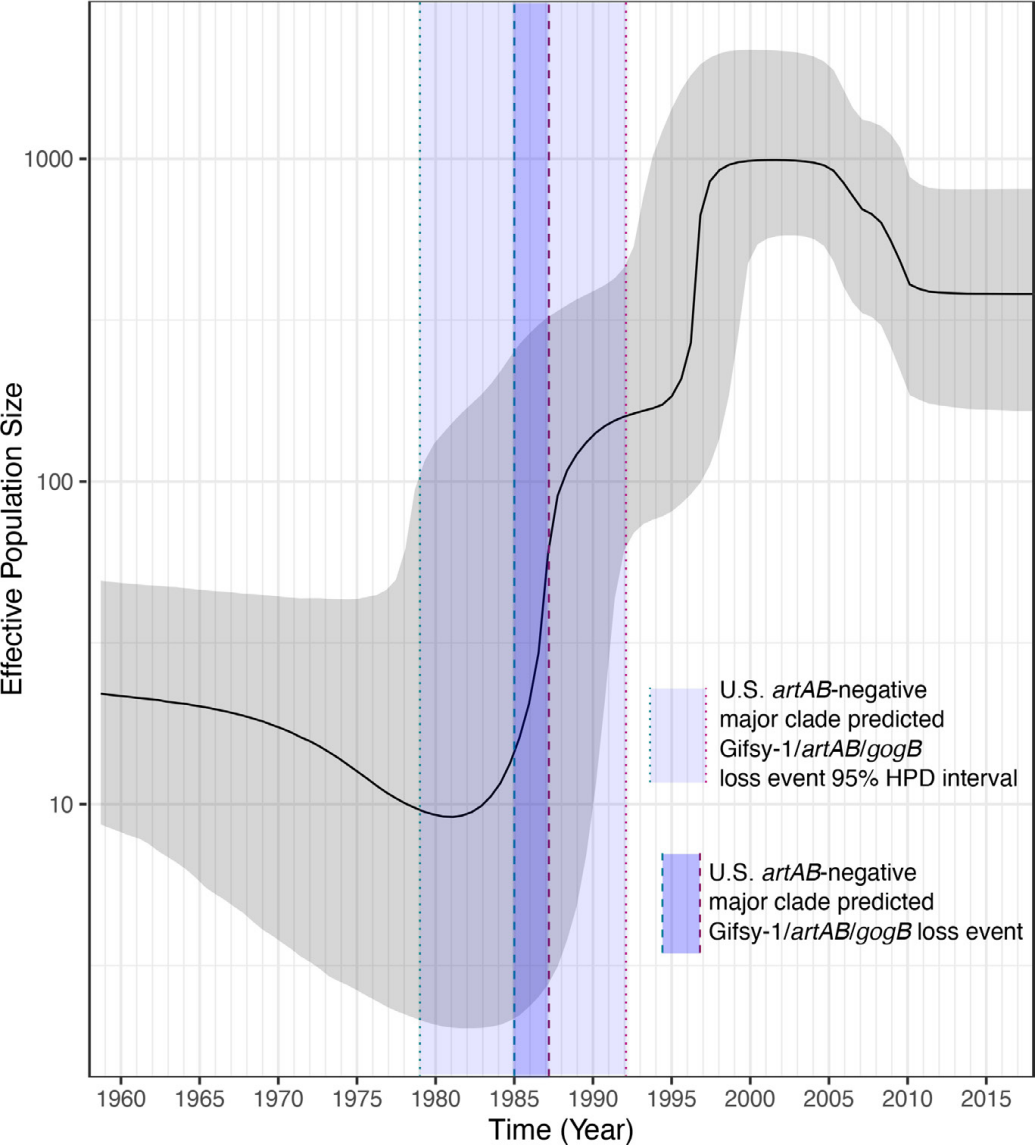
Coalescent Bayesian Skyline plot constructed using 146 U.S. bovine- and human-associated *S*. Typhimurium DT104 complex genomes [i.e. a subset of genomes from Dataset 1 (U.S. bovine and human data)]. Effective population size and time in years are plotted along the *y*- and *x*-axes, respectively. The median effective population size estimate is denoted by the solid black line, with upper and lower 95 % HPD interval bounds denoted by grey shading. The interval shaded in light blue and bounded by dashed vertical lines denotes the time interval in which Gifsy-1/*artAB*/*gogB* were predicted to have been lost by the common ancestor of the U.S. *artAB*-negative major clade (corresponding to the years 1985.0 and 1987.2, denoted by turquoise and pink dashed lines, respectively). The dotted turquoise and pink vertical lines correspond to the 95 % HPD interval lower and upper bounds for Gifsy-1/*artAB*/*gogB* loss among members of the U.S. *artAB*-negative major clade (corresponding to the years 1979.0 and 1992.1, respectively).

The program beast2 version 2.5.1 [78, 79] was used to reconstruct a tip-dated phylogeny, using core SNPs detected among the 146-genome Dataset 1 (U.S. bovine and human data) subset as input (Supplementary Text). An initial clock rate of 2.79×10^−7^ substitutions per site per year [14] was used, along with an ascertainment bias correction to account for the use of solely variant sites [80]. The program bmodeltest [81] was used to infer a substitution model using Bayesian model averaging, with transitions and transversions split. A relaxed lognormal molecular clock [82] and a coalescent Bayesian skyline population model [83] were used, as these models have been selected as the optimal clock/population model combination for DT104 previously [14] (Supplementary Text). Five independent beast2 runs (i.e. beast2 runs with different random seeds) were performed, using chain lengths of at least 100 million generations, sampling every 10 000 generations. LogCombiner-2 was used to aggregate the resulting log and tree files with 10 % of the states treated as burn-in, and TreeAnnotator-2 was used to produce a maximum clade credibility (MCC) tree using Common Ancestor node heights (Fig. S5, Table S8 and Supplementary Data). The resulting phylogenies were displayed and annotated using R version 4.1.2 (Figs 3, 4 and S6–S9, and Supplementary Text) [84–89].

### The *artAB* ancestral state reconstruction for Dataset 1 (U.S. bovine and human data)

To estimate ancestral character states of internal nodes in the Dataset 1 (U.S. bovine and human data) phylogeny as they related to *artAB* presence/absence (i.e. whether a node in the tree represented an ancestor that was more likely to be *artAB*-positive or *artAB*-negative), the presence or absence of *artAB* within each genome was treated as a binary state (see section ‘*In silico* detection of prophage, antimicrobial resistance genes, plasmid replicons and virulence factors’ above). *artAB* ancestral state reconstruction runs were performed using the beast2 time-scaled Bayesian Dataset 1 (U.S. bovine and human data) phylogeny as input [*n*=146 genomes; see section ‘Dataset 1 (U.S. bovine and human data) Bayesian time-scaled phylogeny reconstruction’ above; Supplementary Data and Supplementary Text]. Stochastic character maps were simulated on the phylogeny using the make.simmap function in the phytools version 1.0–1 R package [90] and the all-rates-different (ARD) model in the ape version 5.6-1 package [91, 92]; two root node priors were tested (Supplementary Text). The resulting phylogenies (one for each root node prior) were plotted using the densityMap function in the phytools R package (Figs S10–S12, Supplementary Data).

### Pan-genome characterization of Dataset 1 (U.S. bovine and human data)

Prokka version 1.13.3 [47] was used to annotate all 230 genomes within Dataset 1 (U.S. bovine and human data), using the ‘Bacteria’ database and default settings (Tables S1 and S2). GFF files produced by Prokka were supplied as input to Panaroo version 1.2.7 [93], which was used to identify core- and pan-genome orthologous gene clusters among the 230 Dataset 1 (U.S. bovine and human data) genomes (Supplementary Text) [94, 95]. The LSD2 time-scaled ML phylogeny for Dataset 1 (U.S. bovine and human data) [see section ‘Variant calling and maximum likelihood phylogeny construction within Dataset 1 (U.S. bovine and human data)’ above] was supplied as input to Panaroo’s ‘panaroo-img’ and ‘panaroo-fmg’ commands, which were used to estimate the pan-genome size under the Infinitely Many Genes (IMG) [96, 97] and Finite Many Genes (FMG) models (with 100 bootstrap replicates) [98], respectively (Fig. S13).

Reference pan-genome coding sequences (CDS) identified by Panaroo underwent functional annotation using the eggNOG-mapper version 2 webserver (http://eggnog-mapper.embl.de/; accessed 24 July 2022) using default settings [99, 100]. The ‘table’ function in R was used to identify genes associated with (i) prophage Gifsy-1 presence/absence (Table S9) and (ii) clade membership (Table S10); the ‘fisher.test’ function in R’s stats package was used to conduct two-sided Fisher’s exact tests, and the ‘p.adjust’ function was used to control the false discovery rate (FDR; i.e., p.adjust method=‘fdr’) [101].

### Genome-wide identification of host-associated orthologous gene clusters for Dataset 1 (U.S. bovine and human data)

The treeWAS version 1.0 R package [102] was used to identify potential orthologous gene cluster–host associations among the 230 human- and bovine-associated U.S. DT104 complex genomes in Dataset 1 (U.S. bovine and human data) (i.e. whether an orthologous gene cluster identified with Panaroo was human- or bovine-associated while accounting for population structure; Supplementary Text). No orthologous gene clusters were found to be significantly associated with isolation source via any of the treeWAS association tests (FDR-corrected *P*>0.10).

### Acquisition of global DT104 complex genomic data and metadata

To compare the 230 U.S. human- and bovine-associated DT104 complex genomes in Dataset 1 (U.S. bovine and human data) to a larger set of DT104 complex genomes from numerous sources worldwide, genomic data associated with the following studies were downloaded via Enterobase: (i) 243 bovine- and human-associated DT104 isolates from a study of between-host transmission within Scotland [103] [referred to hereafter as ‘Dataset 2 (Scottish bovine and human data)’; Supplementary Text]; (ii) 290 DT104 isolates from a variety of sources and countries from a study describing the global spread of DT104 [14] [referred to hereafter as ‘Dataset 3 (multi-source data)’; 11 of the 290 genomes were isolated from cattle and humans in the USA and thus had also been included in Dataset 1 (U.S. bovine and human data), Figs 1b, S2 and S3, Table S1 and Supplementary Text].

The following datasets were aggregated to create a final set of 752 DT104 complex genomes derived from numerous countries and isolation sources, which was used in subsequent steps [referred to hereafter as ‘Dataset 4 (combined global dataset)’; Figs 1b, S2 and S3, Tables S1 and S2]: (i) Dataset 1 (U.S. bovine and human data) (*n*=230 DT104 complex genomes), (ii) Dataset 2 (Scottish bovine and human data) (*n*=243 DT104 genomes) and (iii) Dataset 3 (multi-source data) [*n*=290 DT104 genomes, including 11 genomes that were part of Dataset 1 (U.S. bovine and human data)]. quast version 4.5 was used to assess the quality of all 752 genomes in Dataset 4 (combined global dataset) (Tables S1 and S2). Prophage, AMR genes, plasmid replicons and virulence factors were detected in all 752 Dataset 4 (combined global dataset) genomes as described above (see section ‘*In silico* detection of prophage, antimicrobial resistance genes, plasmid replicons, and virulence factors’ above; Tables S3–S5).

### Variant calling and ML phylogeny construction within Dataset 4 (combined global dataset)

To identify core SNPs present in all 752 DT104 complex genomes within Dataset 4 (combined global dataset), Parsnp and HarvestTools version 1.2 [104] were used, as Parsnp easily scales to large data sets (Tables S1 and S2) [104]. Assembled genomes were used as input for Parsnp, along with the closed DT104 chromosome as a reference (NCBI Nucleotide accession NC_022569.1) and Parsnp’s implementation of PhiPack [105] to filter recombination.

**Fig. 5.**
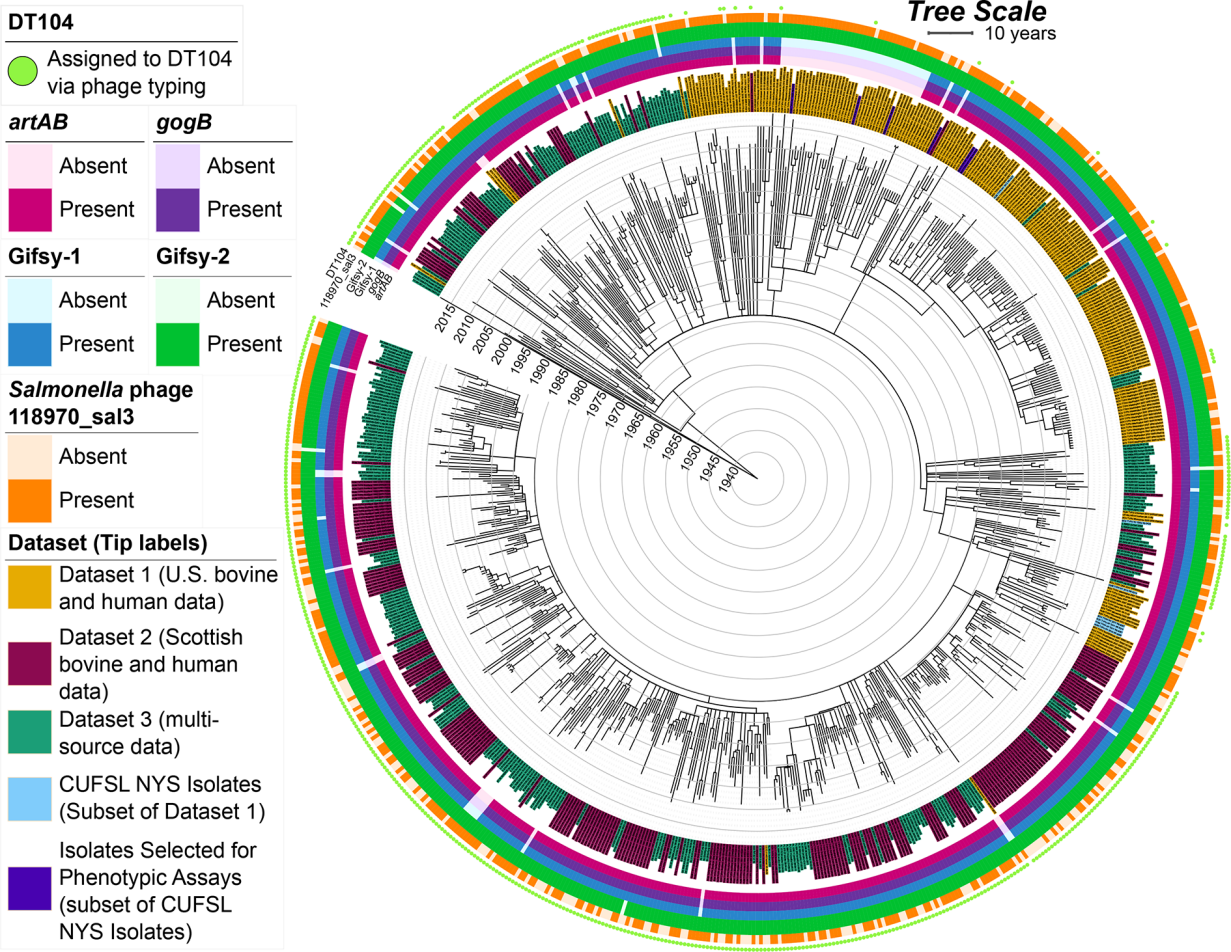
Time-scaled ML phylogeny reconstructed using 752 DT104 complex genomes [i.e. Dataset 4 (combined global dataset), the union of Dataset 1 (U.S. bovine and human data), Dataset 2 (Scottish bovine and human data) and Dataset 3 (multi-source data)]. Tip label colours denote the dataset or dataset subset with which each genome is affiliated [‘Dataset’; 11 genomes within the intersection of Dataset 1 (U.S. bovine and human data) and Dataset 3 (multi-source data) were coloured as Dataset 1 (U.S. bovine and human data)]. The heatmap encompassing the phylogeny denotes the presence and absence of selected virulence factors and intact prophage [identified via nucleotide blast (blastn; default settings, with no minimum identity or coverage threshold employed) and PHASTER, respectively]. Bright green circles in the outermost ring surrounding the phylogeny denote genomes reportedly assigned to DT104 using phage typing (‘DT104’). The ML phylogeny was reconstructed using IQ-TREE and rooted and time-scaled using LSD2. Branch lengths are reported in years. For an extended version of this figure, see Fig. S14.

Core SNPs detected among all 752 assembled genomes within Dataset 4 (combined global dataset) were supplied as input to IQ-TREE version 1.5.4, which was used to reconstruct an ML phylogeny as described above; the resulting ML phylogeny was rooted and time-scaled using LSD2 as described above [see section ‘Variant calling and maximum likelihood phylogeny reconstruction within Dataset 1 (U.S. bovine and human data)’ above; Supplementary Data and Supplementary Text]. The resulting LSD2 time-scaled ML phylogeny was annotated using the Interactive Tree of Life (iTOL) version 6 webserver (https://itol.embl.de/, accessed 7 March 2022; Figs 5 and S14, Supplementary Data) [106]. The LSD2 time-scaled ML phylogeny for Dataset 4 (combined global dataset) was further used for *artAB* presence/absence ancestral state reconstruction as described above [see section ‘*artAB* ancestral state reconstruction for Dataset 1 (U.S. bovine and human data)’ above; Fig. S15].

### Pan-genome characterization of Dataset 4 (combined global dataset)

Pan-genome analyses were carried out for Dataset 4 (combined global dataset) as described above [see section ‘Pan-genome characterization of Dataset 1 (U.S. bovine and human data)’ above; Tables S1 and S2, Supplementary Text]. The pan-genome size for Dataset 4 (combined global dataset) was estimated using Panaroo’s ‘panaroo-img’ and ‘panaroo-fmg’ commands, using the LSD2 time-scaled ML phylogeny for Dataset 4 (combined global dataset) as input [see section ‘Variant calling and maximum likelihood phylogeny construction within Dataset 4 (combined global dataset)’ above; Fig. S13].

### Strain selection for phenotypic stress assays

Phenotypic stress assays (discussed in detail in the sections below) were used to compare (i) bovine- and human-associated, Gifsy-1/*artAB*/*gogB*-positive U.S. DT104 complex strains to (ii) bovine- and human-associated, Gifsy-1/*artAB*/*gogB*-negative U.S. DT104 complex strains. Thus, additional analyses were performed to identify the most closely related, Gifsy-1/*artAB*/*gogB*-positive and -negative strains available in the Cornell University Food Safety Laboratory (CUFSL) culture collection for phenotypic testing (Figs S2 and S3, Table S2, and Supplementary Text) [107–109]. Overall, the CUFSL DT104 complex genomes differed little in terms of their core and pan-genome compositions (Fig. S16 and Supplementary Text).

Considering both (i) core- and pan-genome similarities between all 13 available CUFSL DT104 complex genomes (Fig. S16 and Table S2), as well as (ii) Gifsy-1/*artAB*/*gogB* presence and absence, we selected six closely related, CUFSL DT104 complex strains to undergo phenotypic characterization: three Gifsy-1/*artAB*/*gogB*-positive strains and three Gifsy-1/*artAB*/*gogB*-negative strains (Figs 5 and S16, Table S11, and Supplementary Text). All three Gifsy-1/*artAB*/*gogB*-negative strains were members of the U.S. *artAB*-negative major clade (discussed in detail in the ‘Results’ section below; Fig. 5 and Table S11). All six selected strains had been isolated from humans or cattle in New York State and were part of Dataset 1 (U.S. bovine and human data) (Figs S2 and S3, and Tables S2 and S11).

### Phenotypic assays

Six carefully selected, human- and bovine-associated DT104 complex strains from New York State, which were available to us in the CUFSL culture collection, were characterized using phenotypic assays (see section ‘Strain selection for phenotypic stress assays’ above; Table S11). All strain stocks were maintained in CRYOBANK tubes (Mast) at −80 °C. Strains were streaked out from stocks on tryptic soy agar (TSA; Merck) and incubated overnight at 37 °C. Single colonies from those plates were inoculated in 5 ml of tryptic soy broth (TSB; Merck) and incubated for 16–18 h at 37 °C with shaking at 200 r.p.m. The resulting overnight cultures were diluted 1:100 into 5 ml of fresh, pre-warmed TSB, followed by incubation at 37 °C with shaking at 200 r.p.m. to allow cultures to reach mid-log phase (defined as OD_600_ of 0.4; 1–2×10^8^ c.f.u. ml^−1^). These cultures were used as input into three different phenotypic assays (exposure to ruminal fluid, acid stress and bile stress; discussed in detail below). Bacterial enumeration before and after stress exposure was performed by direct colony counts of tilt plates according to Kühbacher *et al*. [110].

To evaluate exposure to ruminal fluid (RF), approximately 2 litres of RF was acquired from a Jersey cow with a ruminal fistula on each experimental day prior to the experiments (the same collection time was used for each experiment). The RF was immediately filtered through a cellulose filter (Labsolute Type 80; Th. Geyer) to remove any large debris, and the pH was measured, ranging from 7.20 to 7.62. Mid-log phase cultures were prepared and inoculated into the RF at two different concentrations. Culture suspensions of 100 µl were inoculated into 5 ml of the RF at final concentrations of 10^8^ (high) and 10^5^ (low) c.f.u. ml^–1^ and incubated for 1 h at 37 °C without shaking, with enumeration by direct colony counting on XLT-4 agar (Oxoid) prior and after RF exposure (Table S12). The absence of *Salmonella* in the RF at the start of the experiments was confirmed by plating on XLT-4 agar.

Acid stress resistance of the different strains at pH 3.5 with and without prior adaption was tested using an adopted protocol from Horlbog *et al*. [111]. To carry out the acid stress assay, the pH of the TSB was adjusted with hydrochloric acid solution (1 and 6 M HCl; Merck) immediately prior to the experiment. Aliquots (1 ml) of mid-log phase cultures were transferred to reaction tubes and centrifuged at 14 000 ***g*** for 10 min. For the non-adapted acid stress experiments, the pellets were resuspended in 1 ml TSB pH 3.5 and incubated for 1 h at 37 °C without shaking. For acid adaption, 1 ml of the same cultures was pelleted, resuspended in 1 ml TSB adjusted to pH 5.5 and incubated for 1 h at 37 °C (without shaking). The cultures were then centrifuged again, resuspended in 1 ml TSB pH 3.5 and incubated for 1 h at 37 °C without shaking. Bacteria enumeration was performed before and after the 1 h of incubation at pH 3.5 (Table S13).

Susceptibility to bile salts (cholic acid and deoxycholic acid in a mixture of 1 : 1, Bile Salts No.3; Thermo Fisher Scientific) was tested in two different concentrations: 14.5 mmol l^–1^ corresponding to 0.6 % [112] and 26.0 mmol l^–1^ corresponding to 1.1 % [113] were chosen to represent reasonable physiological states in the duodenum. Bile salts were added, and the pH of the TSB was adjusted to 5.5 (TSB-bile) immediately prior to the experiment. Mid-log phase cultures were centrifuged, resuspended in TSB-bile, incubated for 1 h at 37 °C without shaking, and enumerated by direct colony counting prior and after bile exposure (Table S14).

For each stress assay, base-10 logarithmic fold change (FC) values were calculated as follows: FC=log c.f.u. g^−1^ at the start of the experiments – log c.f.u. g^−1^ after the stress assay. ANOVAs for interpretation of the phenotypic assays were conducted using the ‘aov’ function in R’s ‘stats’ package, with the FC values for the respective assay treated as a response. Figures were designed using the ggplot2 package.

## Data availability

Strain metadata, genome quality metrics and Enterobase accession numbers for all publicly available genomes queried in this study are available in Table S1. Strain metadata, genome quality metrics, CUFSL IDs [107] and NCBI BioSample accession numbers [114] for the 13 New York State CUFSL DT104 complex strains queried in this study (including those queried via phenotypic assays) are available in Table S2. LSD2 results [for Dataset 1 (U.S. bovine and human data) and Dataset 4 ([combined global dataset)] and beast2 results [for subsets of Dataset 1 (U.S. bovine and human data)] are available as Supplementary Data.

## Results

### Human- and bovine-associated DT104 complex strains from the USA harbour *artAB* on prophage Gifsy-1

Within the set of 230 human- and bovine-associated U.S. DT104 complex genomes [i.e. Dataset 1 (U.S. bovine and human data); Fig. 2a] [33], *artAB* was present in over 75 % of genomes (177 of 230, 77.0 %; Figs 3 and S6–S8, Table 1). Presence and absence of *artAB* was strongly associated with the presence and absence of anti-inflammatory effector *gogB* [two-sided Fisher’s Exact Test (FET) raw *P*<2.2×10^−16^, odds ratio (OR)=∞], as co-occurrence was observed in all 177 *artAB*-harbouring Dataset 1 (U.S. bovine and human data) genomes (100.0 %; Figs 3 and S6–S8, Table 1). Additionally, within Dataset 1 (U.S. bovine and human data), *artAB* and *gogB* presence was strongly associated with the presence of prophage Gifsy-1 (NCBI Nucleotide accession NC_010392.1; two-sided FET raw *P*<2.2×10^−16^, OR=∞; Figs 3 and S6–S8, Table 1).

**Table 1.** Presence and absence of *artAB*, *gogB* and Gifsy-1 among four DT104 complex genome datasets queried in this study

Data set(s)	Host(s)	Total no. of genomes	*artAB* present (%)*	*gogB* present (%)*	Gifsy-1 present (%)†
Dataset 1 (U.S. bovine and human data)‡					
	All	230	177 (77.0 %)	180 (78.3 %)	180 (78.3 %)
	Bovine	168	150 (89.3 %)	153 (91.1 %)	153 (91.1 %)
	Human	62	27 (43.5 %)	27 (43.5 %)	27 (43.5 %)
Dataset 2 (Scottish bovine and human data)§					
	All	243	240 (98.8 %)	240 (98.8 %)	144 (59.3 %)
	Bovine	82	82 (100.0 %)	82 (100.0 %)	48 (58.5 %)
	Human	161	158 (98.1 %)	158 (98.1 %)	96 (59.6 %)
Dataset 3 (multi-source data)||					
	All	290	271 (93.4 %)	271 (93.4 %)	265 (91.4 %)
Dataset 4 (combined global dataset)¶					
	All	752	678 (90.2 %)	681 (90.6 %)	579 (77.0 %)

*Identified using nucleotide blast (blastn; default settings, with no minimum identity or coverage threshold employed).

†Identified using the PHASTER webserver; Gifsy-1 regions annotated as ‘intact’, ‘incomplete’ or ‘questionable’ were considered to be ‘present’ in a genome.

‡Refers to the set of 230 U.S. human- and bovine-associated DT104 complex genomes identified and aggregated here.

§Refers to a set of 243 Scottish human- and bovine-associated DT104 genomes sequenced and characterized previously [103].

||Refers to a set of 290 DT104 genomes collected from various sources around the world, which were sequenced and characterized previously [14].

¶Refers to the union of Dataset 1 (U.S. bovine and human data), Dataset 2 (Scottish bovine and human data) and Dataset 3 (multi-source data); 11 of the 290 genomes in Dataset 3 (multi-source data) were isolated from cattle and humans in the USA and thus were also included in Dataset 1 (U.S. bovine and human data).

Subsequent investigation confirmed that, for all 177 *artAB-*harbouring Dataset 1 (U.S. bovine and human data) genomes, *artAB* was located within Gifsy-1 prophage regions classified as ‘intact’ via PHASTER (Tables 2 and S5, Figs S17 and S18). *gogB* was largely harboured within regions annotated via PHASTER as Gifsy-1 [126 of 180 *gogB*-harbouring Dataset 1 (U.S. bovine and human data) genomes, 70.0 %], although only 51 of these Gifsy-1 regions were annotated as intact prophages via PHASTER [28.3 % of *gogB*-harbouring Dataset 1 (U.S. bovine and human data) genomes; Tables 2 and S5, Fig. S19). Occasionally, among Dataset 1 (U.S. bovine and human data) genomes, *gogB* was detected elsewhere in the genome: three genomes harboured *gogB* within regions annotated as prophage Gifsy-2 [3 of 180 *gogB*-harbouring Dataset 1 (U.S. bovine and human data) genomes, 1.7 %; Tables 2 and S5), while *gogB* was detected outside of annotated prophage regions within the remaining 51 *gogB*-harbouring genomes [via PHASTER, 28.3 % of *gogB*-harbouring Dataset 1 (U.S. bovine and human data) genomes; Tables 2 and S5].

**Table 2.** Location of *artAB* and *gogB* in DT104 complex genomes within the four datasets queried in this study

Genes*	Dataset (no. of genomes with gene/total no. of genomes; %)	No. of genes detected*						
		Within Gifsy-1†		Within Gifsy-2†		Within *Salmonella* phage 118970_sal3†		Outside of annotated prophage regions (%)†‡
		Intact (%)‡	Incomplete (%)‡	Intact (%)‡	Incomplete (%)‡	Intact (%)‡	Incomplete (%)‡	
*artAB*								
	Dataset 1§ (177/230; 77.0%)	177 (100.0)	0 (0)	0 (0)	0 (0)	0 (0)	0 (0)	0 (0)
	Dataset 2|| (240/243; 98.8%)	21 (8.8)	0 (0)	0 (0)	0 (0)	0 (0)	0 (0)	219 (91.3)¶
	Dataset 3# (271/290; 93.4%)	263 (97.0)	0 (0)	0 (0)	0 (0)	2 (0.7)	0 (0)	6 (2.2)**
	Dataset 4†† (678/752; 90.2%)	451 (66.5)	0 (0)	0 (0)	0 (0)	2 (0.3)	0 (0)	225 (33.2)
*gogB*								
	Dataset 1§ (180/230; 78.3%)	51 (28.3)	75 (41.7)	1 (0.6)	2 (1.1)	0 (0)	0 (0)	51 (28.3)
	Dataset 2|| (240/243; 98.8%)	50 (20.8)	85 (35.4)	0 (0)	0 (0)	0 (0)	0 (0)	105 (43.8)
	Dataset 3# (271/290; 93.4%)	11 (4.1)	67 (24.7)	2 (0.7)	1 (0.4)	0 (0)	0 (0)	190 (70.1)
	Dataset 4†† (681/752; 90.6%)	112 (16.4)	223 (32.7)	3 (0.4)	3 (0.4)	0 (0)	0 (0)	340 (49.9)

*Identified using nucleotide blast (blastn; default settings, with no minimum identity or coverage threshold employed).

†Identified using the PHASTER webserver; ‘intact’ refers to prophage classified by PHASTER as ‘intact’, while ‘incomplete’ encompasses prophage classified as ‘incomplete’ or ‘questionable’.

‡Percentages in parentheses were calculated using the ‘No. of genomes with gene’ value in the ‘Dataset’ column as a denominator.

§Refers to the set of 230 U.S. human- and bovine-associated DT104 complex genomes identified and aggregated here.

||Refers to a set of 243 Scottish human- and bovine-associated DT104 genomes sequenced and characterized previously [103].

¶When 3 kb regions on either side of PHASTER prophage regions were considered, 234 Dataset 2 (Scottish bovine and human data) genomes harboured *artAB* within 3 kb of Gifsy-1 [97.5 % of 240 *artAB*-harbouring genomes in Dataset 2 (Scottish bovine and human data)]; two and one genome harboured *artAB* within 3 kb of other PHASTER prophage regions (annotated by PHASTER as Edward_GF_2_NC_026611 and PHAGE_Entero_HK630_NC_019723, respectively).

#Refers to a set of 290 DT104 genomes collected from various sources around the world, which were sequenced and characterized previously [14].

**One genome (DK_7322994_6_swine_08-08-01) had *artA* and *artB* on separate contigs, with *artB* detected within 3 kb of an intact Gifsy-1 prophage region.

††Refers to the union of Dataset 1 (U.S. bovine and human data), Dataset 2 (Scottish bovine and human data) and Dataset 3 (multi-source data); 11 of the 290 genomes in Dataset 3 (multi-source data) were isolated from cattle and humans in the USA and thus were also included in Dataset 1 (U.S. bovine and human data).

Only three genomes within Dataset 1 (U.S. bovine and human data) possessed an intact Gifsy-1 prophage via PHASTER but did not possess *artAB* [i.e. bovine-associated BOV_TYPH_Washington_2007_SRR1519881, BOV_TYPH_Minnesota_2010_SRR1089590 and BOV_TYPH_Minnesota_2008_SRR1177378, 1.7 % of Dataset 1 (U.S. bovine and human data) genomes in which an intact Gifsy-1 was detected; Fig. S20 and Tables S3 and S4]. Interestingly, all three genomes possessed *gogB* (Table S4). *gogB* was detected within an incomplete Gifsy-1 prophage region in the two genomes from Minnesota, while the genome from Washington did not harbour *gogB* within an annotated prophage region (via PHASTER; Tables 1, 2 and S5, Fig. S20).

Of the 168 bovine-associated Dataset 1 (U.S. bovine and human data) genomes, 150 (89.3 %) possessed *artAB, gogB* and Gifsy-1, while 153 (91.1 %) possessed *gogB* and Gifsy-1 (Figs 3 and S6–S8, Table 1). Interestingly, of 62 human-associated Dataset 1 (U.S. bovine and human data) genomes, only 27 (43.5 %) possessed *artAB, gogB* and Gifsy-1 (Figs 3 and S6–S8, Table 1), indicating that Gifsy-1/*artAB*/*gogB* have a negative association with human-associated DT104 complex strains from the USA (two-sided FET raw *P*<4.1×10^−12^, OR=0.094; Table 1). However, no orthologous gene clusters within the Dataset 1 (U.S. bovine and human data) pan-genome shared a significant association with bovine or human host when accounting for population structure (treeWAS FDR-corrected *P*>0.10).

Overall, 90 orthologous gene clusters within the Dataset 1 (U.S. bovine and human data) pan-genome were associated with Gifsy-1 presence or absence (via PHASTER; two-sided FET FDR-corrected *P*<0.05, Fig. S13 and Table S9). The presence and absence of 30 orthologous gene clusters shared a perfect association with Gifsy-1 presence and absence (via PHASTER; Table S9). These genes were absent from all Dataset 1 (U.S. bovine and human data) genomes that did not possess Gifsy-1 and were present in all Dataset 1 (U.S. bovine and human data) genomes that did possess Gifsy-1 (FDR-corrected *P*<0.05 and OR=∞); in addition to *gogB*, these genes included numerous phage-associated proteins (Table S9). Interestingly, genomes in which PHASTER did not detect an intact Gifsy-1 prophage region tended to possess a ColRNAI plasmid replicon (two-sided FET raw *P*<1.0×10^−27^; Figs S6–S8 and Table S3).

### An MDR DT104 complex lineage circulating among cattle and humans across the USA lost *artAB*- and *gogB*-harbouring prophage Gifsy-1 in the 1980s

To gain insight into the evolutionary relationships of *artAB*-negative U.S. DT104 complex strains, a time-scaled phylogeny was reconstructed using human- and bovine-associated U.S. DT104 complex genomes [i.e. genomes within Dataset 1 (U.S. bovine and human data); Figs 3 and S6–S8). The common ancestor of the MDR U.S. bovine- and human-associated DT104 complex genomes included in this study was predicted to have existed circa 1975 (estimated node age 1974.9, node height 95 % HPD interval [1958.1, 1986.4]; Figs 3 and S6–S8); this is consistent with observations made in previous studies [14, 115], in which DT104 was predicted to have acquired its MDR phenotype in the 1970s. The mean evolutionary rate estimated for the Dataset 1 (U.S. bovine and human data) genomes queried here was 1.75×10^−7^ substitutions per site per year (95 % HPD interval [1.38×10^−7^, 2.11×10^−7^]), which is similar to evolutionary rates estimated in previous studies of DT104 isolates from other regions of the world [14, 103] (Fig. S5, Table S8 and Supplementary Data).

Notably, over 75 % of all Dataset 1 (U.S. bovine and human data) *artAB*-negative genomes [42 of 53 Dataset 1 (U.S. bovine and human data) *artAB*-negative genomes, 79.2 %] were members of a single, well-supported clade (posterior probability=1.0, referred to hereafter as the ‘U.S. *artAB*-negative major clade’; Figs 3 and S6–S8, and Table S15). In addition to lacking *artAB*, all members of the U.S. *artAB*-negative major clade lacked Gifsy-1 and 50 additional genes, which were present in over half of all Dataset 1 (U.S. bovine and human data) genomes not included in the U.S. *artAB*-negative major clade, including *gogB*, a chitinase and many phage-associated proteins (Figs 3 and S6–S8, and Table S10).

Strains within the U.S. *artAB*-negative major clade were reportedly isolated between 1997 and 2018 (the most recent year included in this study) from at least 11 different states across the USA (for two isolates, the state in which the strain was isolated was unknown; Figs 2b and 3, and S6–S8, and Table S15). Interestingly, most strains within the U.S. *artAB*-negative major clade were isolated from humans (*n*=30 of 42 U.S. *artAB*-negative major clade strains, 71.4 %), and nearly half of all Dataset 1 (U.S. bovine and human data) genomes from human sources were members of this clade [*n*=30 of 62 Dataset 1 (U.S. bovine and human data) genomes from human sources, 48.4 %; Tables S1 and S15]. Human-associated U.S. *artAB*-negative major clade strains were reportedly isolated from six states between 1997 and 2014 (Table S15). The majority of human-associated strains were isolated in Pennsylvania (*n*=22 of 30 human-associated U.S. *artAB*-negative major clade strains, 73.3 %; Fig. 2b and Table S15); however, Pennsylvania strains were reportedly isolated over a 5 year period (i.e. from 2009 to 2014; Table S15) and showed considerable genomic diversity (Fig. 3), indicating that it is highly unlikely that all human cases have an epidemiological link (i.e. they were not sequenced as part of a single, point-source outbreak). Bovine strains within the U.S. *artAB*-negative major clade were isolated from cattle or beef products (*n*=12 of 42 U.S. *artAB*-negative major clade genomes, 28.6 %; Fig. 2b and Table S15). Much like their human-associated counterparts, bovine-associated members of the U.S. *artAB*-negative major clade were interspersed throughout the clade’s phylogeny and varied in terms of isolation date (i.e. 2004 to 2018) and geographical origin (i.e. six states; Fig. 2b and Table S15).

Based on results of ancestral state reconstruction using *artAB* presence/absence, the loss of Gifsy-1, *artAB*, *gogB* and other Gifsy-1-associated genes among members of the U.S. *artAB*-negative major clade was estimated to have occurred between 1985 and 1987 (estimated node ages 1985.0 and 1987.2, node height 95 % HPD intervals [1979.0, 1990.2] and [1981.7, 1992.1], respectively; Figs 3 and S10–S12). This predicted loss event occurred around a predicted rapid increase in the U.S. DT104 complex effective population size in the mid- to late 1980s (Figs 4 and S9). Following this predicted rapid increase in the 1980s, the U.S. DT104 complex effective population size was predicted to have increased again in the mid- to late 1990s, peaking circa 2000 (Figs 4 and S9).

### Loss of *artAB* and *gogB* within the global DT104 complex population occurs sporadically

The absence of Gifsy-1, *artAB* and/or *gogB* among DT104 complex strains was not strictly a USA phenomenon: Gifsy-1, *artAB* and *gogB* were not detected in three and 19 genomes out of (i) 243 DT104 strains isolated from cattle and humans in Scotland [referred to here as ‘Dataset 2 (Scottish bovine and human data)’] [103], and (ii) 290 DT104 strains collected from numerous sources around the world [referred to here as ‘Dataset 3 (multi-source data)’] [14], respectively [representing 1.2 % and 6.6 % of genomes in Dataset 2 (Scottish bovine and human data) and Dataset 3 (multi-source data), respectively; Figs 5, S14 and S15, Tables 1 and S3–S5). Overall, out of 752 total DT104 complex genomes queried in this study [i.e. the union of Dataset 1 (U.S. bovine and human data), Dataset 2 (Scottish bovine and human data) and Dataset 3 (multi-source data), referred to here as ‘Dataset 4 (combined global dataset)’], *artAB* could not be detected in 74 genomes [9.8 % of 752 Dataset 4 (combined global dataset) genomes; Tables 1, S15 and S16).

The Gifsy-1/*artAB*/*gogB* loss event associated with the U.S. *artAB*-negative major clade represented the single largest *artAB* loss event observed in this study [*n*=42 of 752 total Dataset 4 (combined global dataset) genomes; Figs 5, S14 and S15). However, several additional, sporadic *artAB* loss events among clades encompassing five or fewer genomes were observed (Figs 5, S14 and S15, Table S16). Overall, the 32 *artAB*-negative genomes that did not belong to the U.S. *artAB*-negative major clade were isolated from (i) a variety of sources (i.e. humans, cattle, pigs and poultry), (ii) on four continents (i.e. North America, Europe, Asia and Oceania), and (iii) between 1992 and 2015 (Table S16).

Among all 752 Dataset 4 (combined global dataset) genomes, the presence and absence of *artAB* and *gogB* was correlated with that of Gifsy-1 (two-sided FET raw *P*<2.2×10^−16^ for each, OR=2069.8 and ∞, respectively), as well as each other (two-sided FET raw *P*<2.2×10^−16^, OR=∞; Figs 5 and S14, Table 1). However, unlike the 177 *artAB*-harbouring Dataset 1 (U.S. bovine and human data) genomes queried here, *artAB* was not always detected within prophage regions annotated as Gifsy-1 in other datasets [i.e. genomes in Dataset 2 (Scottish bovine and human data) and Dataset 3 (multi-source data); Tables 2 and S5, Fig. S21). Two genomes from Dataset 3 (multi-source data) harboured *artAB* within regions annotated as *Salmonella* phage 118970_sal3 (using PHASTER’s nomenclature, ‘PHAGE_Salmon_118970_sal3_NC_031940’; Tables 2 and S5). However, despite not being annotated by PHASTER as ‘Gifsy-1’, these prophage regions shared a high degree of sequence homology with the DT104 Gifsy-1 prophage (Fig. S21).

Notably, for over 90 % of *artAB*-harbouring genomes in Dataset 2 (Scottish bovine and human data), *artAB* was identified outside of prophage regions annotated by PHASTER [*n*=219 of 240 *artAB*-harbouring genomes in Dataset 2 (Scottish bovine and human data]; Tables 2 and S5]. However, when 3 kb regions on either side of PHASTER prophage regions were considered, 234 Dataset 2 (Scottish bovine and human data) genomes harboured *artAB* within 3 kb of Gifsy-1 [97.5 % of 240 *artAB*-harbouring genomes in Dataset 2 (Scottish bovine and human data)]. Two and one genome harboured *artAB* within 3 kb of other PHASTER prophage regions (annotated by PHASTER as Edward_GF_2_NC_026611 and PHAGE_Entero_HK630_NC_019723, respectively). For Dataset 3 (multi-source data), six *artAB*-positive genomes did not harbour *artAB* within PHASTER prophage regions (Tables 2 and S5). For one of these genomes (DK_7322994_6_swine_08-08-01), *artA* and *artB* were detected on separate contigs, with *artB* present within 3 kb of a PHASTER prophage region annotated as intact Gifsy-1; for the remaining five genomes, *artAB* was not located within a 5 kb region upstream or downstream of any PHASTER prophages (Tables 2 and S5).

### *In vitro* response of U.S. DT104 complex strains to human- and bovine-associated gastrointestinal stress factors is not correlated with the presence of *artAB*- and *gogB*-harbouring Gifsy-1

The (i) loss of Gifsy-1/*artAB*/*gogB* associated with the U.S. *artAB*-negative major clade around a predicted rapid increase in the U.S. DT104 complex effective population size, plus (ii) the over-representation of human strains in the U.S. *artAB*-negative major clade led us to hypothesize that ArtAB and/or GogB production (or some other genomic element harboured on Gifsy-1) may influence the dynamics of DT104 complex strains in the digestive tracts of human and bovine hosts. Thus, we used phenotypic assays that simulated human and/or bovine digestion-associated stress conditions to compare the phenotypes of Gifsy-1/*artAB*/*gogB*-negative members of the U.S. *artAB*-negative major clade to those of the most closely related, Gifsy-1/*artAB*/*gogB*-positive U.S. DT104 complex strains available (Table S11).

**Fig. 6.**
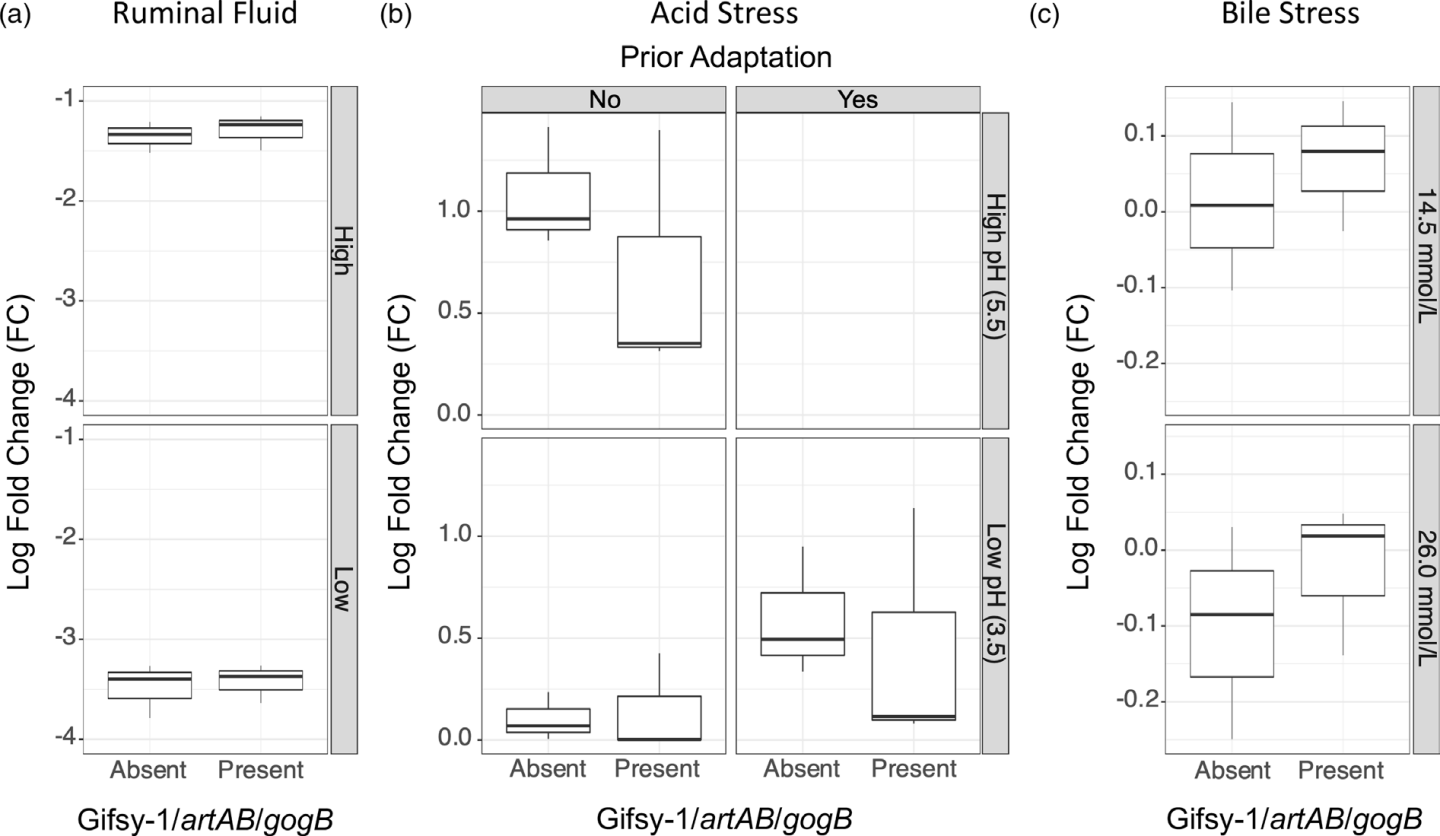
Response of DT104 complex isolates (*n*=6) to environmental stress factors within the context of Gifsy-1/*artAB*/*gogB* presence and absence. Base-10 logarithmic fold change (FC) was calculated as follows: FC=log c.f.u. g^−1^ at the start of the experiments – log c.f.u. g^–1^ after the stress assay. (**a**) Log FC of DT104 inoculated into ruminal fluid at high (10^8^ c.f.u. ml^−1^; ‘High’) or low (10^5^ c.f.u. ml^−1^; ‘Low’) bacterial numbers. (**b**) Log FC of DT104 isolates exposed to inorganic acid stress (pH 3.5) with or without a prior adaption step with an intermediate pH (pH 5.5). (**c**) Log FC of DT104 isolates after exposure to bile salt at two concentrations. Supplementary Data have been uploaded to figshare: https://doi.org/10.6084/m9.figshare.22194385.v1.

As the first three compartments of the bovine digestive tract differ massively from that of the human gut, the phenotype of Gifsy-1/*artAB*/*gogB-*positive and -negative strains was investigated in fresh bovine RF obtained from a donor cow (Table S12). DT104 complex concentrations were reduced by 3.4 log c.f.u. (sd=0.2) when inoculated into RF at a final concentration of 10^5^ c.f.u. ml^−1^, whereas DT104 complex numbers were reduced by 1.3 log c.f.u. (sd=0.2) when inoculated at a final concentration of 10^8^ c.f.u. ml^−1^ (Fig. 6). While the inoculation density did significantly affect survival (ANOVA raw *P*<0.001), the phenotype in RF was not associated with the presence or absence of Gifsy-1/*artAB*/*gogB* (ANOVA raw *P*>0.05; Fig. 6).

The presence of Gifsy-1/*artAB*/*gogB* also did not significantly influence acid stress survival at pH 3.5 (ANOVA raw *P*>0.05; Fig. 6 and Table S13). While prior adaptation at an intermediate pH of 5.5 significantly increased survival at pH 3.5 as expected (ANOVA raw *P*<0.01), there was no significant difference in acid adaptation between Gifsy-1/*artAB*/*gogB*-positive and -negative strains (ANOVA raw *P*>0.05; Fig. 6). Both groups showed a concentration-dependent reduction in growth/survival at the two tested bile concentrations of 0.6 % and 1.1 % (ANOVA raw *P*=0.01 for the difference in fold change at the two concentrations), but there was no Gifsy-1/*artAB*/*gogB*-dependent phenotype in the response of DT104 complex strains to bile stress (ANOVA raw *P*>0.05; Fig. 6 and Table S14).

## Discussion

### Members of the DT104 complex largely harbour *artAB* on a Gifsy-1-like prophage

Bacterial ADP-ribosylating toxins play important roles in the virulence of numerous pathogens [20, 116]. While the illness caused by *S. enterica* is not considered to be a toxin-mediated disease in the classical sense (e.g. as is the case for *Clostridium botulinum* or *Vibrio cholerae *) [20], some *Salmonella* lineages are capable of producing ADP-ribosylating toxins, allowing them to alter host immune responses and promote pathogenesis [18, 20, 117–119]. ArtAB is one such toxin with a variable presence among *Salmonella* lineages: genes encoding ArtAB have been detected in at least 88 different serotypes and are correlated with the presence of typhoid toxin genes [25], although in DT104 this is not the case [18, 26]. Additionally, in the majority of these serotypes, *artA* is predicted to be a pseudogene and the selective advantage of maintaining *artB* appears to be related to its use as an alternative binding subunit for the typhoid toxin [20, 120].

A previous study of ArtAB-producing DT104 strains [19] found that ArtAB production among DT104 appears to be the norm rather than the exception, as 237 of 243 strains (97.5 %) in the study were *artAB*-positive [19]. We observed similar findings here, as *artAB* was detected in 678 of 752 DT104 complex genomes (90.2 %; Tables 1 and S4). Among USA human- and bovine-associated DT104 complex genomes [i.e. Dataset 1 (U.S. bovine and human data)], *artAB* was exclusively harboured on prophage regions identified by PHASTER as intact Gifsy-1 [177 of 177 *artAB*-harbouring Dataset 1 (U.S. bovine and human data) genomes, 100 %; Table 2). It is important to note that Gifsy-1 prophage regions – as defined via PHASTER – can display a significant degree of genetic heterogeneity [29]. Furthermore, Gifsy-1 has been shown to share homology with Gifsy-2 [121, 122], a result observed here (Figs 1a, S1 and S17–S20). Thus, defining a ‘ground-truth’ Gifsy-1 prophage in the genomic era may not necessarily be straightforward, as both Gifsy-1 and Gifsy-2 were originally identified in 1997 using Southern blotting [121]. Considering PHASTER results (Tables 2 and S5), our own sequence homology observations (Figs S17–S20), and the fact that previous studies of DT104 have reportedly identified *artAB* within Gifsy-1 [11, 20], we are confident that the *artAB*-harbouring prophage identified in Dataset 1 (U.S. bovine and human data) can be safely referred to as Gifsy-1.

Among genomes from other sources and/or world regions [i.e. Dataset 2 (Scottish bovine and human data) and Dataset 3 (multi-source data)], *artAB* was not always detected within the bounds of prophage regions annotated as Gifsy-1 (Table 2). Two *artAB*-harbouring prophages that were not annotated as ‘Gifsy-1’ by PHASTER, for example, shared a high degree of sequence homology with DT104 Gifsy-1 (Fig. S21), further highlighting the challenges associated with genomic differentiation of homology-sharing prophages defined in the pre-genomics era. However, most notably, *artAB* was frequently detected outside of annotated prophage regions in Dataset 2 (Scottish bovine and human data; Table 2). When 3 kb regions upstream and downstream of PHASTER prophage regions were considered, nearly all *artAB*-harbouring Dataset 2 (Scottish bovine and human data) genomes possessed *artAB* within 3 kb of Gifsy-1. While it is possible that *artAB* is indeed harboured outside of Gifsy-1 in these genomes, we suspect this is an artefact of prophage boundary prediction. Precise prediction of prophage boundaries is challenging [123], and additional factors (e.g. assembly fragmentation in prophage regions) can further affect boundary accuracy [46]. We thus encourage readers to interpret these results with caution. Future long-read sequencing efforts will thus probably provide much-needed insight into the prophage repertoire of the DT104 complex.

### A DT104 complex lineage isolated across multiple USA states for over 20 years lost its ability to produce the ArtAB toxin and anti-inflammatory effector GogB

Here, we observed that *artAB* loss events appear sporadically throughout the DT104 complex phylogeny (Figs 3 and 5). Among USA human- and bovine-associated DT104 complex genomes [i.e. Dataset 1 (U.S. bovine and human data)], these loss events usually coincided with Gifsy-1 loss, although not exclusively (i.e. three strains did not possess *artAB*, but possessed Gifsy-1; Figs 3 and S20). As mentioned above, *in silico* prophage detection and differentiation is challenging, and it is possible that HGT within a Gifsy-1-like prophage led to the loss of *artAB*, rather than the complete excision of Gifsy-1 in its entirety. In this scenario, it is plausible that partial Gifsy-1 remnants within these genomes would not be classified as Gifsy-1 prophage elements, or they would not be detected by *in silico* prophage detection methods. Regardless, we have identified 30 prophage-associated genes, which were detected in all Gifsy-1-harbouring genomes and absent from all Gifsy-1-negative genomes in Dataset 1 (U.S. bovine and human data), indicating that numerous prophage-associated genes were lost along with *artAB* and *gogB* (Table S9).

Most notably, we observed a MDR DT104 complex clade circulating among cattle and humans across 11 USA states, which lost Gifsy-1, concomitant with the ability to produce ArtAB and GogB (i.e. the U.S. *artAB*-negative major clade; Fig. 3). Considering (i) genomic diversity observed within the U.S. *artAB*-negative major clade, along with the fact that (ii) U.S. *artAB*-negative major clade members have been isolated from multiple states and sources for over 20 years, it is nearly impossible that U.S. *artAB*-negative major clade genomes are the result of repeated sequencing of identical or nearly identical strains (e.g. as would be the case in a point-source outbreak scenario; Table S15). When U.S. *artAB*-negative major clade genomes were compared to DT104 complex genomes collected from a variety of isolation sources around the world (Fig. 5), we did not identify genomes from any country other than the USA within this clade, nor did we identify genomes from any isolation source other than cattle and humans (Fig. 5 and Table S15). Furthermore, we observed a high proportion of human-associated strains within the U.S. *artAB*-negative major clade relative to bovine-associated strains (Fig. 3 and Table S15). The (i) limited host range, (ii) limited geographical range and (iii) high human-to-bovine ratio observed for the U.S. *artAB*-negative major clade in this study is likely to be an artefact of sampling and/or sequencing (e.g. due to the large number of bovine-associated genomes included in this study relative to other animal hosts, due to this study’s focus on the DT104 complex in the USA, due to geographical and/or host biases in allocation of *Salmonella* sequencing resources). Future studies querying more DT104 complex strains from (i) non-human and non-bovine sources (e.g. other animal hosts, foods, environmental sources) and (ii) countries other than the USA and the UK will probably reveal a greater isolation source and geographical range for this clade, respectively.

However, it is important to note that the U.S. *artAB*-negative major clade contained nearly half of all DT104 complex strains isolated from humans in the USA (*n*=30 of 62 U.S. DT104 complex genomes from human sources, 48.4 %; Tables S1 and S15). This is notable, as our study included all human-associated U.S. DT104 complex genomes with metadata available in Enterobase at the time. It is certainly likely that there were U.S. DT104 complex genomes from human sources, which were not included in our study (e.g. due to missing publicly available metadata), and it is possible that there may be biases in terms of metadata reporting (e.g. some laboratories may routinely provide detailed, publicly available metadata for genomes that they sequence, while other laboratories may never or rarely provide metadata). Thus, in order to gain further insight into potential U.S. *artAB*-negative major clade host associations (or the lack thereof), it is essential that isolation source metadata are made publicly available in addition to whole genome sequencing data.

The U.S. *artAB*-negative major clade was predicted to have lost Gifsy-1/*artAB*/*gogB* circa 1985–1987, around a predicted rapid increase in the U.S. DT104 complex effective population size, which occurred in the mid- to late 1980s (Fig. 4). Our results are consistent with a previous study of DT104 from multiple world regions, which also identified periods of dramatic effective population size growth in the 1980s and 1990s [14]. This rapid increase in effective population size is notable, as it coincides with the global MDR DT104 epidemic, which occurred among humans and animals throughout the 1990s [14, 15, 103]. However, it is essential to note that our data do not imply that *artAB*, *gogB* or Gifsy-1 loss played a role in the emergence and subsequent global spread of DT104; any potential association between the virulence and/or fitness of MDR DT104 and Gifsy-1/*artAB*/*gogB* loss among DT104 complex genomes is merely speculative at this point. While previous studies of DT104 have shown that prophage excision and *artAB* loss occur in response to DNA damage and other stressors [18, 20], future studies are needed to better understand the roles that Gifsy-1, *artAB* and *gogB* play in DT104 evolution.

### Members of the U.S. *artAB*-negative major clade do not have a phenotypic advantage relative to other U.S. DT104 complex strains when exposed to ruminal fluid-, acid- and bile-associated stressors *in vitro*

*S. enterica* encounters numerous stressors within the gastrointestinal tracts of humans and animals, including (but not limited to) low pH, low oxygen, exposure to bile and the host immune system [124–126]. Furthermore, the gastrointestinal environment that *S. enterica* encounters can differ between hosts; for example, the first three compartments of the bovine digestive tract differ massively from those of the human gut, as they essentially serve as massive microbial fermentation chambers [127]. Here, we evaluated the survival of DT104 complex strains when exposed to three stressors encountered in the human and/or bovine gastrointestinal tracts: (i) RF (bovine rumen), (ii) low pH (bovine abomasum and human stomach) and (iii) exposure to bile (bovine and human duodenum); we discuss each step in detail below.

In the bovine digestion process, the RF, including the complex community of ruminal microbiota [128], presents an early line of defence against potential pathogens, such as *Salmonella* spp. In RF, the kill rate of DT104 complex strains was dependent on the inoculation density. The high inoculation rate (10^8^ c.f.u. ml^−1^) was chosen to test the ability of the ruminal microbiota to efficiently kill or impede *Salmonella *. The lower inoculation rate of 10^5^ c.f.u. ml^−1^ was chosen for its dynamic range to measure either growth or decrease of *Salmonella* concentration. An interaction of the complex ruminal microbiota with the inoculated *Salmonella* is conceivable in two ways: either the microbiota exhibit strategies to produce antimicrobial compounds against *Salmonella* species [129, 130], or through competition for nutrients, such as iron [131]. The fact that the ruminal microbiota was less effective at killing DT104 complex strains at the high inoculation rate suggests that their defence mechanisms against DT104 complex strains are limited and/or the system started to be overrun by the high numbers of the DT104 complex strain.

Gastric acids in the stomach (or abomasum) are the next line of host defence, which *Salmonella* must overcome during gastrointestinal passage [132]. A pH of 3.5 was selected based on the following considerations: the human gastric pH varies from pH <2 in a fasted state to pH >6 during meals, returning to a low pH within hours postprandially [133, 134]. Intracellular pathogens such as *Salmonella* spp. have adapted to survive low pH intracellularly in the phagosomes of phagocytes (e.g. pH 4–6) [135, 136] and express adaptive acid tolerance that allows them to tolerate pH of 2–3 [137]. Therefore, the chosen pH of 3.5 reflects a relevant physiological state of the human stomach and represents a sublethal stress to *Salmonella* spp. Our experiments confirmed that acid adaptation with HCl at pH 5.5 led to much higher survival rates at pH 3.5. Well-known mechanisms such as decreased membrane conductivity for H^+^, increased proton extrusion or changes in the cell envelope composition [137–139] could be responsible for this.

Upon leaving the stomach, enteric pathogens are confronted with bile. Bile salts show antimicrobial activity by dissolving membrane lipids and by dissociating integral membrane proteins [140], and lead to general cell damage by misfolding and denaturation of proteins [141, 142] and DNA damage [143, 144]. *S. enterica* is able to survive duodenal bile salt concentrations through DNA repair mechanisms [144], multiple changes in gene expression [145] and increased production of anti-oxidative enzymes [146]. Here, selected DT104 complex strains were able to survive at both tested bile salt concentrations (14.5 and 26.0 mmol l^–1^); however, no significant differences were observed between strains that harboured Gifsy-1/*artAB*/*gogB* and those that did not (Fig. 6).

In summary, the *in vitro* stress assays performed in this study aimed to mimic the stressors that DT104 complex strains encounter in the gastrointestinal tracts of humans and ruminants. Given the over-representation of human-associated Gifsy-1/*artAB*/*gogB*-negative strains observed here, one may be tempted to speculate that Gifsy-1, *artAB* and/or *gogB* absence may confer members of the U.S. *artAB*-negative major clade with a competitive advantage in the human host gastrointestinal tract; however, no Gifsy-1/*artAB*/*gogB*-dependent phenotype was observed in DT104 complex strains under the tested conditions (Fig. 6). Furthermore, as mentioned above, the overrepresentation of human strains in this clade could merely be an artefact of sampling/sequencing. Thus, it may be possible that Gifsy-1/*artAB*/*gogB* absence may confer some advantage(s) to U.S. *artAB*-negative major clade strains in hosts underrepresented in this study, or in environmental conditions, which were not tested in this study, including those outside of the host (e.g. high osmotic pressure and competitive microbiota in manure or wastewater, food safety measures such as disinfectants, antimicrobials and food processing) [125]. However, at present, this is merely speculation; future studies are needed to evaluate whether Gifsy-1/*artAB*/*gogB* loss among members of the U.S. *artAB*-negative major clade is merely coincidental or indicative of some evolutionarily advantageous phenotype.

### Future research is needed to understand the roles that Gifsy-1, ArtAB and GogB play in DT104 virulence

The results presented here indicate that prophage-mediated ArtAB production within the DT104 complex can undergo temporal changes. Most notably, we identified the U.S. *artAB*-negative major clade, which lost the ability to produce ArtAB and GogB, probably due to a Gifsy-1 loss event (Fig. 3). However, the ecological and/or evolutionary significance of this loss-of-function event remain unclear. Although phenotypic assessments have demonstrated a role for DT104-encoded ArtAB in both cell culture and a mouse model [19], the true benefit of this toxin in the context of human and bovine salmonellosis has not been investigated. It has been previously shown that reactive oxygen species (ROS) induce production of ArtAB [24], which may suggest that *artAB* is expressed in response to immune cell-derived ROS. Furthermore, as treatment with ArtA increases intracellular levels of cAMP in macrophage-like cells [19], ArtAB may play a role in delaying *Salmonella* clearance by altering the activity of host immune cells [20]. Hence, future studies, including in tissue culture and animal models, will be needed to determine whether *artAB* presence or absence confers a selective advantage among human- and animal-associated DT104.

## Supplementary Data

Supplementary material 1

Supplementary material 2

Supplementary material 3
