# Supplementary material for: A multidrug-resistant Salmonella enterica Typhimurium DT104 complex lineage circulating among humans and cattle in the USA lost the ability to produce pertussis-like toxin ArtAB
Source: Microb Genom. 2023 Jul 4;9(7):mgen001050. doi: 10.1099/mgen.0.001050 (PMC10438809; doi:10.1099/mgen.0.001050)
Supplement: Supplementary material 1 [file mgen-9-1050-s001.pdf]

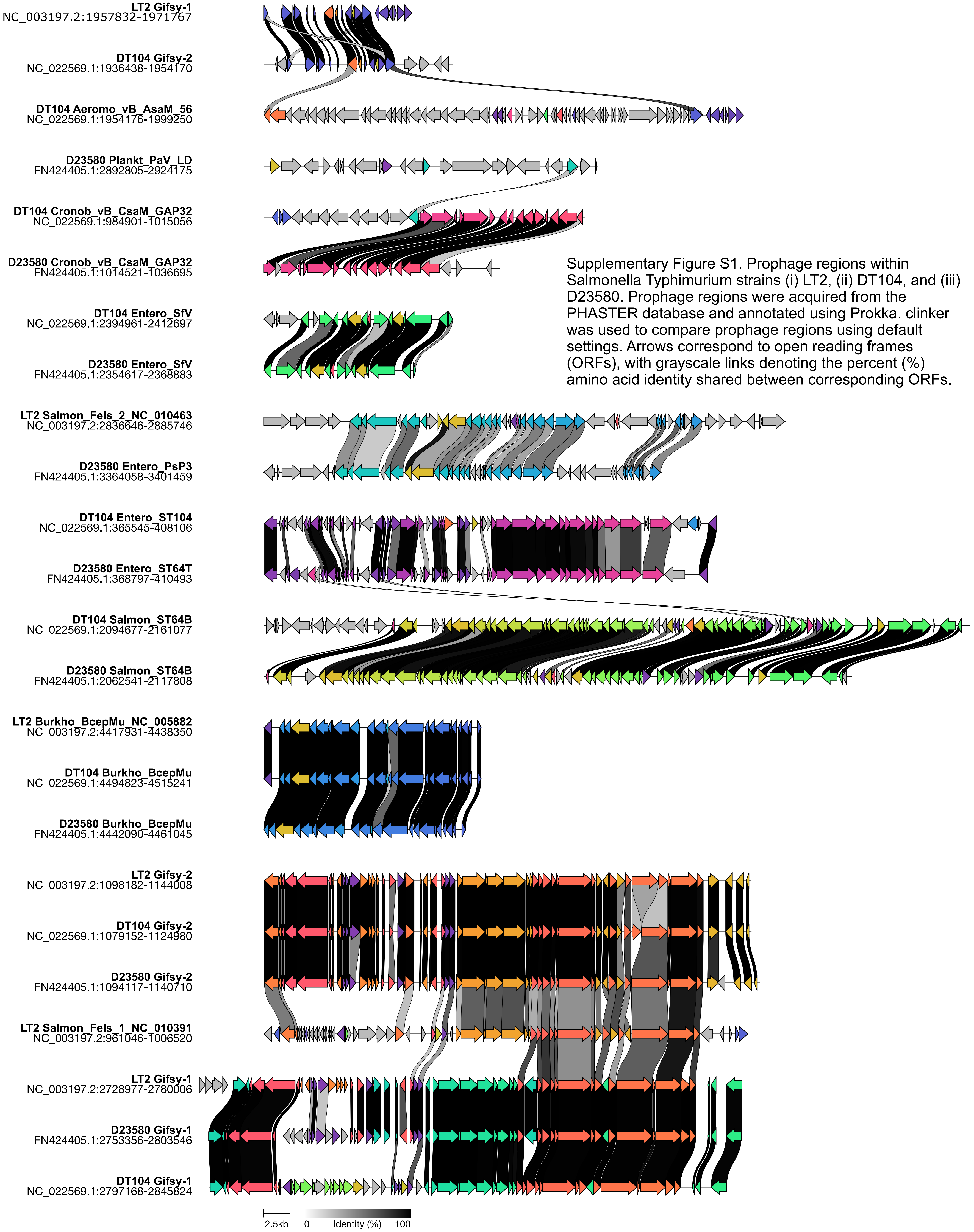

Supplementary Figure S1. Prophage regions within Salmonella Typhimurium strains (i) LT2, (ii) DT104, and (iii) D23580. Prophage regions were acquired from the PHASTER database and annotated using Prokka. clinker was used to compare prophage regions using default settings. Arrows correspond to open reading frames (ORFs), with grayscale links denoting the percent (%) amino acid identity shared between corresponding ORFs.

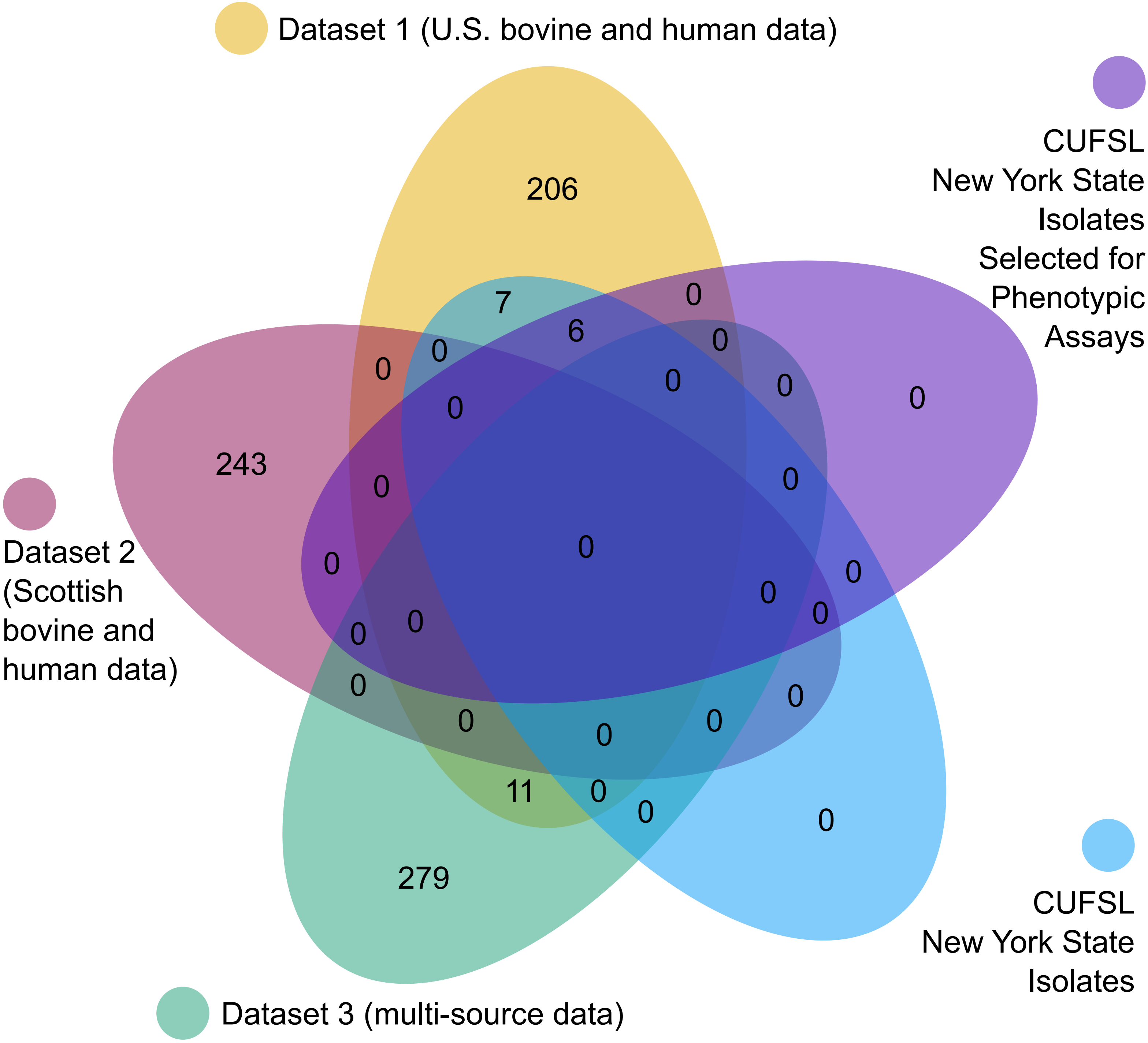

Supplementary Figure S2. Venn diagram showcasing the relationship between DT104 complex datasets used in this study. Numbers denote the number of genomes within a given dataset or subset of a dataset. For a flow chart with detailed descriptions of the datasets used in this study, see Supplementary Figure S3. CUFSL, Cornell University Food Safety Laboratory culture collection.

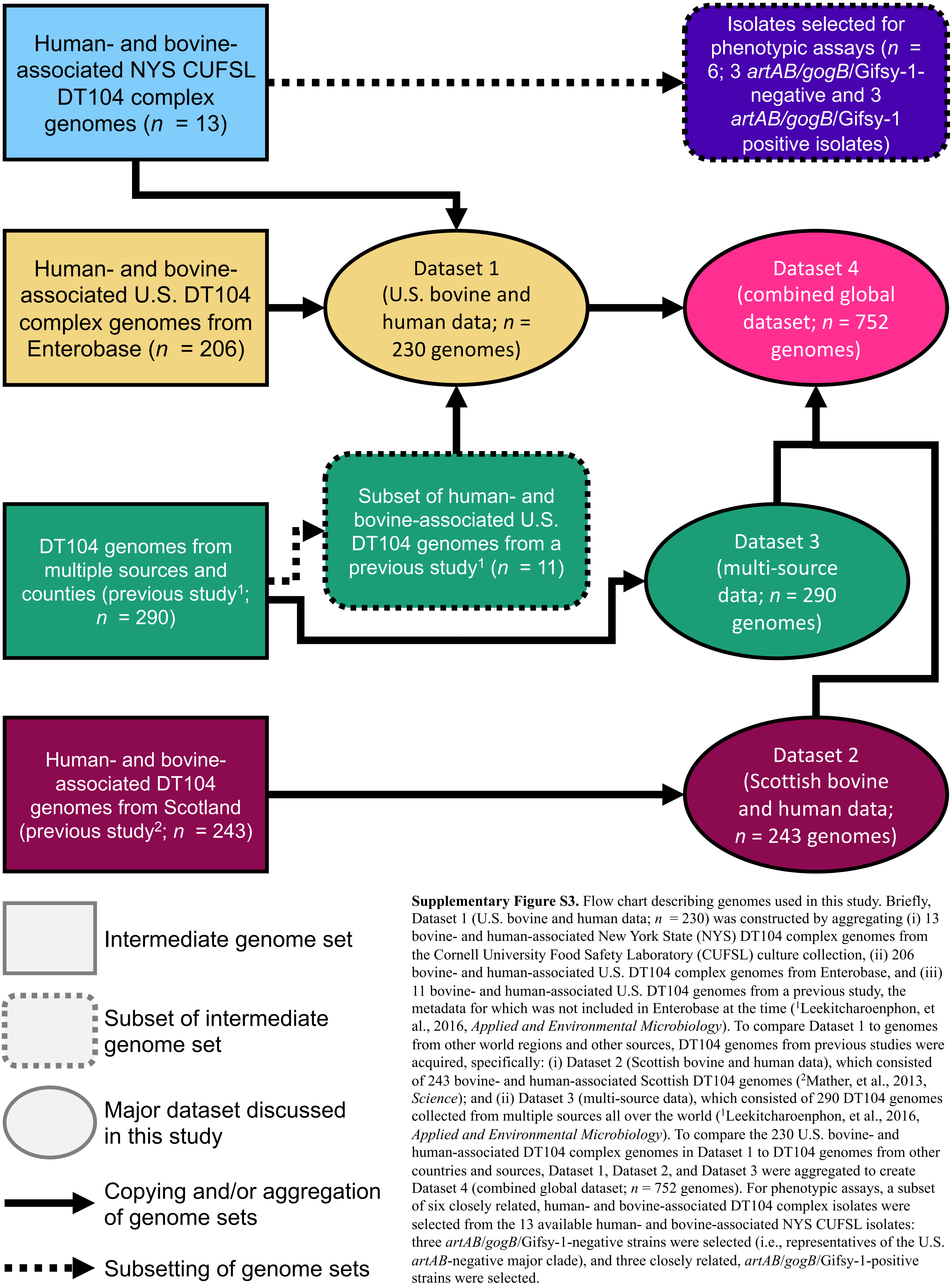

Supplementary Figure S3. Flow chart describing genomes used in this study. Briefly, Dataset 1 (U.S. bovine and human data; *n* = 230) was constructed by aggregating (i) 13 bovine- and human-associated New York State (NYS) DT104 complex genomes from the Cornell University Food Safety Laboratory (CUFSL) culture collection, (ii) 206 bovine- and human-associated U.S. DT104 complex genomes from Enterobase, and (iii) 11 bovine- and human-associated U.S. DT104 genomes from a previous study, the metadata for which was not included in Enterobase at the time ([1]Leekitcharoenphon, et al., 2016, *Applied and Environmental Microbiology*). To compare Dataset 1 to genomes from other world regions and other sources, DT104 genomes from previous studies were acquired, specifically: (i) Dataset 2 (Scottish bovine and human data), which consisted of 243 bovine- and human-associated Scottish DT104 genomes ([2]Mather, et al., 2013, *Science*); and (ii) Dataset 3 (multi-source data), which consisted of 290 DT104 genomes collected from multiple sources all over the world ([1]Leekitcharoenphon, et al., 2016, *Applied and Environmental Microbiology*). To compare the 230 U.S. bovine- and human-associated DT104 complex genomes in Dataset 1 to DT104 genomes from other countries and sources, Dataset 1, Dataset 2, and Dataset 3 were aggregated to create Dataset 4 (combined global dataset; *n* = 752 genomes). For phenotypic assays, a subset of six closely related, human- and bovine-associated DT104 complex isolates were selected from the 13 available human- and bovine-associated NYS CUFSL isolates: three *artAB*/*gogB*/Gifsy-1-negative strains were selected (i.e., representatives of the U.S. *artAB*-negative major clade), and three closely related, *artAB*/*gogB*/Gifsy-1-positive strains were selected.

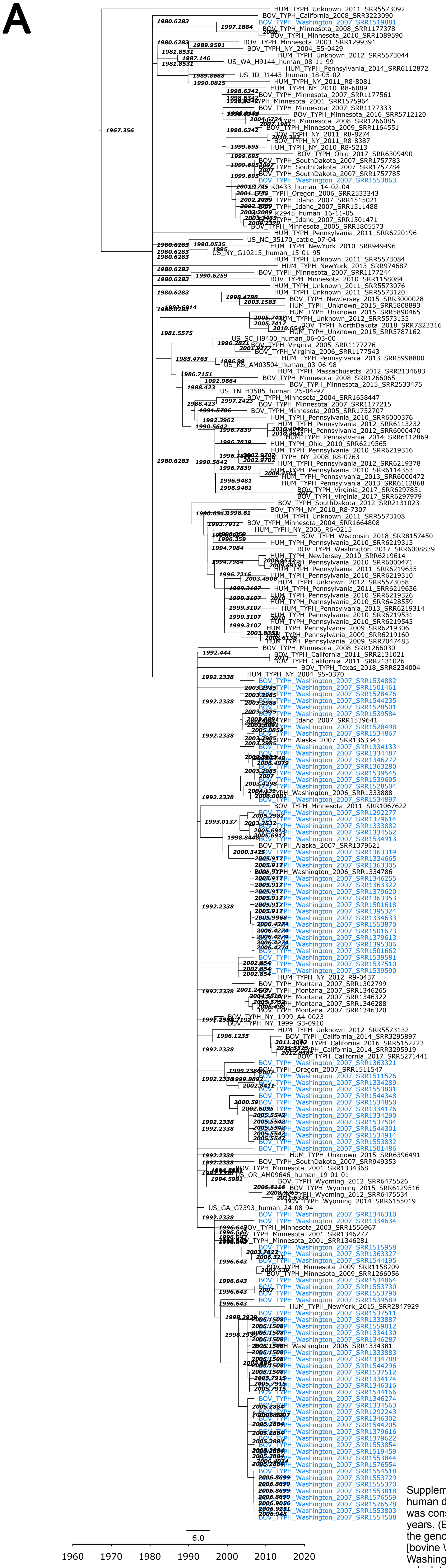

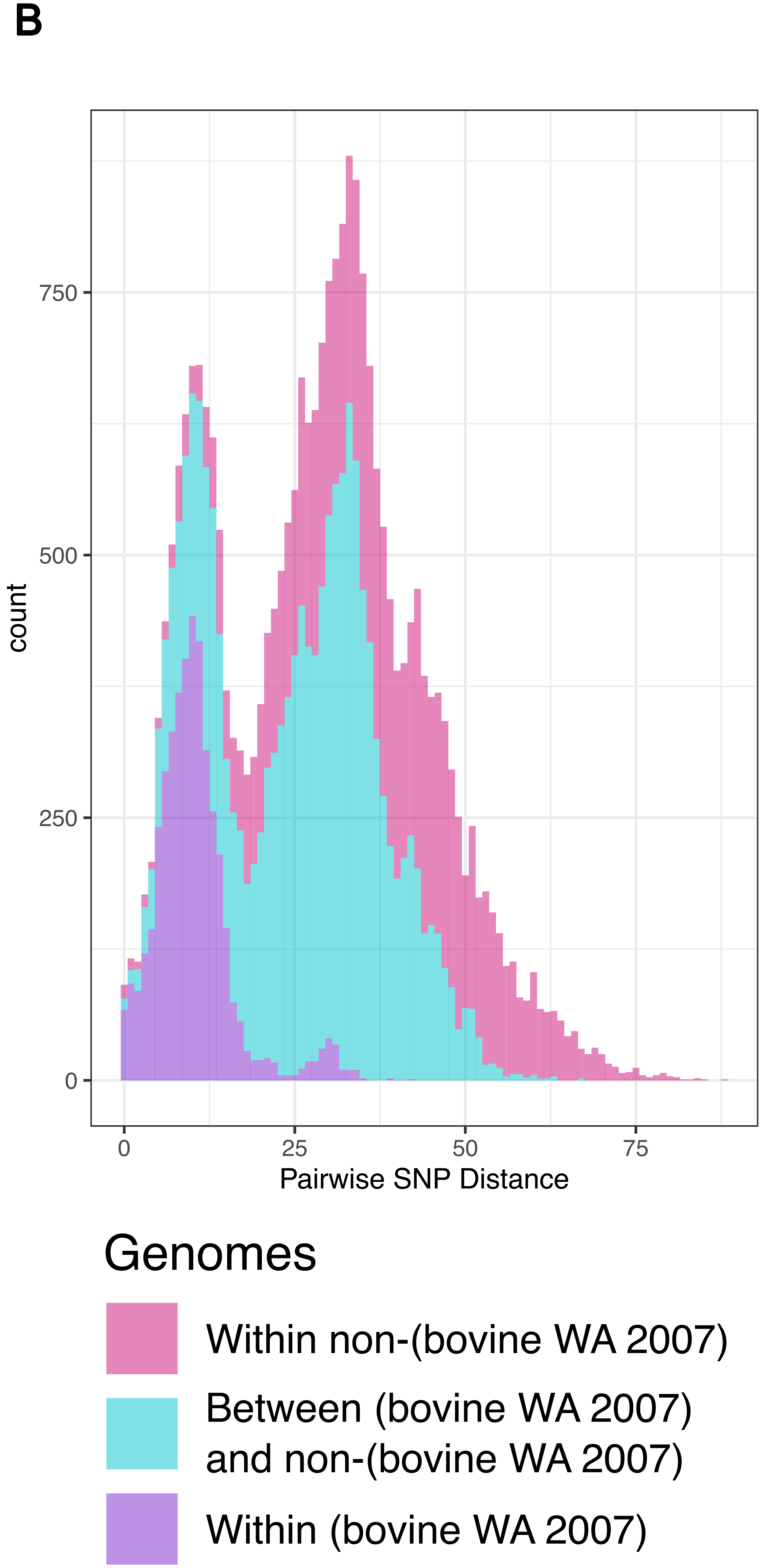

Supplementary Figure S4. (A) Time-scaled maximum likelihood (ML) phylogeny constructed using all 230 DT104 complex genomes in Dataset 1 (U.S. bovine and human data). Blue tip labels denote strains reportedly isolated from cattle in Washington State in 2007. Node labels correspond to node ages (year). The phylogeny was constructed using IQ-TREE, using core SNPs identified via Snippy as input. LSD2 was used to root and time-scale the phylogeny. Branch lengths are reported in years. (B) Histogram of pairwise SNP distances calculated between all bovine-associated U.S. DT104 complex genomes isolated in Washington State in 2007 (i.e., the genomes denoted by blue tip labels in panel A). Colored shading denotes distances calculated between (i) two 2007 bovine Washington State genomes (Within [bovine WA 2007]), (ii) two genomes not isolated in 2007 from bovine sources in Washington State (Within non-[bovine WA 2007]), and (iii) one 2007 bovine Washington State genome and one genome that was not a member of this set (Between [bovine WA 2007] and non-[bovine WA 2007]). Pairwise distances were calculated using the “dist.gene” function in the ape version 5.6-2 R package. The histogram was plotted using ggplot2.

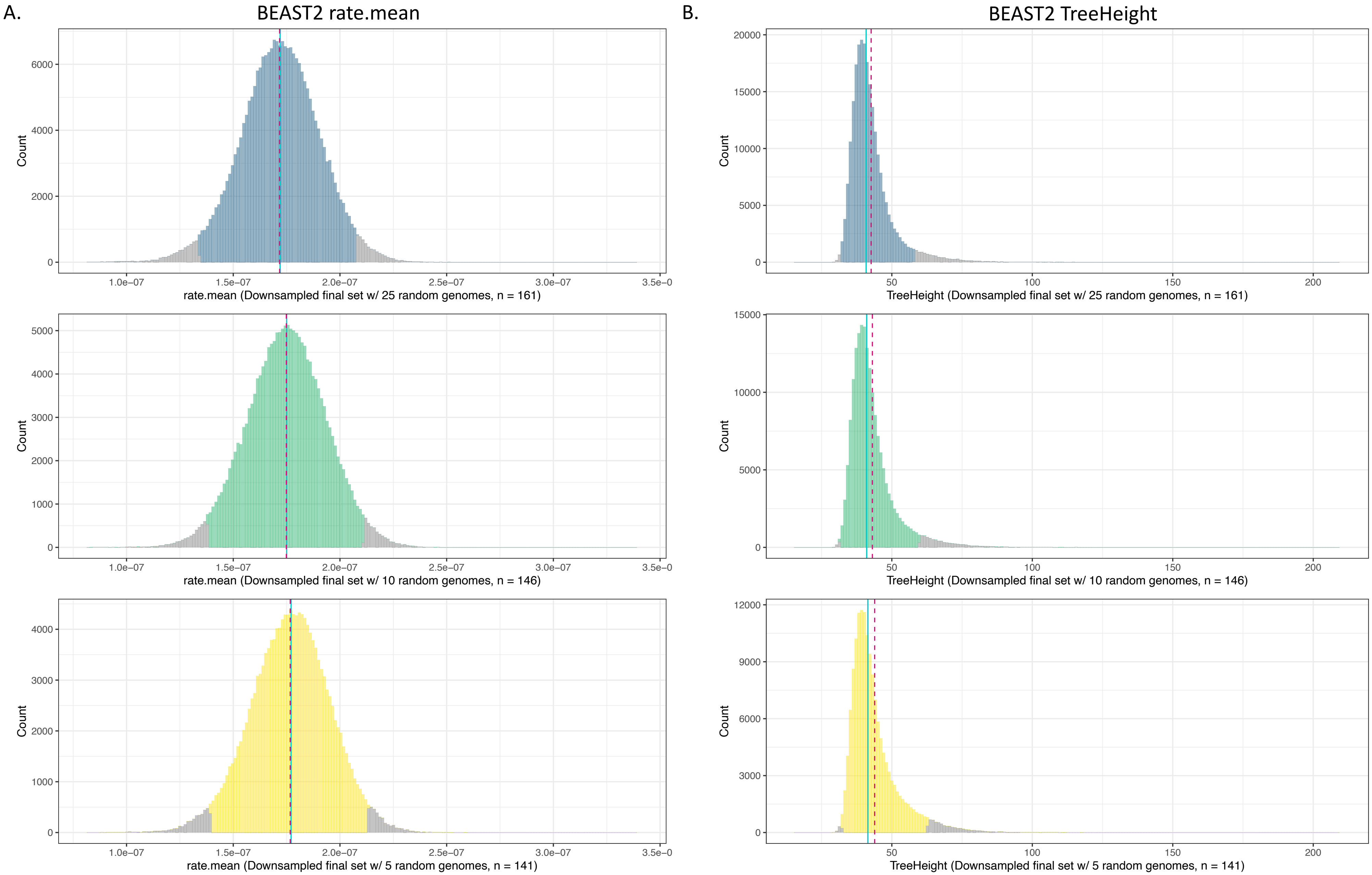

Supplementary Figure S5. Histograms of BEAST2 parameter estimates for the (A) rate.mean and (B) TreeHeight parameters for three downsampled Dataset 1 (U.S. bovine and human data) genome sets. The three genome sets were constructed by randomly selecting (i) 25, (ii) 10, and (iii) 5 bovine DT104 complex genomes collected in Washington State in 2007 from the complete set of 230 Dataset 1 (U.S. bovine and human data) genomes (n = 161, 146, and 141 total genomes in each downsampled genome set, respectively).

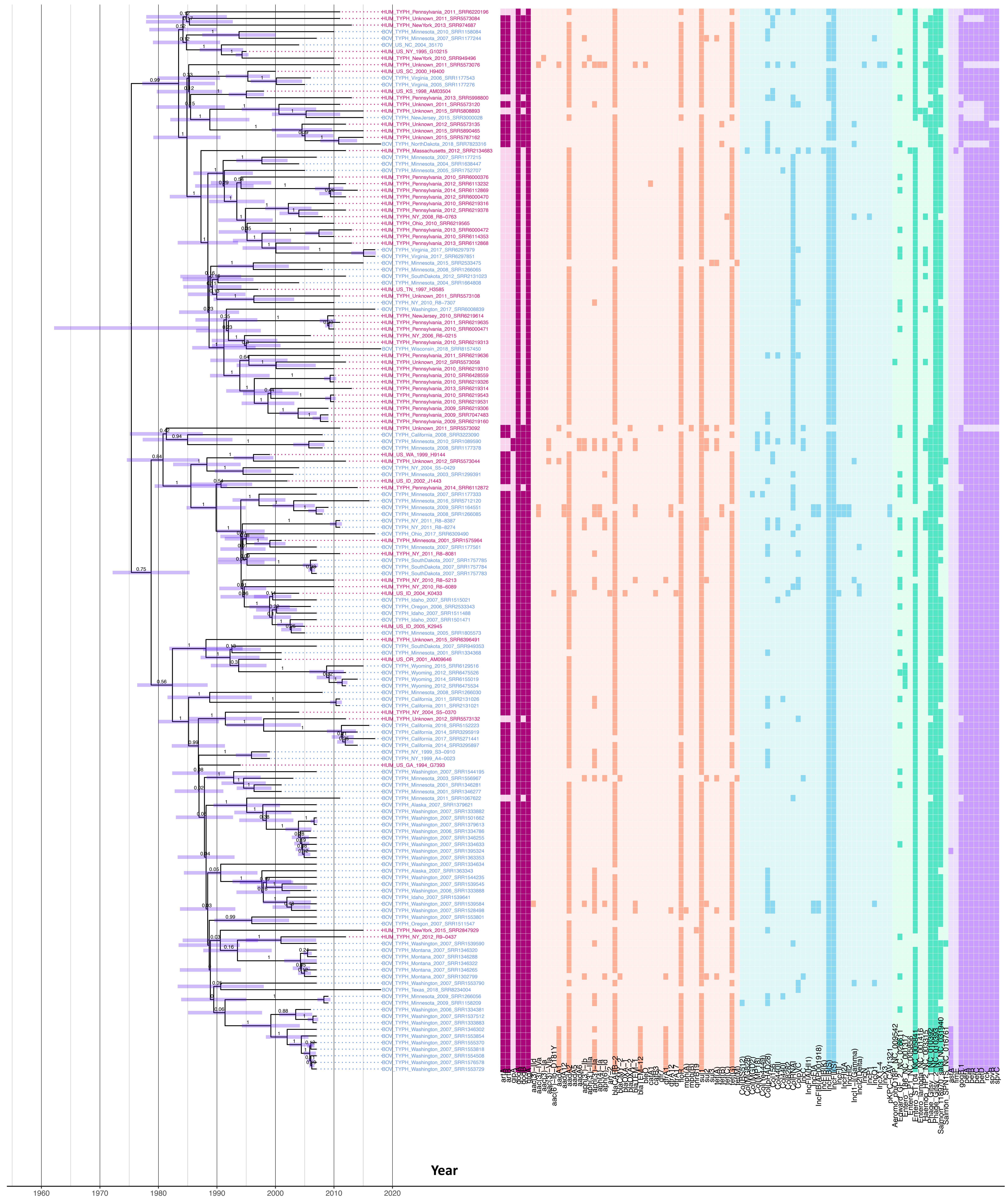

Supplementary Figure S6. Bayesian time-scaled phylogeny constructed using 161 human- and bovine-associated DT104 complex genomes collected in the United States (U.S.; i.e., the genome set downsampled from Dataset 1 [U.S. bovine and human data], using 25 randomly selected DT104 complex genomes collected from cattle in Washington State in 2007). Tip label colors denote the isolation source reported for each genome (human or bovine in pink and blue, respectively). The heatmap to the right of the phylogeny denotes the presence and absence of: (i) selected virulence factors (dark and light pink, respectively; selected virulence factors were detected using nucleotide BLAST and were considered present using a minimum coverage threshold of 40%); (ii) antimicrobial resistance (AMR) genes (dark and light orange, respectively; detected using ABRicate and the NCBI AMR database); (iii) plasmid replicons (dark and light blue, respectively; detected using ABRicate and the PlasmidFinder database); (iv) intact prophage (dark and light green, respectively; identified and classified as "intact" via PHASTER); (v) variably present Virulence Factor Database (VFDB) virulence factors (dark and light purple, respectively; detected using ABRicate and VFDB, with virulence factors detected in all 161 genomes omitted for readability). All analyses that relied on ABRicate employed minimum nucleotide identity and coverage thresholds of 75 and 50%, respectively. The phylogeny was constructed and rooted using BEAST2. Time in years is plotted along the X-axis, while branch labels correspond to posterior probabilities of branch support. Transparent purple node bars denote node height 95% highest posterior density (HPD) intervals.

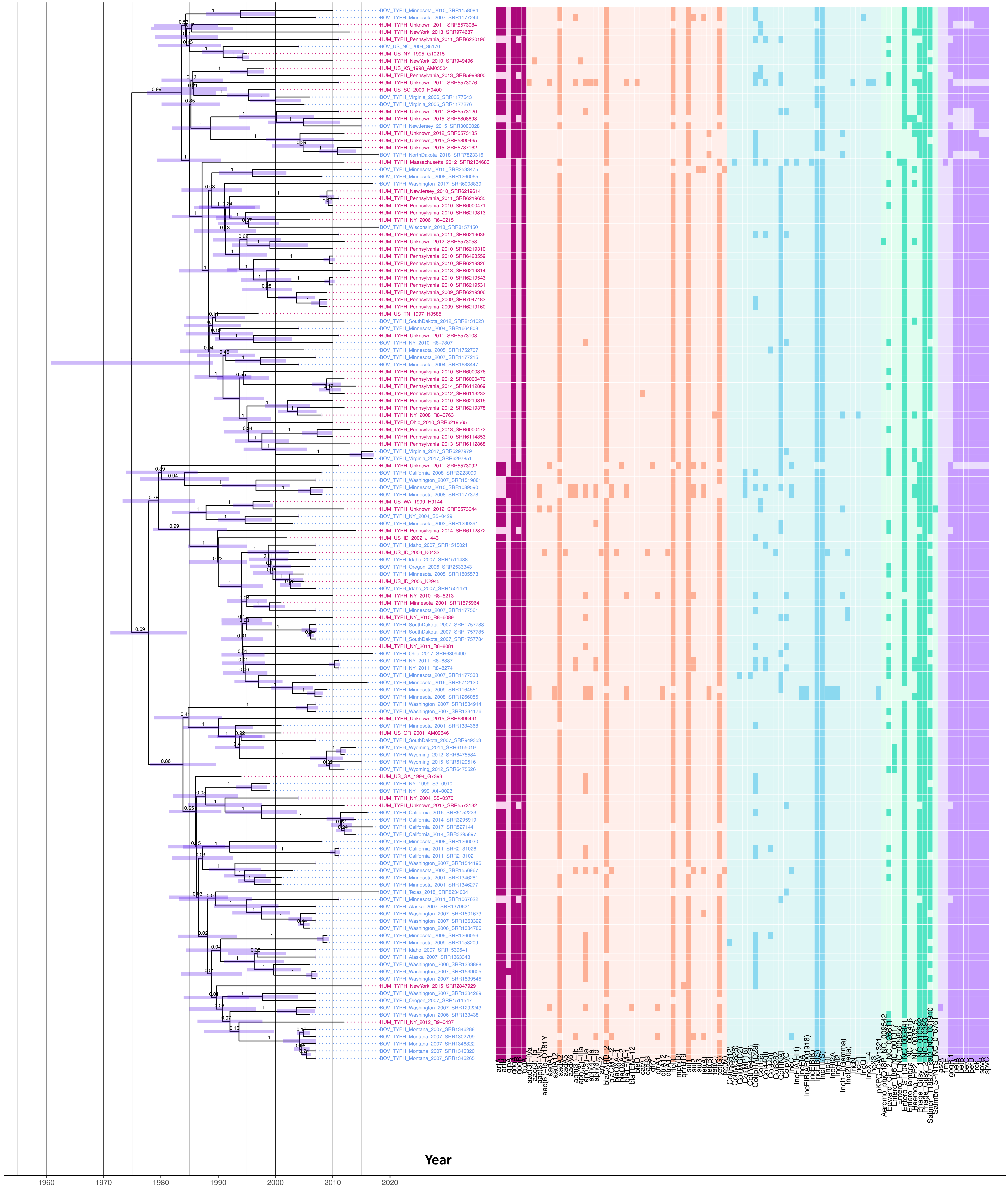

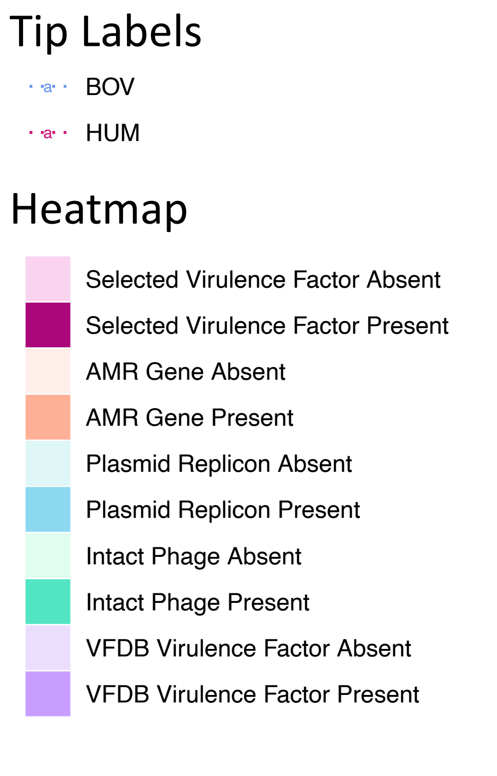

Supplementary Figure S7. Bayesian time-scaled phylogeny constructed using 146 human- and bovine-associated DT104 complex genomes collected in the United States (U.S.; i.e., the genome set downsampled from Dataset 1 [U.S. bovine and human data], using 10 randomly selected DT104 complex genomes collected from cattle in Washington State in 2007). Tip label colors denote the isolation source reported for each genome (human or bovine in pink and blue, respectively). The heatmap to the right of the phylogeny denotes the presence and absence of: (i) selected virulence factors (dark and light pink, respectively; selected virulence factors were detected using nucleotide BLAST and were considered present using a minimum coverage threshold of 40%); (ii) antimicrobial resistance (AMR) genes (dark and light orange, respectively; detected using ABRicate and the NCBI AMR database); (iii) plasmid replicons (dark and light blue, respectively; detected using ABRicate and the PlasmidFinder database); (iv) intact prophage (dark and light green, respectively; identified and classified as "intact" via PHASTER); (v) variably present Virulence Factor Database (VFDB) virulence factors (dark and light purple, respectively; detected using ABRicate and VFDB, with virulence factors detected in all 146 genomes omitted for readability). All analyses that relied on ABRicate employed minimum nucleotide identity and coverage thresholds of 75 and 50%, respectively. The phylogeny was constructed and rooted using BEAST2. Time in years is plotted along the X-axis, while branch labels correspond to posterior probabilities of branch support. Transparent purple node bars denote node height 95% highest posterior density (HPD) intervals.

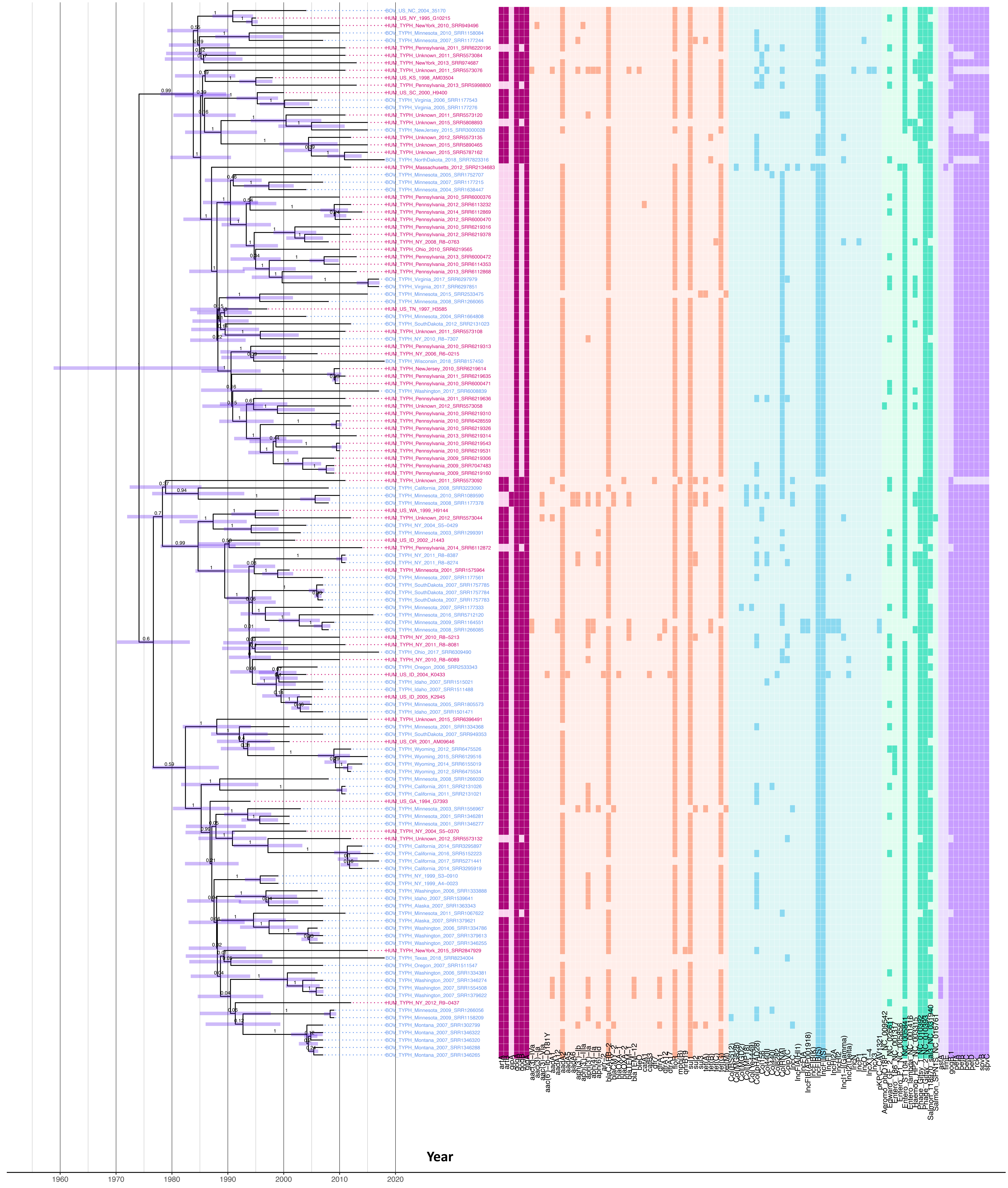

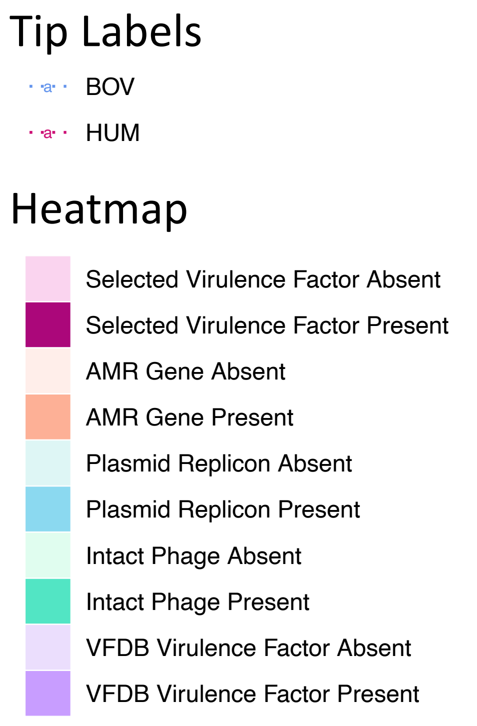

Supplementary Figure S8. Bayesian time-scaled phylogeny constructed using 141 human- and bovine-associated DT104 complex genomes collected in the United States (U.S.; i.e., the genome set downsampled from Dataset 1 [U.S. bovine and human data], using 5 randomly selected DT104 complex genomes collected from cattle in Washington State in 2007). Tip label colors denote the isolation source reported for each genome (human or bovine in pink and blue, respectively). The heatmap to the right of the phylogeny denotes the presence and absence of: (i) selected virulence factors (dark and light pink, respectively; selected virulence factors were detected using nucleotide BLAST and were considered present using a minimum coverage threshold of 40%); (ii) antimicrobial resistance (AMR) genes (dark and light orange, respectively; detected using ABRicate and the NCBI AMR database); (iii) plasmid replicons (dark and light blue, respectively; detected using ABRicate and the PlasmidFinder database); (iv) intact prophage (dark and light green, respectively; identified and classified as "intact" via PHASTER); (v) variably present Virulence Factor Database (VFDB) virulence factors (dark and light purple, respectively; detected using ABRicate and VFDB, with virulence factors detected in all 141 genomes omitted for readability). All analyses that relied on ABRicate employed minimum nucleotide identity and coverage thresholds of 75 and 50%, respectively. The phylogeny was constructed and rooted using BEAST2. Time in years is plotted along the X-axis, while branch labels correspond to posterior probabilities of branch support. Transparent purple node bars denote node height 95% highest posterior density (HPD) intervals.

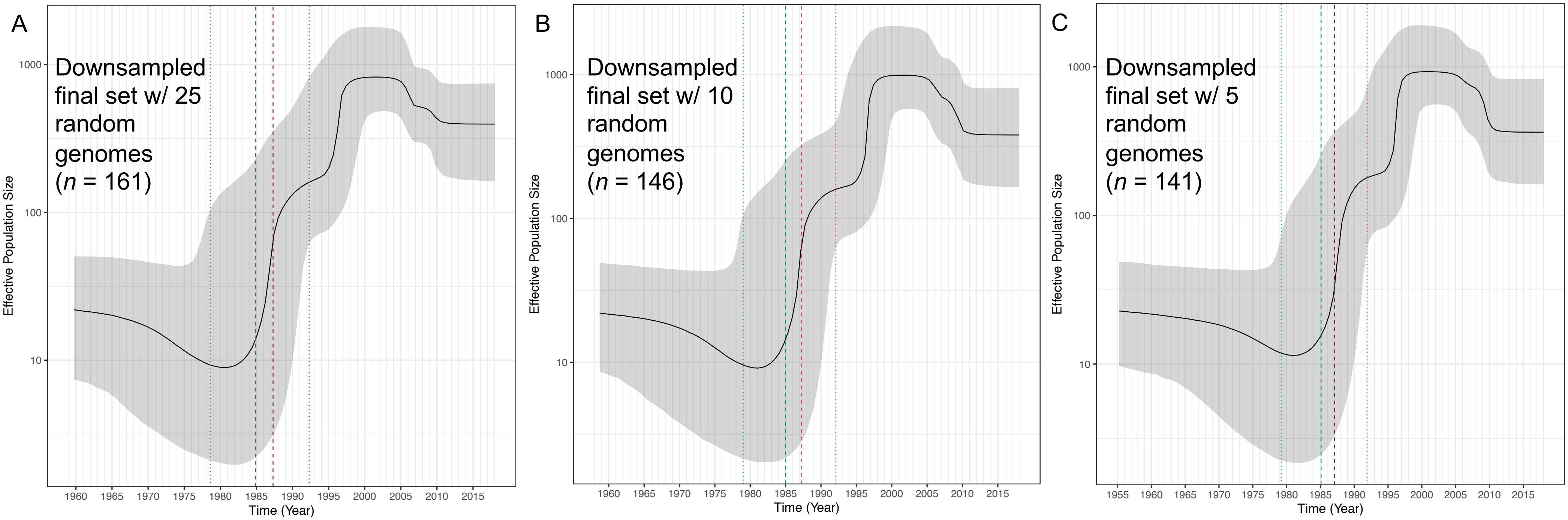

Supplementary Figure S9. Coalescent Bayesian Skyline plots constructed using Dataset 1 (U.S. bovine and human data) genomes, downsampled to (A) 25, (B) 10, and (C) 5 bovine genomes collected from Washington State in 2007 (*n* = 161, 146, and 141 genomes, respectively). Effective population size and time in years are plotted on the Y- and X-axes, respectively. The median effective population size estimate is denoted by the solid black line, with upper and lower 95% highest posterior density (HPD) interval bounds denoted by gray shading. The interval bounded by dashed vertical lines denotes the time interval in which Gifsy-1/*artAB*/*gogB* were predicted to have been lost among members of the U.S. *artAB*-negative major clade. The dotted vertical lines correspond to the 95% HPD interval for Gifsy-1/*artAB*/*gogB* loss among members of the U.S. *artAB*-negative major clade.

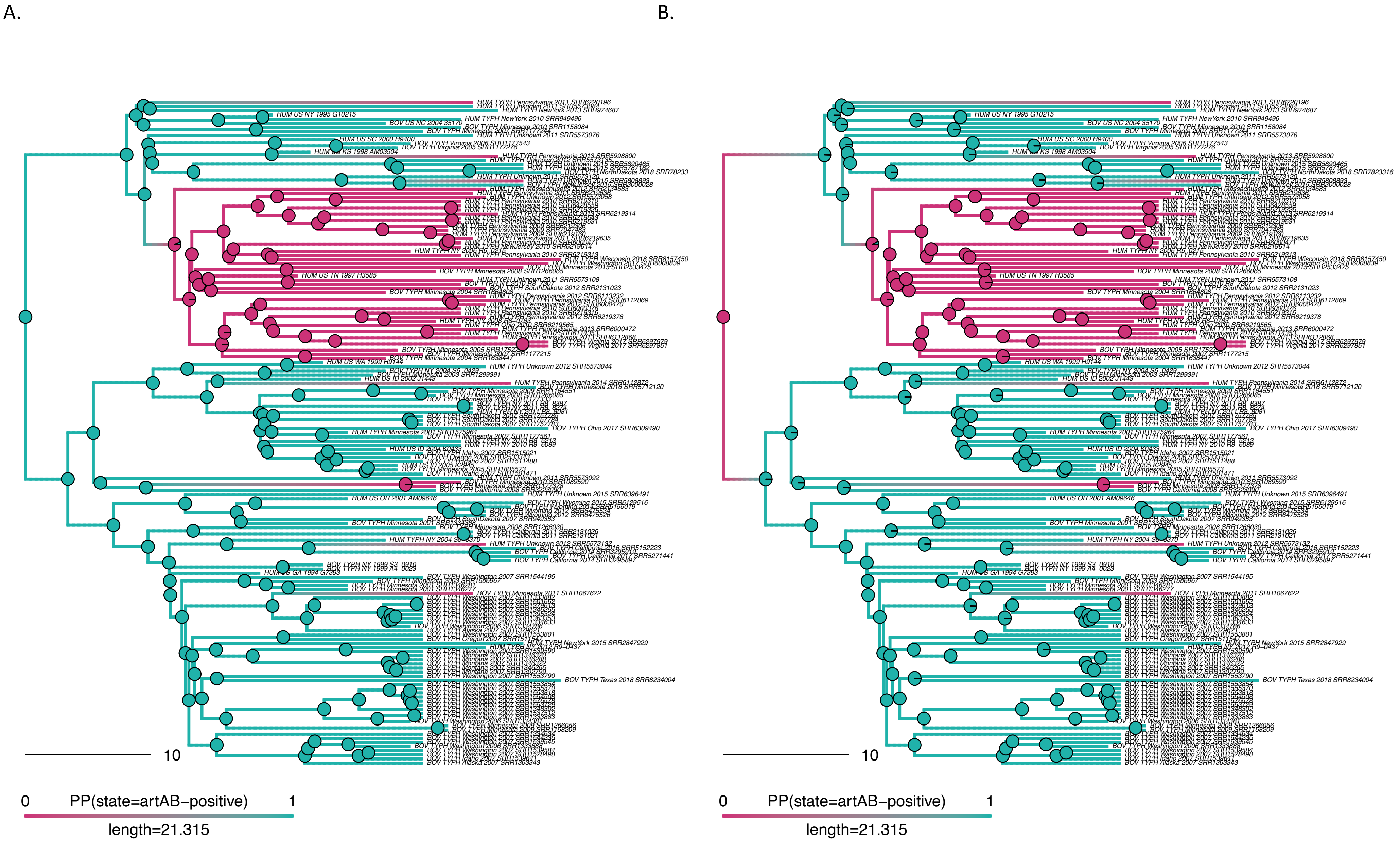

Supplementary Figure S10. Time-scaled Bayesian phylogeny of 161 human- and bovine-associated U.S. DT104 genomes (i.e., the genome set downsampled from Dataset 1 [U.S. bovine and human data], using 25 randomly selected DT104 complex genomes collected from cattle in Washington State in 2007). Tree edge and node colors correspond to the posterior probability (PP) of being in an *artAB*-positive state, obtained using an empirical Bayes approach, in which a continuous-time reversible Markov model was fitted, followed by 10,000 simulations of stochastic character histories using the fitted model and tree tip states. Root node prior probabilities for *artAB*-positive and *artAB*-negative states were (A) equal (i.e., 0.5 each) or (B) estimated using the make.simmap function in the phytools package in R. Branch lengths are reported in years.

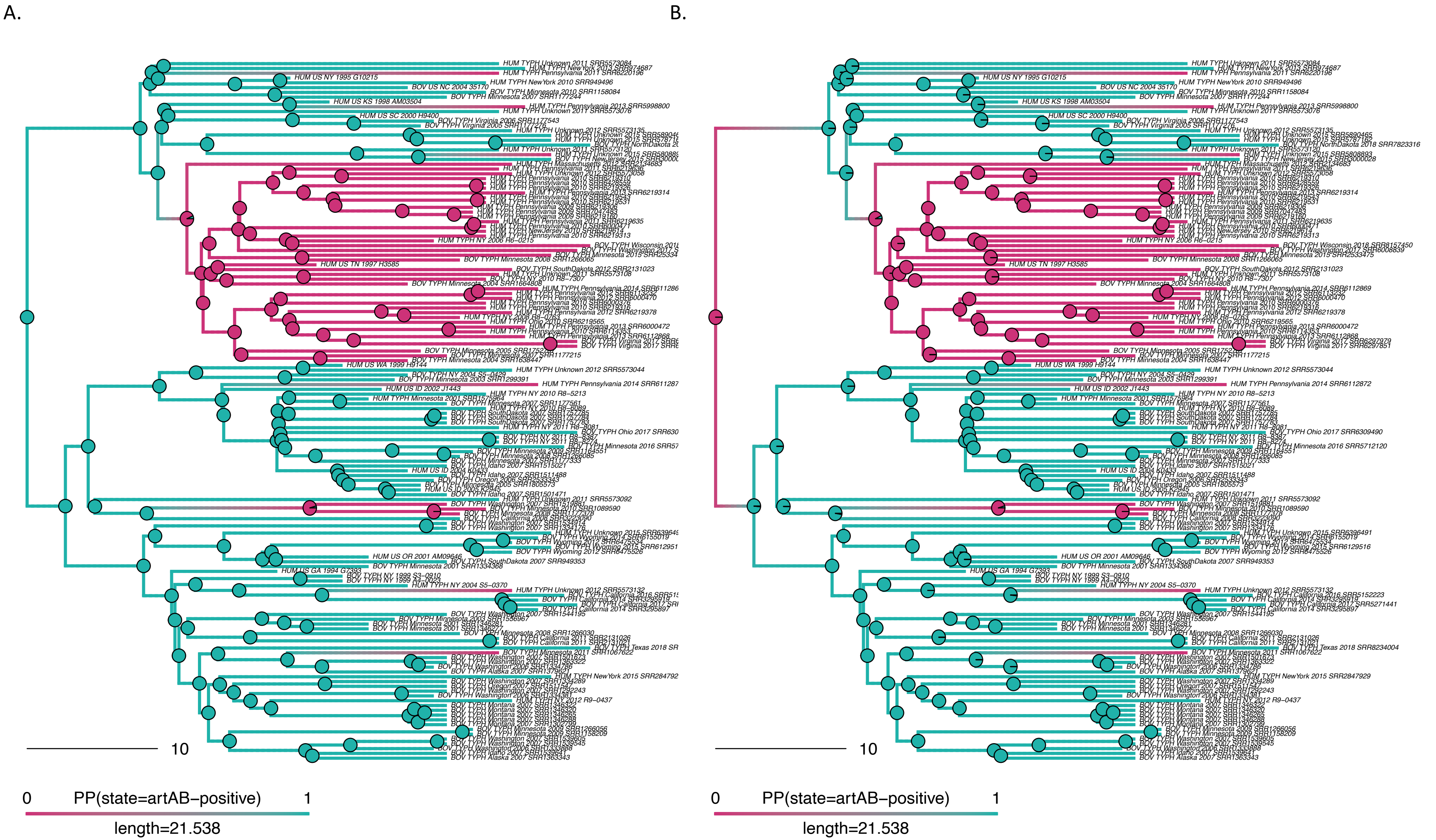

Supplementary Figure S11. Time-scaled Bayesian phylogeny of 146 human- and bovine-associated U.S. DT104 complex genomes (i.e., the genome set downsampled from Dataset 1 (U.S. bovine and human data), using 10 randomly selected DT104 complex genomes collected from cattle in Washington State in 2007). Tree edge and node colors correspond to the posterior probability (PP) of being in an *artAB*-positive state, obtained using an empirical Bayes approach, in which a continuous-time reversible Markov model was fitted, followed by 10,000 simulations of stochastic character histories using the fitted model and tree tip states. Root node prior probabilities for *artAB*-positive and *artAB*-negative states were (A) equal (i.e., 0.5 each) or (B) estimated using the make.simmap function in the phytools package in R. Branch lengths are reported in years.

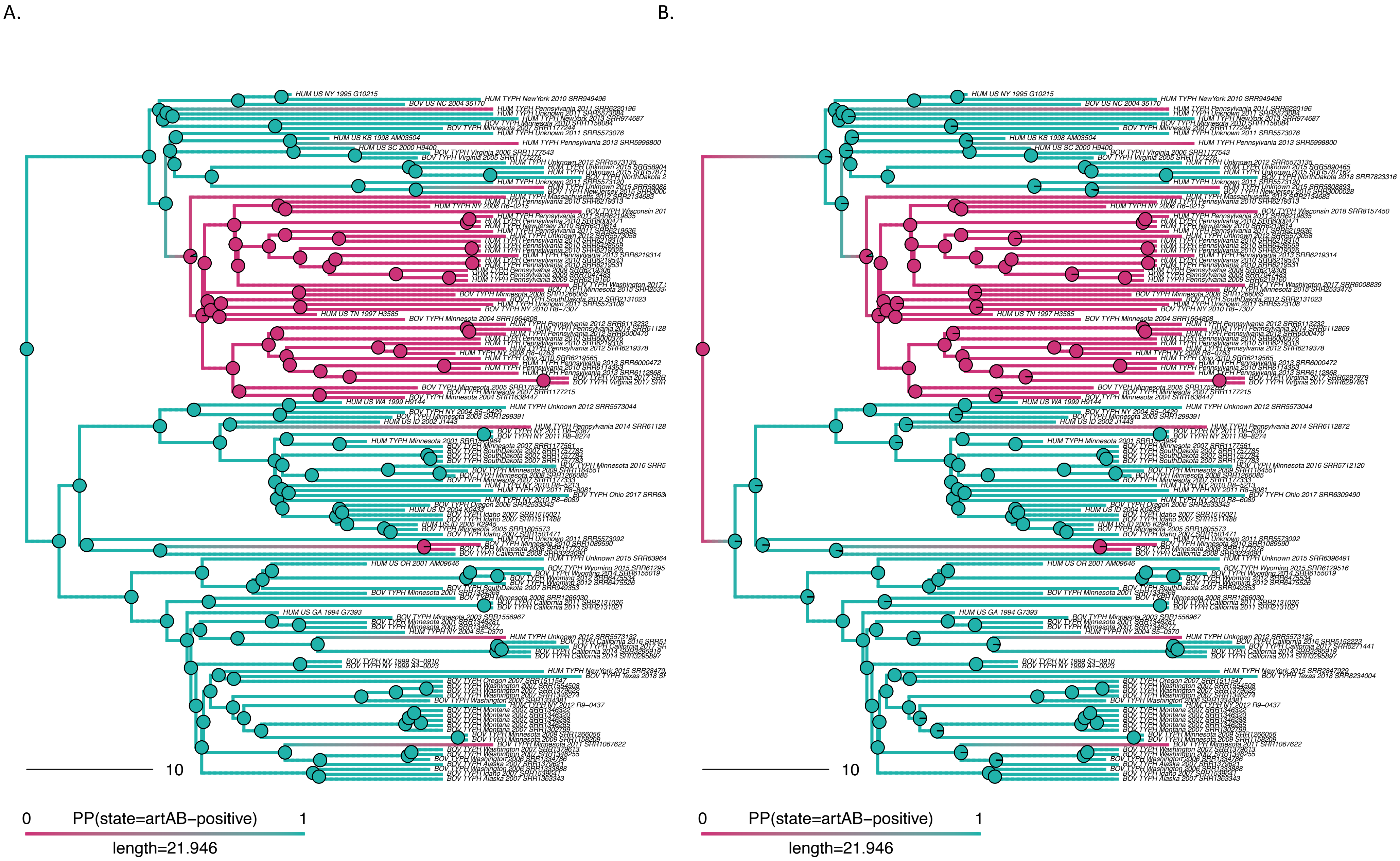

Supplementary Figure S12. Time-scaled Bayesian phylogeny of 141 human- and bovine-associated U.S. DT104 complex genomes (i.e., the genome set downsampled from Dataset 1 (U.S. bovine and human data), using 5 randomly selected DT104 complex genomes collected from cattle in Washington State in 2007). Tree edge and node colors correspond to the posterior probability (PP) of being in an *artAB*-positive state, obtained using an empirical Bayes approach, in which a continuous-time reversible Markov model was fitted, followed by 10,000 simulations of stochastic character histories using the fitted model and tree tip states. Root node prior probabilities for *artAB*-positive and *artAB*-negative states were (A) equal (i.e., 0.5 each) or (B) estimated using the make.simmap function in the phytools package in R. Branch lengths are reported in years.

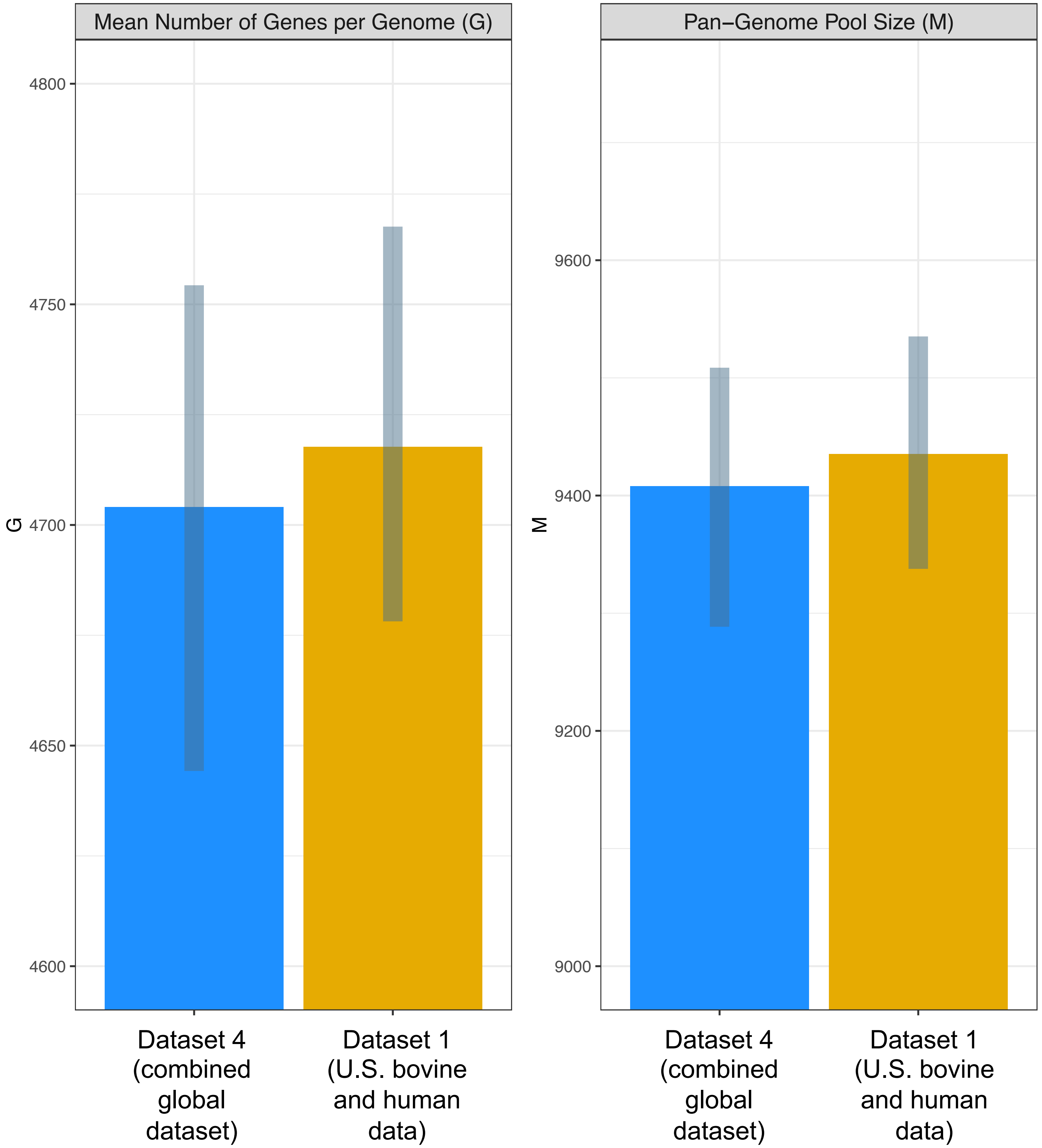

Supplementary Figure S13. Inferred parameters for the Finite Many Genes (FMG) model for the following data sets: (i) Dataset 4 (combined global dataset) (n = 752 DT104 complex genomes); (ii) Dataset 1 (U.S. bovine and human data) (n = 230 DT104 complex genomes). FMG parameters were estimated using Panaroo, with gray bars denoting the 2.5 and 97.5% confidence interval bounds for each parameter (obtained using 100 bootstrap replicates).

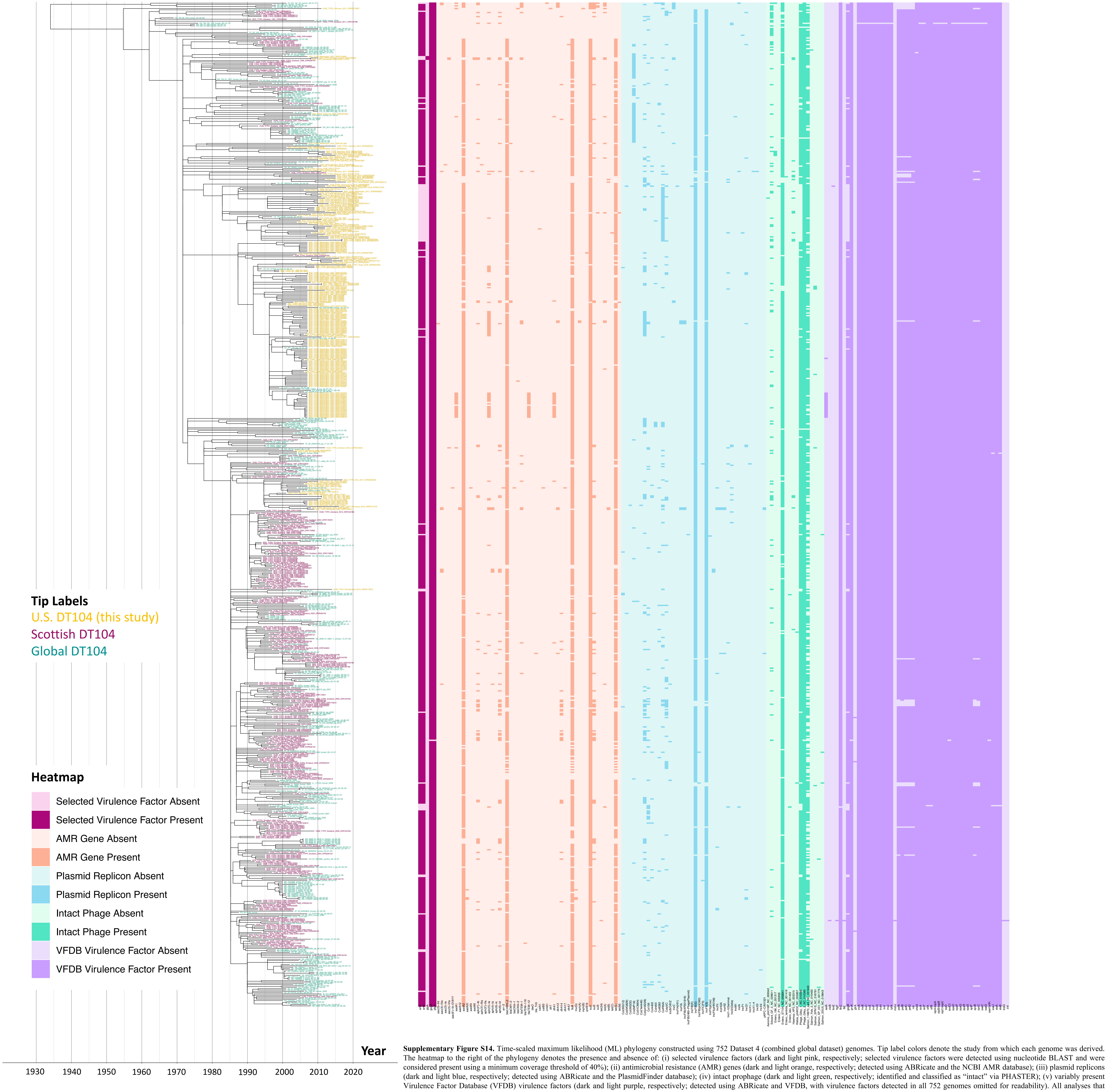

Supplementary Figure S14. Time-scaled maximum likelihood (ML) phylogeny constructed using 752 Dataset 4 (combined global dataset) genomes. Tip label colors denote the study from which each genome was derived. The heatmap to the right of the phylogeny denotes the presence and absence of: (i) selected virulence factors (dark and light pink, respectively; selected virulence factors were detected using nucleotide BLAST and were considered present using a minimum coverage threshold of 40%); (ii) antimicrobial resistance (AMR) genes (dark and light orange, respectively; detected using ABRicate and the NCBI AMR database); (iii) plasmid replicons (dark and light blue, respectively; detected using ABRicate and the PlasmidFinder database); (iv) intact prophage (dark and light green, respectively; identified and classified as "intact" via PHASTER); (v) variably present Virulence Factor Database (VFDB) virulence factors (dark and light purple, respectively; detected using ABRicate and VFDB, with virulence factors detected in all 752 genomes omitted for readability). All analyses that relied on ABRicate employed minimum nucleotide identity and coverage thresholds of 75 and 50%, respectively. The phylogeny was constructed using IQ-TREE and rooted and time-scaled using LSD2. Time in years is plotted along the X-axis.

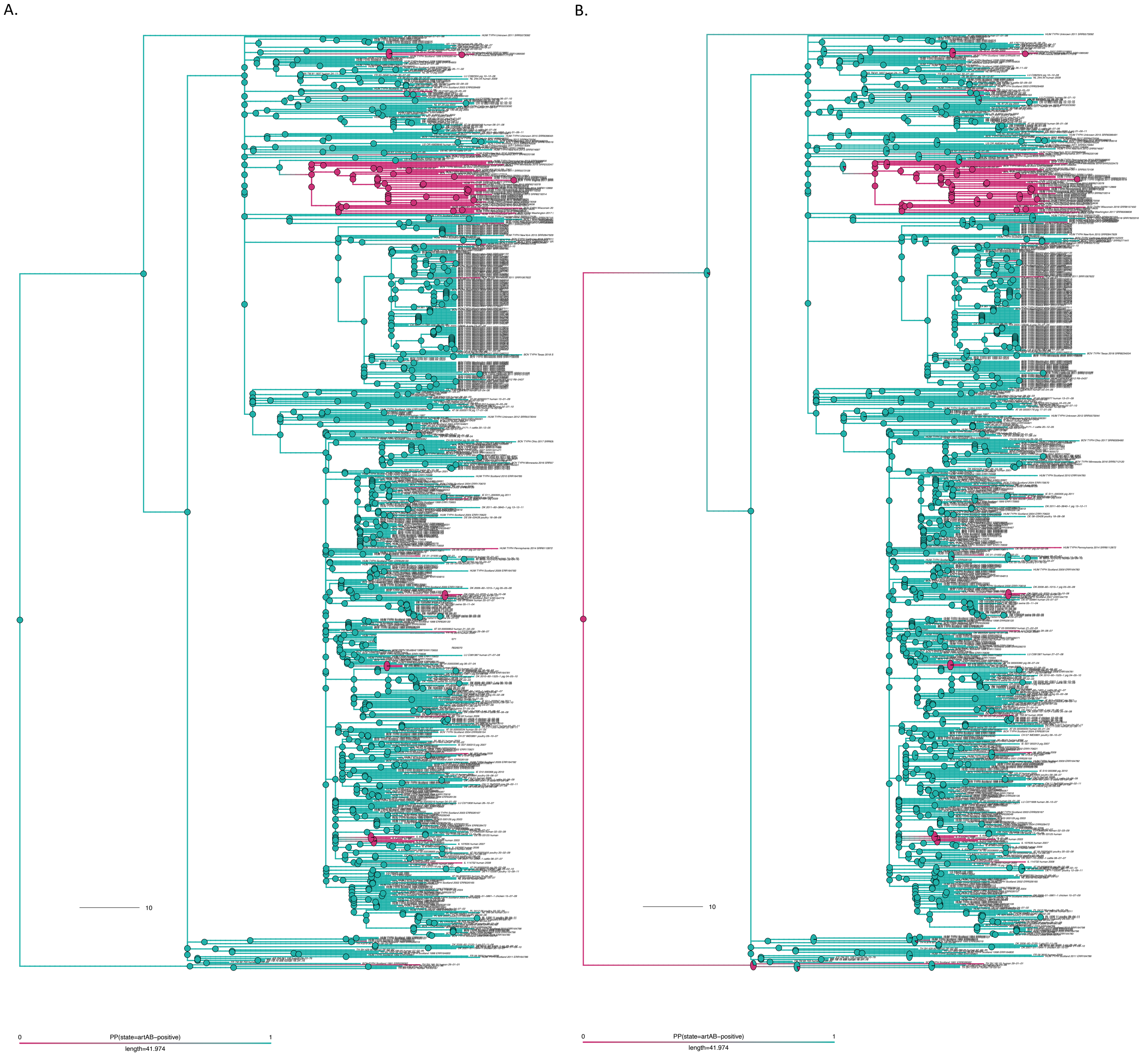

Supplementary Figure S15. Time-scaled maximum likelihood phylogeny of 752 DT104 complex genomes in Dataset 4 (combined global dataset). Tree edge and node colors correspond to the posterior probability (PP) of being in an *artAB*-positive state, obtained using an empirical Bayes approach, in which a continuous-time reversible Markov model was fitted, followed by 10,000 simulations of stochastic character histories using the fitted model and tree tip states. Root node prior probabilities for *artAB*-positive and *artAB*-negative states were (A) equal (i.e., 0.5 each) or (B) estimated using the make.simmap function in the phytools package in R. Branch lengths are reported in years.

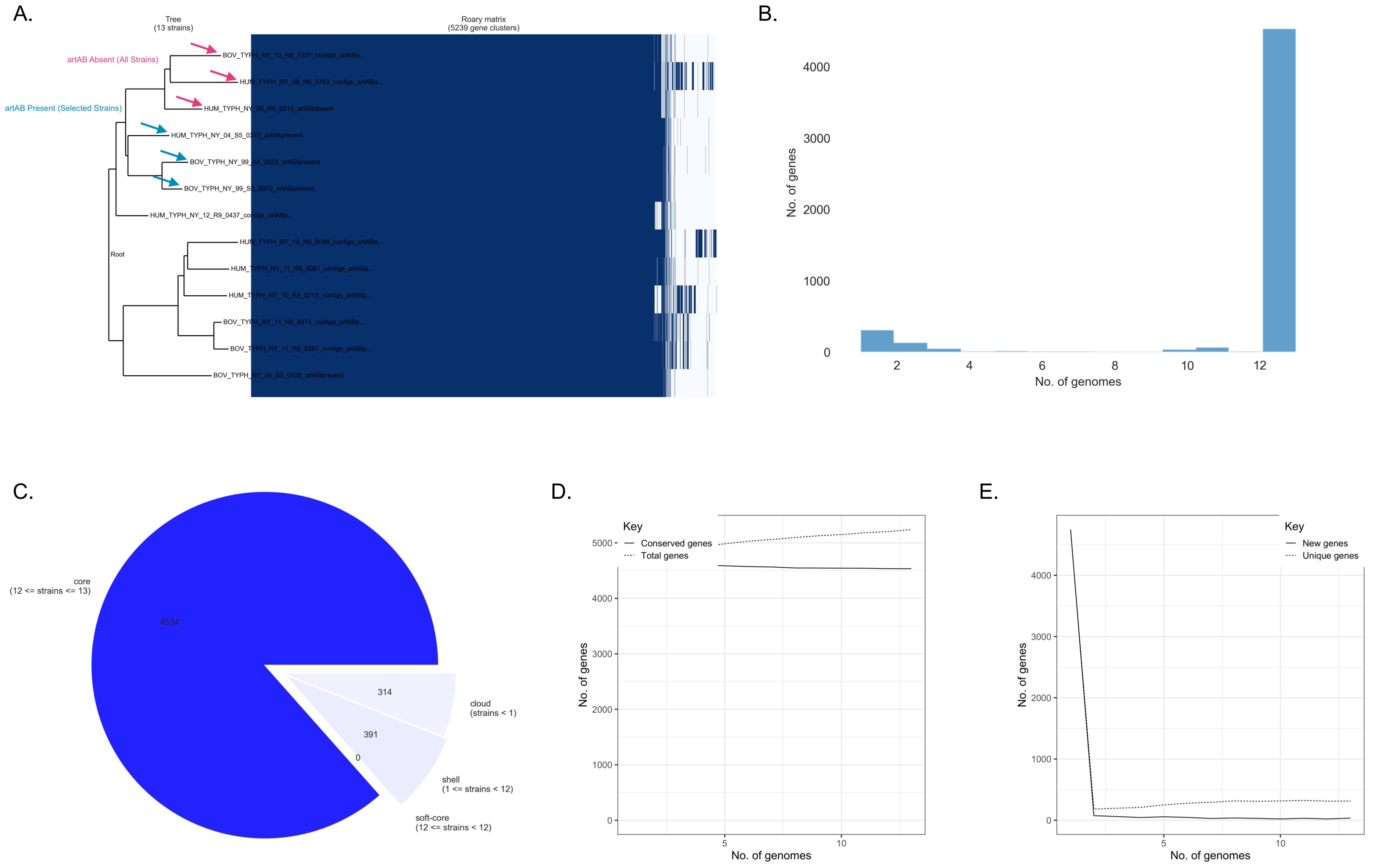

Supplementary Figure S16. (Pan-)genomic characterization of the 13 Cornell University Food Safety Laboratory (CUFSL) DT104 complex strains, which were available for phenotypic assays. (A) Roary gene presence/absence matrix. The phylogeny to the left of the matrix corresponds to the maximum likelihood (ML) phylogeny produced via Parsnp, rooted at the midpoint. *artAB*-positive and *artAB*-negative strains selected to undergo phenotypic characterization are denoted by blue and pink arrows, respectively. (B) Histogram of the number of genes detected among the 13 genomes. (C) Pie chart showcasing pan-/core-genome composition. (D) Number of conserved and total and (E) new and unique genes among the 13 DT104 complex genomes. Plots (A-C) were constructed using roary_plots.py version 0.1.0 (https://github.com/sanger-pathogens/Roary/blob/master/contrib/roary_plots/roary_plots.py). Plots D and E were produced via Roary.

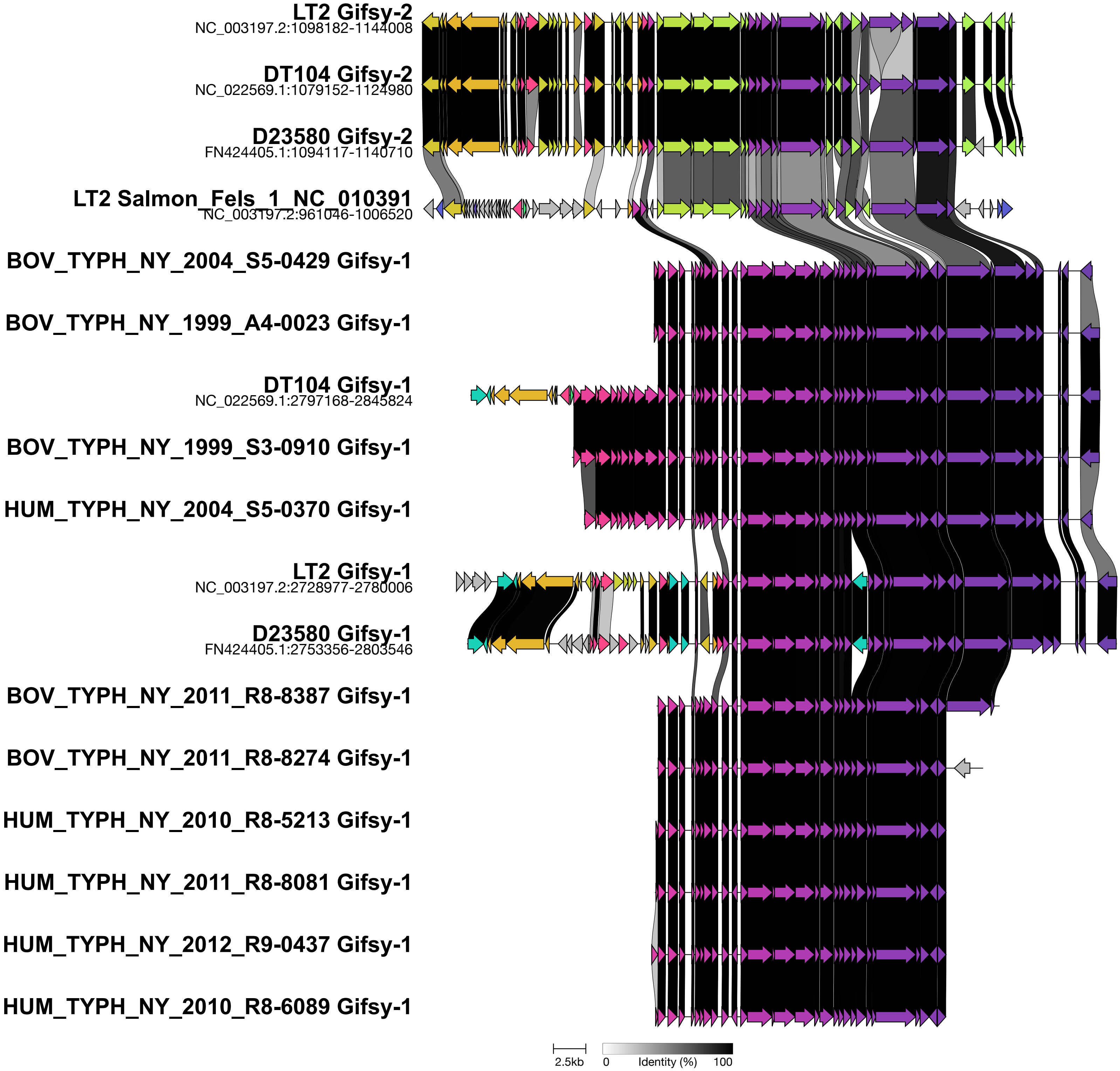

Supplementary Figure S17. artAB-harboring prophages in human- and bovine-associated DT104 complex genomes from New York State, as compared to Gifsy-1- and Gifsy-2-like prophages described in Salmonella Typhimurium strains (i) LT2, (ii) DT104, and (iii) D23580. artAB-harboring prophages were detected in the New York State genomes using PHASTER. Selected LT2, DT104, and D23580 prophage regions were acquired from the PHASTER database. All prophage regions were annotated using Prokka. clinker was used to compare prophage regions using default settings. Arrows correspond to open reading frames (ORFs), with grayscale links denoting the percent (%) amino acid identity shared between corresponding ORFs.

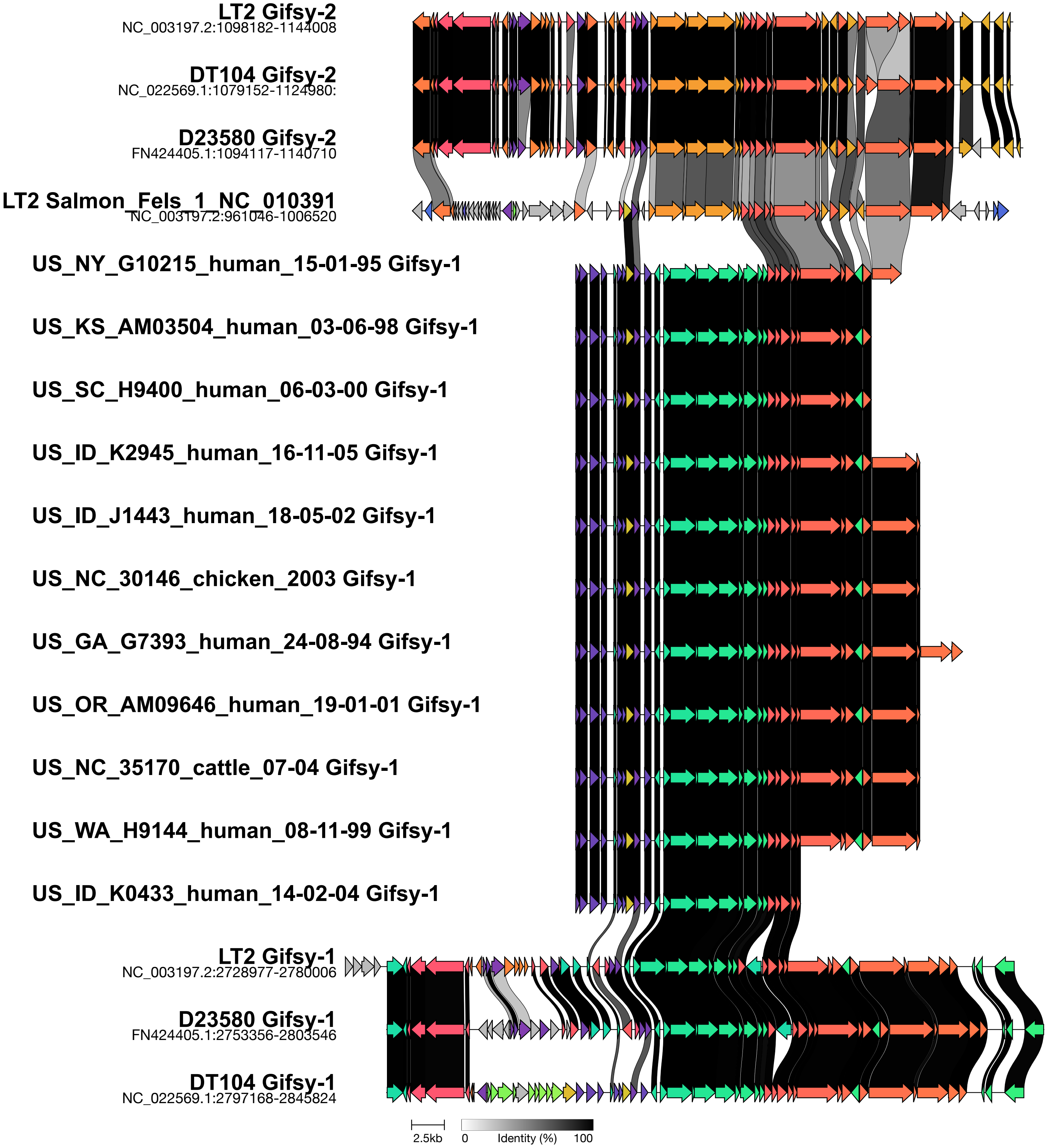

Supplementary Figure S18. artAB-harboring prophages in U.S. DT104 complex genomes from Leekitcharoenphon, et al., 2016, as compared to Gifsy-1- and Gifsy-2-like prophages described in Salmonella Typhimurium strains (i) LT2, (ii) DT104, and (iii) D23580. artAB-harboring prophages were detected in the U.S. genomes from Leekitcharoenphon, et al., 2016 using PHASTER. Selected LT2, DT104, and D23580 prophage regions were acquired from the PHASTER database. All prophage regions were annotated using Prokka. clinker was used to compare prophage regions using default settings. Arrows correspond to open reading frames (ORFs), with grayscale links denoting the percent (%) amino acid identity shared between corresponding ORFs.

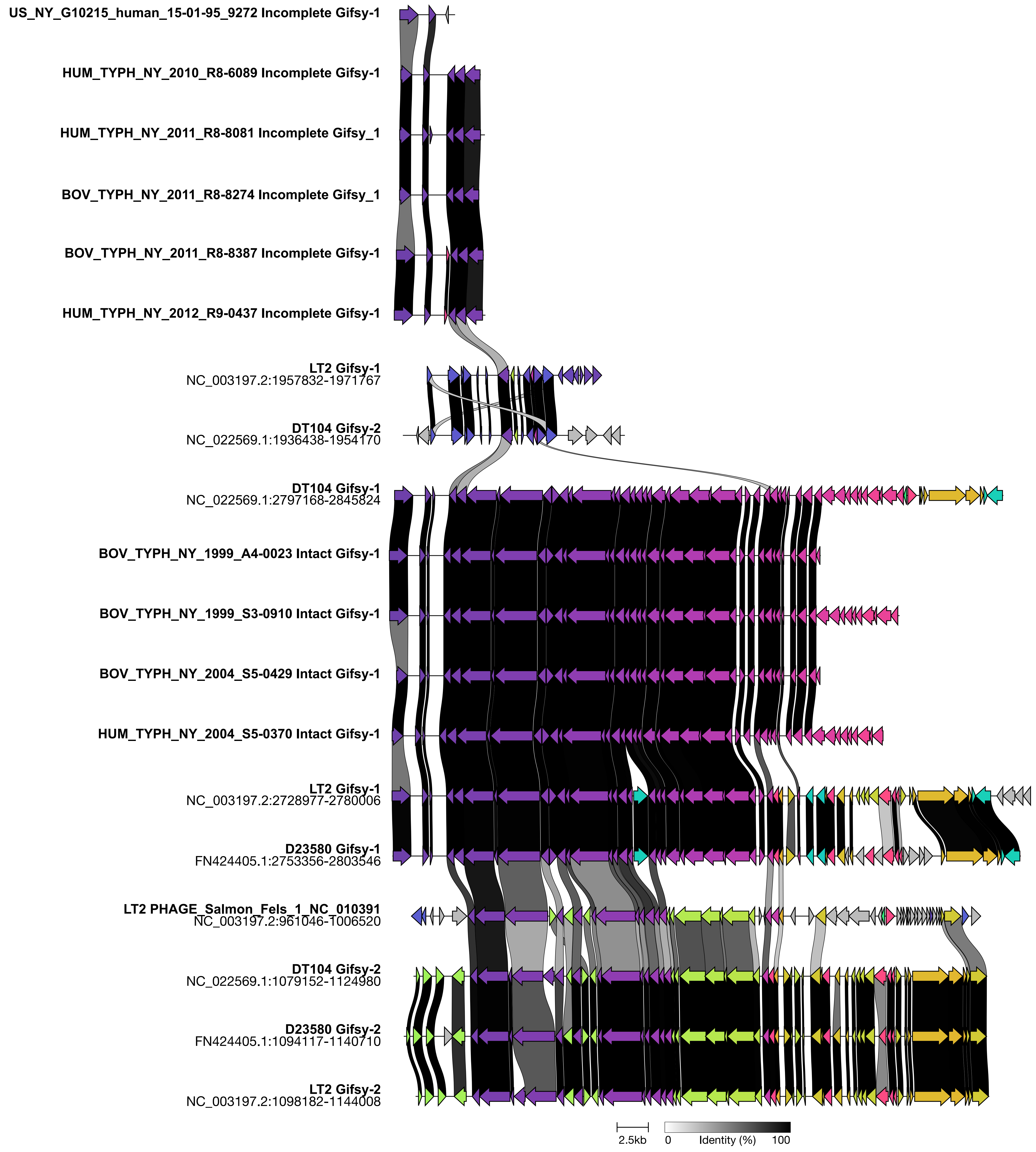

Supplementary Figure S19. gogB-harboring prophage regions in human- and bovine-associated DT104 complex genomes from New York State, as compared to Gifsy-1- and Gifsy-2-like prophages described in Salmonella Typhimurium strains (i) LT2, (ii) DT104, and (iii) D23580. gogB-harboring prophage regions were detected in the New York State genomes using PHASTER. Selected LT2, DT104, and D23580 prophage regions were acquired from the PHASTER database. All prophage regions were annotated using Prokka. clinker was used to compare prophage regions using default settings. Arrows correspond to open reading frames (ORFs), with grayscale links denoting the percent (%) amino acid identity shared between corresponding ORFs.

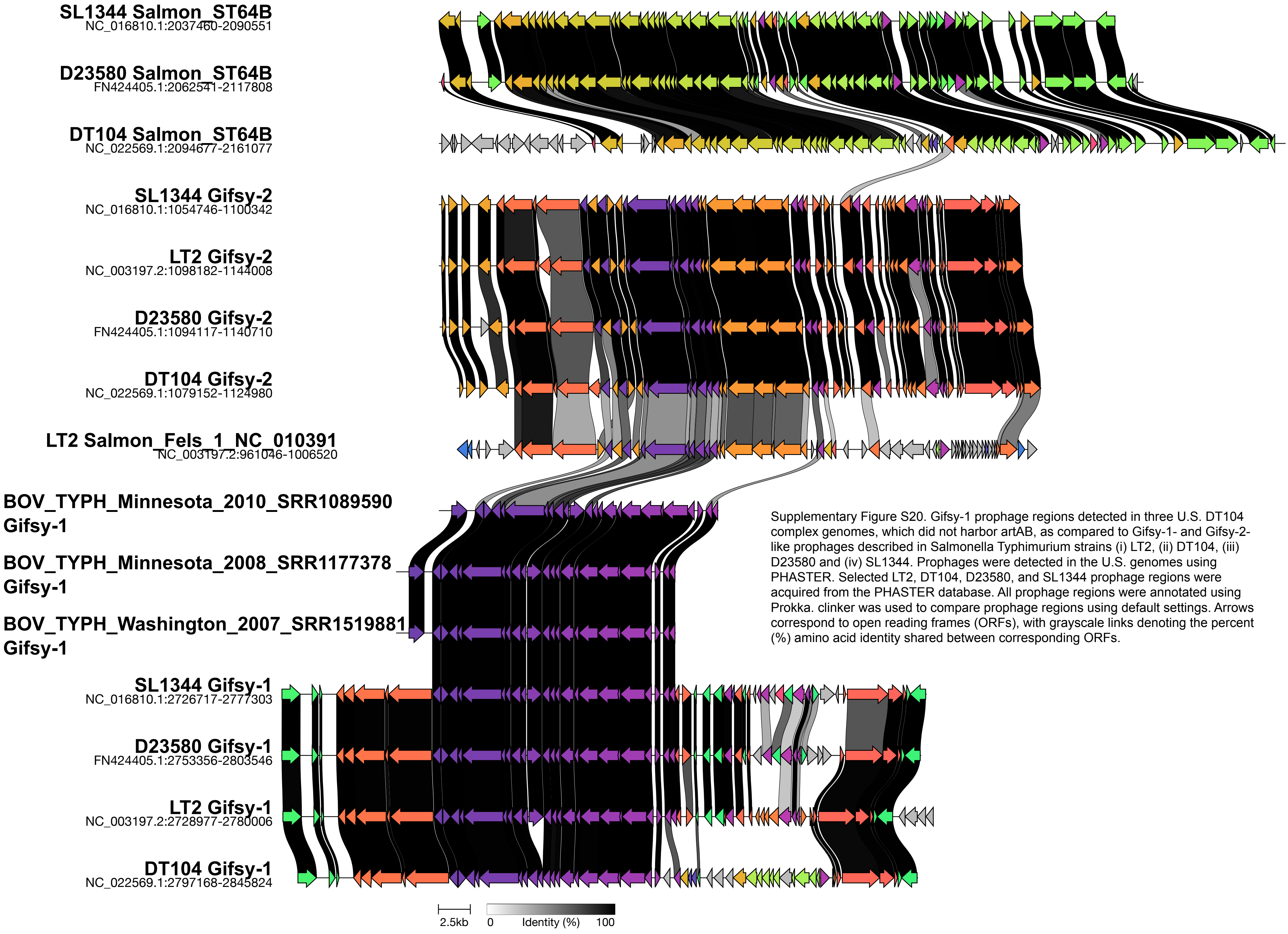

Supplementary Figure S20. Gifsy-1 prophage regions detected in three U.S. DT104 complex genomes, which did not harbor artAB, as compared to Gifsy-1- and Gifsy-2-like prophages described in Salmonella Typhimurium strains (i) LT2, (ii) DT104, (iii) D23580 and (iv) SL1344. Prophages were detected in the U.S. genomes using PHASTER. Selected LT2, DT104, D23580, and SL1344 prophage regions were acquired from the PHASTER database. All prophage regions were annotated using Prokka. clinker was used to compare prophage regions using default settings. Arrows correspond to open reading frames (ORFs), with grayscale links denoting the percent (%) amino acid identity shared between corresponding ORFs.

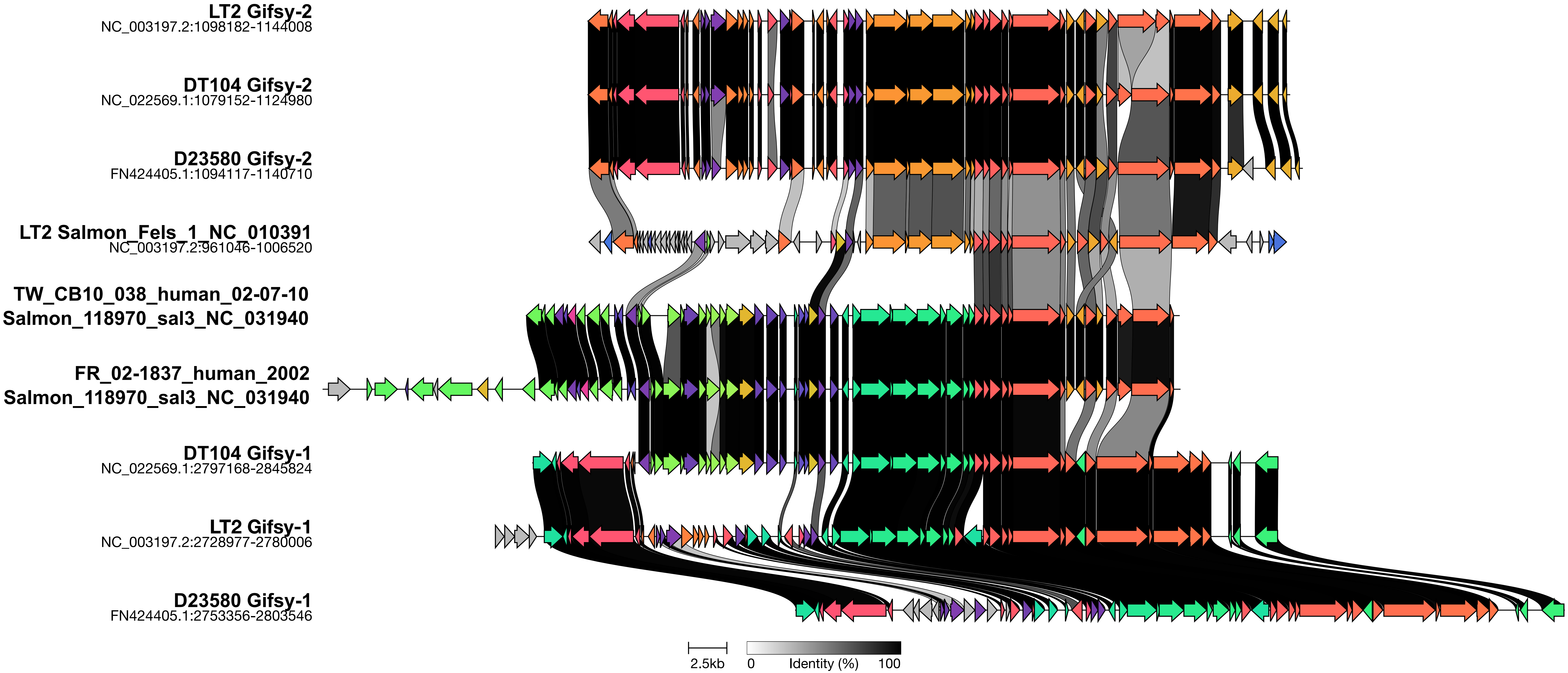

Supplementary Figure S21. artAB-harboring prophages detected in DT104 complex genomes, which were not labeled as “Gifsy-1” by PHASTER, as compared to Gifsy-1- and Gifsy-2-like prophages described in Salmonella Typhimurium strains (i) LT2, (ii) DT104, and (iii) D23580. Selected LT2, DT104, and D23580 prophage regions were acquired from the PHASTER database. All prophage regions were annotated using Prokka. clinker was used to compare prophage regions using default settings. Arrows correspond to open reading frames (ORFs), with grayscale links denoting the percent (%) amino acid identity shared between corresponding ORFs.
