# Supplementary material for: A multidrug-resistant Salmonella enterica Typhimurium DT104 complex lineage circulating among humans and cattle in the USA lost the ability to produce pertussis-like toxin ArtAB
Source: Microb Genom. 2023 Jul 4;9(7):mgen001050. doi: 10.1099/mgen.0.001050 (PMC10438809; doi:10.1099/mgen.0.001050)
Supplement: Supplementary material 3 [file mgen-9-1050-s003.pdf]

SUPPLEMENTARY TEXT

for

A multidrug-resistant *Salmonella enterica* Typhimurium DT104 complex lineage circulating among humans and cattle in the United States lost the ability to produce pertussis-like toxin ArtAB

by

Laura M. Carroll, Nicolo Piacenza, Rachel A. Cheng, Martin Wiedmann, Claudia Guldimann

SUPPLEMENTARY METHODS

Acquisition of U.S. human- and bovine-associated DT104 complex genomic data and metadata. In a previous study of human- and bovine-associated *S.* Typhimurium from New York State, we identified three closely related, *artAB*-negative DT104 complex genomes from both humans and cattle (out of 14 total DT104 complex genomes from humans and cattle in New York State) [1]. Thus, as a first evaluation of *artAB* presence and absence in the DT104 complex, we compiled a set of DT104 complex genomes from humans and cattle across the U.S. (referred to hereafter as "Dataset 1 [U.S. bovine and human data]"; Figure 1B and Supplementary Figures S2 and S3).

To construct Dataset 1 (U.S. bovine and human data), we first collected genomic data derived from 14 human- and bovine-associated DT104 complex isolates from New York State, which we had sequenced in a previous study (members of the *S.* Typhimurium Lineage III cluster described in Supplementary Figures S2 and S5 of Carroll, et al.) [1]. We then aggregated these 14 New York State genomes with 223 human- and bovine-associated DT104 complex genomes from across the U.S., as described previously [1]. Briefly, paired-end Illumina short reads associated with 223 *S.* Typhimurium genomes meeting the following criteria were downloaded via Enterobase (accessed November 29, 2018) [2, 3] and the Sequence Read Archive (SRA) Toolkit version 2.9.3 [4, 5]: (i) genomes were serotyped as *S.* Typhimurium *in silico* using the implementation of SISTR [6] in Enterobase; (ii) the country of isolation was the United States; (iii) the isolation source was reported as either "Human" or "Bovine" in the "Source Niche" and "Source Type" fields in Enterobase, respectively; (iv) genomes had an isolation year reported in Enterobase; (v) using RhierBAPS [7], genomes were assigned to the DT104 complex, a well-supported cluster within the larger bovine- and human-associated U.S. *S.*

Typhimurium phylogeny, which clustered among known DT104 genomes from other countries (see Supplementary Figures S2 and S5 of Carroll, et al.) [1].

Trimmomatic version 0.36 [8] was used to trim low quality bases and Illumina adapters from all read sets using the default settings for paired-end reads, and SPAdes version 3.13.0 [9] was used to assemble all genomes using default settings plus the “careful” option. FastQC version 0.11.5 [10] and QUAST version 4.0 [11] were used to assess the quality of each read pair set and assembly, respectively, and MultiQC version 1.6 [12] was used to aggregate all FastQC and QUAST results. Trimmed paired-end read sets/assemblies that were flagged by MultiQC as meeting any of the following conditions were excluded: (i) Illumina adapters present after trimming (n = 2), (ii) an abnormal per sequence GC content distribution (n = 3), (iii) an assembly with over 200 contigs (n = 11), and (iv) a sequence quality histogram flagged as poor quality (n = 2). After excluding genomes that met these conditions, a set of 219 DT104 complex genomes was produced (Figure 1B, Supplementary Figures S2 and S3, and Supplementary Tables S1 and S2).

Finally, the 219 U.S. human- and bovine-associated DT104 complex genomes identified here were supplemented with 11 U.S. bovine- and human-associated DT104 genomes from a previous study [13], which did not have metadata available in Enterobase at the time and were thus not included in the initial set of 219 bovine- and human-associated U.S. DT104 complex genomes. Overall, the search conducted here produced a set of 230 bovine- and human-associated U.S. DT104 complex genomes, which were used in subsequent steps (i.e., Dataset 1 [U.S. bovine and human data]; Figure 1B, Supplementary Figures S2 and S3, and Supplementary Tables S1 and S2).

***In silico* detection of prophage, antimicrobial resistance genes, plasmid replicons, and virulence factors.** To identify putative prophage regions in all 230 genomes in Dataset 1 (U.S. bovine and human data), each assembly was submitted to the PHASTER web server (https://phaster.ca/) via the URL API [14, 15], with the "contigs" option set to "1" (suggested by PHASTER for multi-contig files in multi-FASTA format, https://phaster.ca/instructions#urlapi, accessed May 13, 2023; Supplementary Table S3). To compare prophage regions identified in Dataset 1 (U.S. bovine and human data) genomes to previously described prophage in well-characterized *S.* Typhimurium strains, prophage in the following *S.* Typhimurium strains were obtained from the PHASTER prophage database (accessed September 18, 2020): (i) LT2 (NCBI Nucleotide accession NC_003197.2), (ii) DT104 (NCBI Nucleotide accession NC_022569.1), (iii) D23580 (NCBI Nucleotide accession FN424405.1), and (iv) SL1344 (NCBI Nucleotide accession NC_016810.1; Figure 1A and Supplementary Figure S1). All prophage regions were annotated using Prokka version 1.14.6 [16], using default settings and the "Viruses" kingdom database. The resulting GFF and FNA files produced by Prokka were supplied to clinker version 0.0.26 [17], which was used to perform pairwise alignments of all genes within prophage regions using default settings.

ABRicate version 0.8 [18] was used to detect antimicrobial resistance (AMR) genes, plasmid replicons, and virulence factors in each assembled DT104 genome using NCBI's National Database of Antibiotic Resistant Organisms (NDARO) [19], the PlasmidFinder database [20], and the Virulence Factor Database (VFDB) [21], respectively, using minimum nucleotide identity and coverage thresholds of 75 and 50%, respectively (all databases accessed December 10, 2020; Supplementary Table S3). The aforementioned ABRicate analyses were

repeated, using a minimum coverage threshold of 0% (e.g., to confirm that virulence factors discussed in the manuscript were absent from genomes in which they were not initially detected).

Each assembled genome was additionally queried for the presence of selected virulence factors, which have previously been associated with prophage in *Salmonella* [22]: (i) *artAB* (NCBI Nucleotide Accession AB104436.1), (ii) *gogA* (European Nucleotide Archive [ENA] Accession EAA7850902.1), (iii) *gtgA* (ENA Accession PVI70081.1), and (iv) *gipA* (ENA Accession CAI93790.1). Assembled genomes were queried for selected virulence factors using the command-line implementation of nucleotide BLAST (blastn) version 2.11.0 [23], using default settings plus a minimum coverage threshold of 40% (Supplementary Table S3). To confirm that the aforementioned genes were absent from genomes in which they were not initially detected, all genomes were queried again (i) as described above, with the coverage threshold lowered to 0%; and (ii) using translated nucleotide BLAST (tblastx; Supplementary Tables S4 and S5).

ARIBA version 2.14.6 [24] was used to further confirm *artAB* and *gogB* presence/absence in all genomes with associated paired-end Illumina reads (*n* = 219 U.S. human- and bovine-associated DT104 complex genomes from Dataset 1 [U.S. bovine and human data] with paired-end Illumina reads; Figure 1B, Supplementary Figures S2 and S3, and Supplementary Tables S1 and S2). Briefly, the ARIBA database was constructed using the ARIBA "prepareref" command (default settings, except "--all_coding" set to "yes" and the "--no_cdhit" option included), and ARIBA was run using ARIBA's "run" command with default settings. *artAB* presence/absence results obtained via ARIBA were identical to results obtained via blastn (Supplementary Table S6). For *gogB*, ARIBA results matched BLAST results for all but five genomes (*n* = 214 of 219 genomes, 97.7%; Supplementary Table S6). For the five

discordant genomes, *gogB* was detected via blastn, but not with ARIBA. Upon manual inspection of these five genomes, (i) three genomes had *gogB* split on multiple contigs, and (ii) two genomes had an intact *gogB* detected at high identity (>99.9% nucleotide identity) and 100% coverage via blastn. blastn results are thus reported in the main manuscript (Supplementary Tables S3-S5), with ARIBA results available in Supplementary Table S6.

Variant calling and maximum likelihood phylogeny construction within Dataset 1 (U.S. bovine and human data). Core single nucleotide polymorphisms (SNPs) were identified among all 230 genomes within Dataset 1 (U.S. bovine and human data), using the default pipeline implemented in Snippy version 4.6.0 [25] and the following dependencies: BWA version 0.7.17-r1188 [26, 27], Minimap2 version 2.23-r1111 [28], SAMtools version 1.14 [29], BEDtools version 2.30.0 [30, 31], BCFtools version 1.14 [32], FreeBayes version 1.3.2-dirty [33], vcflib version 1.0.0-rc0-349-g45c6-dirty [34], vt version 0.5 [35], SnpEff version 5.0e [36], samclip version 0.4.0 [37], seqtk version 1.3-r106 [38], and snp-sites version 2.5.1 [39]. For the 219 Dataset 1 (U.S. bovine and human data) genomes with paired-end Illumina reads available, the trimmed Illumina paired-end reads associated with each genome were used as input; for the remaining 11 genomes, the assembled contigs were used as input (see section "Acquisition of U.S. human- and bovine-associated DT104 complex genomic data and metadata" above; Supplementary Tables S1 and S2). The closed DT104 chromosome (NCBI Nucleotide accession NC_022569.1) was used as a reference. Core SNPs identified in regions of the DT104 chromosome predicted to belong to phage were masked (see section "*In silico* detection of prophage, antimicrobial resistance genes, plasmid replicons, and virulence factors" above). Gubbins version 2.4.1 [40] was used to identify and remove recombination events in all genomes

using default settings, and snp-sites was used to query the resulting recombination-free alignment for core SNPs (i.e., using the "-c" option).

A maximum likelihood (ML) phylogeny was constructed with IQ-TREE version 1.5.4 [41], using (i) the resulting core SNPs as input, (ii) the optimal nucleotide substitution model determined using Bayesian information criteria (BIC) values produced with ModelFinder [42] (i.e., the K3Pu+I model) [43], (iii) an ascertainment bias correction to account for the use of solely variant sites (corresponding to constant sites identified relative to the DT104 reference chromosome; "-fconst 1092869,1195194,1193287,1094079"), and (iv) 1,000 replicates of the ultrafast bootstrap approximation [44, 45].

TempEst version 1.5.3 [46] was used to assess the temporal structure of the resulting unrooted ML phylogeny, using the best-fitting root and the R^2 function ($R^2 = 0.33$, slope = 3.05×10^{-7} substitutions/site/year, X-intercept = 1988.1). The unrooted ML phylogeny was additionally rooted and time scaled using LSD2 version 1.4.2.2 [47] and the following parameters: (i) tip dates corresponding to the year of isolation associated with each genome; (ii) an estimated substitution rate; (iii) constrained mode ("-c"), with the root estimated using constraints on all branches ("-r as"); (iv) variances calculated using input branch lengths ("-v 1"); (v) 1,000 samples for calculating confidence intervals for estimated dates ("-f 1000"); (vi) a sequence length of 4,500,000. The resulting rooted, time-scaled ML phylogeny was viewed using FigTree version 1.4.4 [48] (Supplementary Data).

Dataset 1 (U.S. bovine and human data) Bayesian time-scaled phylogeny construction. In addition to constructing a time-scaled ML phylogeny (see section "Variant calling and maximum likelihood phylogeny construction within Dataset 1 [U.S. bovine and human data]" above), a Bayesian approach was additionally employed to construct time-scaled phylogenies for Dataset 1

(U.S. bovine and human data). Due to an overrepresentation of strains reportedly isolated in 2007 from bovine sources in Washington State within Dataset 1 (U.S. bovine and human data) (n = 94 of 230 Dataset 1 [U.S. bovine and human data] genomes, 40.9%; Figure 2, Supplementary Figure S4, and Supplementary Table S1), all aforementioned SNP calling and ML phylogeny construction steps were repeated among Dataset 1 (U.S. bovine and human data) genome sets downsampled to (i) 25, (ii) 10, and (iii) 5 randomly selected bovine genomes collected in Washington State in 2007 (to minimize the risk of parameter estimate biases during Bayesian phylogeny construction, n = 161, 146, and 141 total genomes in each downsampled genome set, respectively, out of 230 total Dataset 1 [U.S. bovine and human data] genomes; see section "Variant calling and maximum likelihood phylogeny construction within Dataset 1 [U.S. bovine and human data]" above, Supplementary Data) [49]. All resulting ML phylogenies were time-scaled using TempEst and LSD2 as described above (see section "Variant calling and maximum likelihood phylogeny construction within Dataset 1 [U.S. bovine and human data]" above; Supplementary Table S7).

For each of the three downsampled Dataset 1 (U.S. bovine and human data) genome sets, BEAST2 version 2.5.1 [50, 51] was used to construct a tip-dated phylogeny, using core SNPs detected among the genomes within the respective downsampled data set as input (see section "Variant calling and maximum likelihood phylogeny construction within Dataset 1 [U.S. bovine and human data]" above). For all three downsampled genome sets, an initial clock rate of 2.79×10^{-7} substitutions/site/year [13] was used, along with an ascertainment bias correction to account for the use of solely variant sites [52]. bmodeltest [53] was used to infer a substitution model using Bayesian model averaging, with transitions and transversions split. A relaxed lognormal molecular clock [54] and a coalescent Bayesian skyline population model [55] were

used, as these models have been selected as the optimal clock/population model combination for DT104 previously [13]. A log-normal distribution with a mean of 4.6×10^{-7} and standard deviation of 1 (median of 2.79×10^{-7}) was used as the prior on the uncorrelated log-normal relaxed molecular clock mean rate parameter (ucld.mean; Supplementary Data).

For each of the three downsampled Dataset 1 (U.S. bovine and human data) genome sets, five independent BEAST2 runs (i.e., BEAST2 runs with different random seeds) were performed, using chain lengths of at least 100 million generations, sampling every 10 thousand generations. For each downsampled data set, LogCombiner-2 was used to aggregate the resulting log and tree files with 10% of the states treated as burn-in, and TreeAnnotator-2 was used to produce a maximum clade credibility (MCC) tree using Common Ancestor node heights (Supplementary Data). The resulting phylogenies were displayed and annotated using R version 4.1.2 [56] and the following packages: ggplot2 version 3.3.5 [57], ggtree version 3.2.1 [58, 59], phylobase version 0.8.10 [60], and treeio version 1.18.1 [61].

All three downsampled Dataset 1 (U.S. bovine and human data) genome sets resulted in similar BEAST2 parameter estimates (Supplementary Figure S5, Supplementary Table S8, and Supplementary Data). The final Bayesian time-scaled phylogeny and associated parameter estimates reported in the main manuscript correspond to those obtained using the Dataset 1 (U.S. bovine and human data) genome set, which was downsampled to 10 randomly selected bovine genomes collected in Washington State in 2007 (n = 146 genomes, Figures 3 and 4). Results are available for the Dataset 1 (U.S. bovine and human data) genome sets downsampled to 25 and 5 bovine genomes collected in Washington State in 2007 (Supplementary Figures S6-S9, Supplementary Table S8, and Supplementary Data).

***artAB* ancestral state reconstruction for Dataset 1 (U.S. bovine and human data).** To estimate ancestral character states of internal nodes in the Dataset 1 (U.S. bovine and human data) phylogeny as they related to *artAB* presence/absence (i.e., whether a node in the tree represented an ancestor that was more likely to be *artAB*-positive or *artAB*-negative), the presence or absence of *artAB* within each genome was treated as a binary state (see section "*In silico* detection of prophage, antimicrobial resistance genes, plasmid replicons, and virulence factors" above). Three separate *artAB* ancestral state reconstruction runs were performed, each using one of the three BEAST2 time-scaled Bayesian Dataset 1 (U.S. bovine and human data) phylogenies as input (*n* = 161, 146, and 141 total genomes in each downsampled Dataset 1 [U.S. bovine and human data] genome set; see section "Dataset 1 [U.S. bovine and human data] Bayesian time-scaled phylogeny construction" above).

Stochastic character maps were simulated on each phylogeny using the make.simmap function in the phytools version 1.0-1 R package [62] and the all-rates-different (ARD) model in the ape version 5.6-1 package [63, 64]. For each phylogeny, either (i) equal root node prior probabilities for *artAB*-positive and *artAB*-negative states (i.e., *P*(*artAB* present) = *P*(*artAB* absent) = 0.5), or (ii) estimated root node prior probabilities for *artAB*-positive and *artAB*-negative states obtained using the make.simmap function were used. For each root node prior/phylogeny combination (six total combinations of two root node priors and three Dataset 1 [U.S. bovine and human data] phylogenies), an empirical Bayes approach was used, in which a continuous-time reversible Markov model was fitted, followed by 10,000 simulations of stochastic character histories using the fitted model and tree tip states. The resulting phylogenies were plotted using the densityMap function in the phytools R package. For Dataset 1 (U.S. bovine and human data), the final ancestral state results reported in the main manuscript

correspond to those obtained using the Dataset 1 (U.S. bovine and human data) genome set, which was downsampled to 10 randomly selected bovine genomes collected in Washington State in 2007 (*n* = 146 genomes, Figure 3). Results are available for the Dataset 1 (U.S. bovine and human data) genome sets downsampled to 25 and 5 bovine genomes collected in Washington State in 2007 (Supplementary Figures S10-S12 and Supplementary Data).

Pan-genome characterization of Dataset 1 (U.S. bovine and human data). Prokka version 1.13.3 [16] was used to annotate all 230 genomes within Dataset 1 (U.S. bovine and human data), using the "Bacteria" database and default settings (Supplementary Tables S1 and S2). GFF files produced by Prokka were supplied as input to Panaroo version 1.2.7 [65], which was used to identify core- and pan-genome orthologous gene clusters among the 230 Dataset 1 (U.S. bovine and human data) genomes, with the following parameters: (i) "strict" mode ("--clean-mode strict"); (ii) MAFFT as the sequence aligner ("--aligner mafft") [66, 67]; (iii) a core genome threshold of 98% (i.e., genes present in at least 98% of genomes were considered to be core genes; "--core_threshold 0.98"); (iv) a protein family sequence identity threshold of 70% ("-f 0.7", the default). The LSD2 time-scaled ML phylogeny for Dataset 1 (U.S. bovine and human data) (see section "Variant calling and maximum likelihood phylogeny construction within Dataset 1 [U.S. bovine and human data]" above) was supplied as input to Panaroo's "panaroo-img" and "panaroo-fmg" commands, which were used to estimate the pan-genome size under the Infinitely Many Genes (IMG) [68, 69] and Finite Many Genes (FMG) models (with 100 bootstrap replicates) [70], respectively (Supplementary Figure S13).

Reference pan-genome coding sequences (CDS) identified by Panaroo underwent functional annotation using the eggNOG-mapper version 2 webserver (http://eggnog-mapper.embl.de/; accessed July 24, 2022) using default settings [71, 72]. The "table" function in

R was used to identify genes associated with (i) prophage Gifsy-1 presence/absence (Supplementary Table S9) and (ii) clade membership (Supplementary Table S10); the "fisher.test" function in R's stats package was used to conduct two-sided Fisher's exact tests, and the "p.adjust" function was used to control the false discovery rate (i.e., p.adjust method = "fdr") [73].

Genome-wide identification of host-associated orthologous gene clusters for Dataset 1 (U.S. bovine and human data). The treeWAS version 1.0 R package [74] was used to identify potential orthologous gene cluster-host associations among the 230 human- and bovine-associated U.S. DT104 complex genomes in Dataset 1 (U.S. bovine and human data) (i.e., whether an orthologous gene cluster identified with Panaroo was human- or bovine-associated while accounting for population structure). The following treeWAS parameters were used: (i) the isolation source was treated as a discrete phenotype (i.e., a vector of "human" or "bovine", supplied to the "treeWAS" function's "phen" argument; phen.type = "discrete"); (ii) unique gene presence/absence profiles of genes detected in ≥10 and ≤220 of 230 total Dataset 1 (U.S. bovine and human data) genomes, treated as the genotypes to test (supplied to the treeWAS function's "snps" argument); (iii) the time-scaled ML phylogeny constructed using LSD2 was supplied as input to the "treeWAS" function's "tree" argument (see section "Variant calling and maximum likelihood phylogeny construction within Dataset 1 [U.S. bovine and human data]" above); (iv) the number of simulated loci for estimating the null distribution was set to five million (i.e., n.snps.sim = 5000000); (v) ancestral state reconstruction performed using ML methods (i.e., snps.reconstruction = "ML", snps.sim.reconstruction = "ML", and phen.reconstruction = "ML"); (vi) a *P*-value significance threshold of 0.1, after controlling the FDR (p.value.correct = "fdr"). The analysis was re-run, using parsimony approaches in place of ML approaches for ancestral

state reconstruction. Regardless of approach, no orthologous gene clusters were found to be significantly associated with isolation source via any of the treeWAS association tests (FDR-corrected *P*-value > 0.1).

Acquisition of global DT104 complex genomic data and metadata. To compare the 230 U.S. human- and bovine-associated DT104 complex genomes in Dataset 1 (U.S. bovine and human data) to a larger set of DT104 complex genomes from numerous sources worldwide, genomic data associated with the following studies were downloaded via Enterobase: (i) Illumina reads associated with 243 bovine- and human-associated DT104 isolates from a study of between-host transmission within Scotland [75] (referred to hereafter as "Dataset 2 [Scottish bovine and human data]"); genomes were pre-processed and assembled as described above (see section "Acquisition of U.S. human- and bovine-associated DT104 complex genomic data and metadata" above); (ii) assembled genomes associated with 290 DT104 isolates from a variety of sources and countries from a study describing the global spread of DT104 [13] (referred to hereafter as "Dataset 3 [multi-source data]"; eleven of the 290 genomes were isolated from cattle and humans in the U.S. and thus had also been included in Dataset 1 [U.S. bovine and human data], Figure 1B, Supplementary Figures S2 and S3, and Supplementary Table S1).

The following datasets were aggregated to create a final set of 752 DT104 complex genomes derived from multiple different countries and isolation sources, which was used in subsequent steps (referred to hereafter as "Dataset 4 [combined global dataset]"; Figure 1B, Supplementary Figures S2 and S3, and Supplementary Tables S1 and S2): (i) Dataset 1 (U.S. bovine and human data) (n = 230 DT104 complex genomes), (ii) Dataset 2 (Scottish bovine and human data) (n = 243 DT104 genomes), and (iii) Dataset 3 (multi-source data) (n = 290 DT104 genomes, including 11 genomes that were part of Dataset 1 [U.S. bovine and human data]).

QUAST version 4.5 was used to assess the quality of all 752 genomes in Dataset 4 (combined global dataset) (Supplementary Tables S1 and S2). Prophage, AMR genes, plasmid replicons, and virulence factors were detected in all 752 Dataset 4 (combined global dataset) genomes as described above (see section "*In silico* detection of prophage, antimicrobial resistance genes, plasmid replicons, and virulence factors" above; Supplementary Tables S3-S5).

Variant calling and maximum likelihood phylogeny construction within Dataset 4 (combined global dataset). To identify core SNPs present in all 752 DT104 complex genomes within Dataset 4 (combined global dataset), Parsnp and HarvestTools version 1.2 [76] were used, as Parsnp easily scales to large data sets (Supplementary Tables S1 and S2) [76]. Assembled genomes were used as input for Parsnp, along with the closed DT104 chromosome as a reference (NCBI Nucleotide accession NC_022569.1) and Parsnp's implementation of PhiPack [77] to filter recombination.

Core SNPs detected among all 752 assembled genomes within Dataset 4 (combined global dataset) were supplied as input to IQ-TREE version 1.5.4, which was used to construct a ML phylogeny as described above (the corresponding ascertainment bias correction here was "-fconst 1181208,1285673,1280769,1179580"; see section "Variant calling and maximum likelihood phylogeny construction within Dataset 1 [U.S. bovine and human data]" above). The resulting ML phylogeny was rooted and time-scaled using LSD2 as described above (see section "Variant calling and maximum likelihood phylogeny construction within Dataset 1 [U.S. bovine and human data]" above; Supplementary Data). A range of 1900-2017 was supplied for four genomes, which were part of Dataset 3 (multi-source data), but did not have a reported year of isolation. The resulting LSD2 time-scaled ML phylogeny was annotated using the Interactive Tree of Life (iTOL) version 6 webserver (https://itol.embl.de/, accessed March 7, 2022; Figure 5,

Supplementary Figure S14, Supplementary Data) [78]. The LSD2 time-scaled ML phylogeny for Dataset 4 (combined global dataset) was further used for *artAB* presence/absence ancestral state reconstruction as described above (see section "*artAB* ancestral state reconstruction for Dataset 1 [U.S. bovine and human data]" above; Supplementary Figure S15).

Pan-genome characterization of Dataset 4 (combined global dataset). Pan-genome analyses were carried out for Dataset 4 (combined global dataset) as described above (see section "Pan-genome characterization of Dataset 1 [U.S. bovine and human data]" above; Supplementary Tables S1 and S2). Briefly, Prokka was used to annotate all 752 genomes within Dataset 4 (combined global dataset). GFF files produced by Prokka were supplied as input to Panaroo, which was used to identify core- and pan-genome orthologous gene clusters among all 752 Dataset 4 (combined global dataset) genomes. The pan-genome size for Dataset 4 (combined global dataset) was estimated using Panaroo's "panaroo-img" and "panaroo-fmg" commands, using the LSD2 time-scaled ML phylogeny for Dataset 4 (combined global dataset) as input (Supplementary Figure S13). Reference pan-genome CDS identified by Panaroo underwent functional annotation using eggNOG-mapper.

Strain selection for phenotypic stress assays. Phenotypic stress assays (discussed in detail in the sections below) were used to compare (i) bovine- and human-associated, Gifsy-1/*artAB*/*gogB*-positive U.S. DT104 complex strains to (ii) bovine- and human-associated, Gifsy-1/*artAB*/*gogB*-negative U.S. DT104 complex strains. Thus, the genomes of 13 bovine- and human-associated DT104 complex strains from New York State [1], which were available to us in the Cornell University Food Safety Laboratory (CUFSL) culture collection [79], were characterized further (Supplementary Figures S2 and S3 and Supplementary Table S2).

To identify a set of closely related, Gifsy-1/*artAB*/*gogB*-positive and -negative genomes for experimental characterization, Parsnp and HarvestTools version 1.2 [76] were used to identify core SNPs among all 13 genomes of New York State DT104 complex strains available in the CUFSL culture collection (Supplementary Figures S2 and S3 and Supplementary Table S2). Assembled genomes were supplied as input to Parsnp, along with the closed DT104 chromosome as a reference (NCBI Nucleotide accession NC_022569.1) and Parsnp's implementation of PhiPack [77] to remove recombination. IQ-TREE version 1.5.4 was used to construct a ML phylogeny, using (i) the resulting core SNPs as input, (ii) an ascertainment bias correction, based on the GC content of the DT104 reference chromosome ("-fconst 1182070,1287912,1283169,1180480"), (iii) the optimal nucleotide substitution model ("-m MFP"), selected using ModelFinder (i.e., the TIM+I model), and (iv) 1,000 replicates of the ultrafast bootstrap approximation ("-bb 1000").

Further steps were taken to ensure that the selected DT104 complex strains were as similar as possible in terms of their pan-genome composition. Briefly, Prokka version 1.13 was used to annotate each genome (using the "Bacteria" database, plus default settings). The resulting GFF files were supplied to Roary version 3.13.0 [80], which was used to identify orthologous gene clusters among the 13 DT104 complex genomes available for experimental characterization (using default thresholds, e.g., 95% protein BLAST [blastp] identity; Supplementary Table S2).

The New York State DT104 complex genomes differed little in terms of their core and pan-genome compositions (Supplementary Figure S16 and Supplementary Table S2). A total of 336 core SNPs were identified among the 13 DT104 complex genomes; pairwise core SNP distances between all 13 genomes ranged from 12-113 core SNPs (median and mean of 85 and

80.8 core SNPs, respectively, calculated using the "dist.gene" function in the ape R package). Based on gene presence/absence of pan-genome elements identified via Roary, the Jaccard distance between all 13 genomes ranged from 0.0036-0.0820 (median and mean of 0.0359 and 0.0380, respectively; calculated in R using the "vegdist" function in vegan version 2.5-7) [81].

Considering both (i) core- and pan-genome similarities between all 13 available New York State DT104 complex genomes, as well as (ii) Gifsy-1/*artAB*/*gogB* presence and absence, we selected six closely related, New York State DT104 complex strains to undergo phenotypic characterization (i.e., three Gifsy-1/*artAB*/*gogB*-positive strains, and three Gifsy-1/*artAB*/*gogB*-negative strains; Supplementary Table S11). Briefly, all available Gifsy-1/*artAB*/*gogB*-negative strains in the CUFSL culture collection were selected to undergo phenotypic testing ($n = 3$, two human isolates and one bovine isolate; Supplementary Table S11); all three strains were members of the U.S. *artAB*-negative major clade (discussed in detail in the "Results" section below). Considering both core- and pan-genome distances relative to all three available Gifsy-1/*artAB*/*gogB*-negative strains, three Gifsy-1/*artAB*/*gogB*-positive DT104 complex strains were additionally selected to undergo phenotypic testing (one from human and two from bovine sources; Figure 5, Supplementary Figure S16, and Supplementary Table S11). The three selected Gifsy-1/*artAB*/*gogB*-positive DT104 complex strains differed from the three available Gifsy-1/*artAB*/*gogB*-negative strains by (i) 64-83 (HUM_TYPH_NY_04_S5_0370), 74-93 (BOV_TYPH_NY_99_A4_0023), and 65-84 (BOV_TYPH_NY_99_S3_0910) core SNPs and (ii) Jaccard distances (based on pan-genome element presence/absence) of 0.0148-0.0610 (HUM_TYPH_NY_04_S5_0370), 0.0174-0.0622 (BOV_TYPH_NY_99_A4_0023) and 0.0163-0.0620 (BOV_TYPH_NY_99_S3_0910; Figure 5, Supplementary Figure S16, and Supplementary Table S11).

Phenotypic assays. All strain stocks (Supplementary Table S11) were maintained in CRYOBANK® tubes (Mast Ltd., Reinfeld, Germany) at -80°C. Strains were streaked out from stocks on tryptic soy agar (TSA; Merck KGaA, Darmstadt, Germany) and incubated overnight at 37°C. Single colonies from those plates were inoculated in 5 mL of tryptic soy broth (TSB; Merck KGaA, Darmstadt, Germany) and incubated for 16 - 18 h at 37°C with shaking at 200 rpm. The resulting overnight cultures were diluted 1/100 into 5 mL of fresh, pre-warmed TSB, followed by incubation at 37°C with shaking at 200 rpm to allow cultures to reach mid log phase (defined as OD_{600} of 0.4; 1-2 x10^8 CFU/mL). These cultures were used as input into three different phenotypic assays (exposure to ruminal fluid, acid stress, and bile stress; discussed in detail below). Bacterial enumeration before and after stress exposure was performed by direct colony counts of tilt plates according to Kühbacher et al. [82].

To evaluate exposure to ruminal fluid (RF), approximately 2 L of RF was acquired from a Jersey cow with a ruminal fistula on each experimental day prior to the experiments (same collection time was used for each experiment). The RF was immediately filtered through a cellulose filter (Labsolute® Type 80, Th. Geyer GmbH& Co. KG., Renningen, Germany) to remove any large debris, and the pH was measured, ranging from 7.20 to 7.62. Mid-log phase cultures were prepared and inoculated into the RF at two different concentrations. One hundred µl of culture suspensions were inoculated into 5mL of the RF at final concentrations of 10^8 (high) and 10^5 (low) CFU/mL and incubated for 1 h at 37°C without shaking with enumeration by direct colony counting on XLT-4 agar (Oxoid Ltd., Basingstoke, UK) prior and after RF exposure (Supplementary Table S12). The absence of *Salmonella* in the RF at the start of the experiments was confirmed by plating on XLT-4 agar.

Acid stress resistance of the different strains at pH 3.5 with and without prior adaption was tested using an adopted protocol from Horlbog et al. [83]. To carry out the acid stress assay, the pH of the TSB was adjusted with hydrochloric acid solution (1M and 6 M HCL; Merck KGaA, Darmstadt, Germany) immediately prior to the experiment. 1 mL aliquots of mid log phase cultures were transferred to reaction tubes and centrifuged at 14,000 x g for 10 min. For the non-adapted acid stress experiments, the pellets were resuspended in 1mL TSB pH 3.5 and incubated for 1 h at 37°C without shaking. For acid adaption, 1 mL of the same cultures were pelleted, resuspended in 1 mL TSB adjusted to pH 5.5 and incubated for 1h at 37°C (without shaking). Afterwards, the cultures were centrifuged again, resuspended in 1mL TSB pH 3.5, and incubated for 1 h at 37°C without shaking. Bacteria enumeration was performed before and after the one-hour incubation at pH 3.5 (Supplementary Table S13).

Susceptibility to bile salts (cholic acid and deoxycholic acid in a mixture of 1:1, Bile Salts No.3, Thermo Fisher Scientific Inc., Waltham, USA) was tested in two different concentrations: 14.5 mmol/L corresponding to 0.6% [84] and 26.0 mmol/L corresponding to 1.1% [85] were chosen to represent reasonable physiological states in the duodenum. Bile salts were added, and the pH of the TSB was adjusted to 5.5 (TSB-bile) immediately prior to the experiment. Mid log phase cultures were centrifuged, resuspended in TSB-bile, incubated for 1h 37°C without shaking, and enumerated by direct colony counting prior and after bile exposure (Supplementary Table S14).

For each stress assay, base-ten logarithmic fold change (FC) values were calculated as follows: FC = log CFU/g at the start of the experiments – log CFU/g after the stress assay. Analysis of Variance (ANOVA) for the interpretation of the phenotypic assays were conducted

using the "aov" function in R's "stats" package, with the FC values for the respective assay treated as a response. Figures were designed using the ggplot2 package.

Data availability. Strain metadata, genome quality metrics, and Enterobase accession numbers for all publicly available genomes queried in this study are available in Supplementary Table S1. Strain metadata, genome quality metrics, Food Microbe Tracker IDs [79], and NCBI BioSample accession numbers [86] for the 13 New York State DT104 complex strains queried in this study (including those queried via phenotypic assays) are available in Supplementary Table S2. LSD2 results (for Dataset 1 [U.S. bovine and human data] and Dataset 4 [combined global dataset]) and BEAST2 results (for subsets of Dataset 1 [U.S. bovine and human data]) are available as Supplementary Data.

SUPPLEMENTARY REFERENCES

1. **Carroll LM, Huisman JS, Wiedmann M**. Twentieth-century emergence of antimicrobial resistant human- and bovine-associated *Salmonella enterica* serotype Typhimurium lineages in New York State. *Sci Rep* 2020;10(1):14428.
2. **Zhou Z, Alikhan N-F, Mohamed K, Fan Y, Achtman M**. The EnteroBase user's guide, with case studies on *Salmonella* transmissions, *Yersinia pestis* phylogeny, and *Escherichia* core genomic diversity. *Genome Res* 2020;30(1):138-152.
3. **Alikhan NF, Zhou Z, Sergeant MJ, Achtman M**. A genomic overview of the population structure of *Salmonella*. *PLoS Genet* 2018;14(4):e1007261.
4. **Leinonen R, Sugawara H, Shumway M, International Nucleotide Sequence Database C**. The sequence read archive. *Nucleic Acids Res* 2011;39(Database issue):D19-21.
5. **Kodama Y, Shumway M, Leinonen R, International Nucleotide Sequence Database C**. The Sequence Read Archive: explosive growth of sequencing data. *Nucleic Acids Res* 2012;40(Database issue):D54-56.
6. **Yoshida CE, Kruczkiewicz P, Laing CR, Lingohr EJ, Gannon VP et al.** The *Salmonella In Silico* Typing Resource (SISTR): An Open Web-Accessible Tool for Rapidly Typing and Subtyping Draft *Salmonella* Genome Assemblies. *PLoS One* 2016;11(1):e0147101.
7. **Tonkin-Hill G, Lees JA, Bentley SD, Frost SDW, Corander J**. RhierBAPS: An R implementation of the population clustering algorithm hierBAPS. *Wellcome Open Res* 2018;3:93.
8. **Bolger AM, Lohse M, Usadel B**. Trimmomatic: a flexible trimmer for Illumina sequence data. *Bioinformatics* 2014;30(15):2114-2120.
9. **Bankevich A, Nurk S, Antipov D, Gurevich AA, Dvorkin M et al.** SPAdes: a new genome assembly algorithm and its applications to single-cell sequencing. *J Comput Biol* 2012;19(5):455-477.
10. **Andrews S**. FastQC: a quality control tool for high throughput sequence data. https://www.bioinformatics.babraham.ac.uk/projects/fastqc/.
11. **Gurevich A, Saveliev V, Vyahhi N, Tesler G**. QUAST: quality assessment tool for genome assemblies. *Bioinformatics* 2013;29(8):1072-1075.
12. **Ewels P, Magnusson M, Lundin S, Kaller M**. MultiQC: summarize analysis results for multiple tools and samples in a single report. *Bioinformatics* 2016;32(19):3047-3048.
13. **Leekitcharoenphon P, Hendriksen RS, Le Hello S, Weill FX, Baggesen DL et al.** Global Genomic Epidemiology of *Salmonella enterica* Serovar Typhimurium DT104. *Appl Environ Microbiol* 2016;82(8):2516-2526.
14. **Arndt D, Marcu A, Liang Y, Wishart DS**. PHAST, PHASTER and PHASTEST: Tools for finding prophage in bacterial genomes. *Brief Bioinform* 2019;20(4):1560-1567.
15. **Arndt D, Grant JR, Marcu A, Sajed T, Pon A et al.** PHASTER: a better, faster version of the PHAST phage search tool. *Nucleic Acids Res* 2016;44(W1):W16-21.
16. **Seemann T**. Prokka: rapid prokaryotic genome annotation. *Bioinformatics* 2014;30(14):2068-2069.
17. **Gilchrist CLM, Chooi YH**. Clinker & clustermap.js: Automatic generation of gene cluster comparison figures. *Bioinformatics* 2021.
18. **Seemann T**. ABRicate: Mass screening of contigs for antimicrobial resistance or virulence genes. 2018. https://github.com/tseemann/abricate.

19. **Feldgarden M, Brover V, Haft DH, Prasad AB, Slotta DJ et al.** Validating the AMRFinder Tool and Resistance Gene Database by Using Antimicrobial Resistance Genotype-Phenotype Correlations in a Collection of Isolates. *Antimicrob Agents Chemother* 2019;63(11).

20. **Carattoli A, Zankari E, Garcia-Fernandez A, Voldby Larsen M, Lund O et al.** *In silico* detection and typing of plasmids using PlasmidFinder and plasmid multilocus sequence typing. *Antimicrob Agents Chemother* 2014;58(7):3895-3903.

21. **Chen L, Yang J, Yu J, Yao Z, Sun L et al.** VFDB: a reference database for bacterial virulence factors. *Nucleic Acids Res* 2005;33(Database issue):D325-328.

22. **Owen SV, Wenner N, Canals R, Makumi A, Hammarlof DL et al.** Characterization of the Prophage Repertoire of African *Salmonella* Typhimurium ST313 Reveals High Levels of Spontaneous Induction of Novel Phage BTP1. *Front Microbiol* 2017;8:235.

23. **Camacho C, Coulouris G, Avagyan V, Ma N, Papadopoulos J et al.** BLAST+: architecture and applications. *BMC Bioinformatics* 2009;10:421.

24. **Hunt M, Mather AE, Sanchez-Buso L, Page AJ, Parkhill J et al.** ARIBA: rapid antimicrobial resistance genotyping directly from sequencing reads. *Microb Genom* 2017;3(10):e000131.

25. **Seemann T**. Snippy: Rapid haploid variant calling and core genome alignment. https://github.com/tseemann/snippy.

26. **Li H**. Aligning sequence reads, clone sequences and assembly contigs with BWA-MEM. *arXiv* 2013:1303.3997.

27. **Li H, Durbin R**. Fast and accurate short read alignment with Burrows-Wheeler transform. *Bioinformatics* 2009;25(14):1754-1760.

28. **Li H**. Minimap2: pairwise alignment for nucleotide sequences. *Bioinformatics* 2018;34(18):3094-3100.

29. **Li H, Handsaker B, Wysoker A, Fennell T, Ruan J et al.** The Sequence Alignment/Map format and SAMtools. *Bioinformatics* 2009;25(16):2078-2079.

30. **Quinlan AR, Hall IM**. BEDTools: a flexible suite of utilities for comparing genomic features. *Bioinformatics* 2010;26(6):841-842.

31. **Quinlan AR**. BEDTools: The Swiss-Army Tool for Genome Feature Analysis. *Curr Protoc Bioinformatics* 2014;47:11 12 11-34.

32. **Li H**. A statistical framework for SNP calling, mutation discovery, association mapping and population genetical parameter estimation from sequencing data. *Bioinformatics* 2011;27(21):2987-2993.

33. **Garrison E, Marth G**. Haplotype-based variant detection from short-read sequencing. *arXiv* 2012:1207.3907.

34. **Cleary JG, Braithwaite R, Gaastra K, Hilbush BS, Inglis S et al.** Comparing Variant Call Files for Performance Benchmarking of Next-Generation Sequencing Variant Calling Pipelines. *bioRxiv* 2015:023754.

35. **Tan A, Abecasis GR, Kang HM**. Unified representation of genetic variants. *Bioinformatics* 2015;31(13):2202-2204.

36. **Cingolani P, Platts A, Wang le L, Coon M, Nguyen T et al.** A program for annotating and predicting the effects of single nucleotide polymorphisms, SnpEff: SNPs in the genome of *Drosophila melanogaster* strain w1118; iso-2; iso-3. *Fly (Austin)* 2012;6(2):80-92.

37. **Seemann T**. samclip: Filter SAM file for soft and hard clipped alignments. https://github.com/tseemann/samclip.

38. **Li H**. Seqtk: a fast and lightweight tool for processing sequences in the FASTA or FASTQ format. https://github.com/lh3/seqtk.
39. **Page AJ, Taylor B, Delaney AJ, Soares J, Seemann T et al.** SNP-sites: rapid efficient extraction of SNPs from multi-FASTA alignments. *Microb Genom* 2016;2(4):e000056.
40. **Croucher NJ, Page AJ, Connor TR, Delaney AJ, Keane JA et al.** Rapid phylogenetic analysis of large samples of recombinant bacterial whole genome sequences using Gubbins. *Nucleic Acids Res* 2015;43(3):e15.
41. **Nguyen LT, Schmidt HA, von Haeseler A, Minh BQ**. IQ-TREE: a fast and effective stochastic algorithm for estimating maximum-likelihood phylogenies. *Mol Biol Evol* 2015;32(1):268-274.
42. **Kalyaanamoorthy S, Minh BQ, Wong TKF, von Haeseler A, Jermiin LS**. ModelFinder: fast model selection for accurate phylogenetic estimates. *Nat Methods* 2017;14(6):587-589.
43. **Kimura M**. Estimation of evolutionary distances between homologous nucleotide sequences. *Proc Natl Acad Sci U S A* 1981;78(1):454-458.
44. **Minh BQ, Nguyen MA, von Haeseler A**. Ultrafast approximation for phylogenetic bootstrap. *Mol Biol Evol* 2013;30(5):1188-1195.
45. **Hoang DT, Chernomor O, von Haeseler A, Minh BQ, Vinh LS**. UFBoot2: Improving the Ultrafast Bootstrap Approximation. *Mol Biol Evol* 2018;35(2):518-522.
46. **Rambaut A, Lam TT, Max Carvalho L, Pybus OG**. Exploring the temporal structure of heterochronous sequences using TempEst (formerly Path-O-Gen). *Virus Evol* 2016;2(1):vew007.
47. **To T-H, Jung M, Lycett S, Gascuel O**. Fast Dating Using Least-Squares Criteria and Algorithms. *Systematic Biology* 2015;65(1):82-97.
48. **Rambaut A**. FigTree: a graphical viewer of phylogenetic trees. http://tree.bio.ed.ac.uk/software/figtree/.
49. **Boskova V, Stadler T**. PIQMEE: Bayesian phylodynamic method for analysis of large datasets with duplicate sequences. *Molecular Biology and Evolution* 2020.
50. **Bouckaert R, Heled J, Kuhnert D, Vaughan T, Wu CH et al.** BEAST 2: a software platform for Bayesian evolutionary analysis. *PLoS Comput Biol* 2014;10(4):e1003537.
51. **Bouckaert R, Vaughan TG, Barido-Sottani J, Duchene S, Fourment M et al.** BEAST 2.5: An advanced software platform for Bayesian evolutionary analysis. *PLoS Comput Biol* 2019;15(4):e1006650.
52. **Bouckaert R**. 2014. Correcting for constant sites in BEAST2. https://groups.google.com/forum/#!topic/beast-users/QfBHMOqImFE [accessed March 1, 2023].
53. **Bouckaert RR, Drummond AJ**. bModelTest: Bayesian phylogenetic site model averaging and model comparison. *BMC Evol Biol* 2017;17(1):42.
54. **Drummond AJ, Ho SY, Phillips MJ, Rambaut A**. Relaxed phylogenetics and dating with confidence. *PLoS Biol* 2006;4(5):e88.
55. **Drummond AJ, Rambaut A, Shapiro B, Pybus OG**. Bayesian coalescent inference of past population dynamics from molecular sequences. *Mol Biol Evol* 2005;22(5):1185-1192.
56. **R Core Team**. R: A Language and Environment for Statistical Computing. R Foundation for Statistical Computing, Vienna, Austria; 2023. https://www.R-project.org/.
57. **Wickham H**. *ggplot2: Elegant Graphics for Data Analysis*: Springer-Verlag New York; 2016.

58. **Yu G, Smith DK, Zhu H, Guan Y, Lam TT-Y**. ggtree: an r package for visualization and annotation of phylogenetic trees with their covariates and other associated data. *Methods in Ecology and Evolution* 2017;8(1):28-36.

59. **Yu G, Lam TT, Zhu H, Guan Y**. Two Methods for Mapping and Visualizing Associated Data on Phylogeny Using Ggtree. *Mol Biol Evol* 2018;35(12):3041-3043.

60. **R Hackathon**. phylobase: Base Package for Phylogenetic Structures and Comparative Data. https://CRAN.R-project.org/package=phylobase.

61. **Yu G**. treeio: Base Classes and Functions for Phylogenetic Tree Input and Output. https://guangchuangyu.github.io/software/treeio.

62. **Revell LJ**. phytools: an R package for phylogenetic comparative biology (and other things). *Methods in Ecology and Evolution* 2012;3(2):217-223.

63. **Paradis E, Claude J, Strimmer K**. APE: Analyses of Phylogenetics and Evolution in R language. *Bioinformatics* 2004;20(2):289-290.

64. **Paradis E, Schliep K**. ape 5.0: an environment for modern phylogenetics and evolutionary analyses in R. *Bioinformatics* 2019;35(3):526-528.

65. **Tonkin-Hill G, MacAlasdair N, Ruis C, Weimann A, Horesh G et al.** Producing polished prokaryotic pangenomes with the Panaroo pipeline. *Genome Biol* 2020;21(1):180.

66. **Katoh K, Standley DM**. MAFFT multiple sequence alignment software version 7: improvements in performance and usability. *Mol Biol Evol* 2013;30(4):772-780.

67. **Katoh K, Misawa K, Kuma K, Miyata T**. MAFFT: a novel method for rapid multiple sequence alignment based on fast Fourier transform. *Nucleic Acids Res* 2002;30(14):3059-3066.

68. **Collins RE, Higgs PG**. Testing the infinitely many genes model for the evolution of the bacterial core genome and pangenome. *Mol Biol Evol* 2012;29(11):3413-3425.

69. **Baumdicker F, Hess WR, Pfaffelhuber P**. The infinitely many genes model for the distributed genome of bacteria. *Genome Biol Evol* 2012;4(4):443-456.

70. **Zamani-Dahaj SA, Okasha M, Kosakowski J, Higgs PG**. Estimating the Frequency of Horizontal Gene Transfer Using Phylogenetic Models of Gene Gain and Loss. *Mol Biol Evol* 2016;33(7):1843-1857.

71. **Huerta-Cepas J, Szklarczyk D, Heller D, Hernandez-Plaza A, Forslund SK et al.** eggNOG 5.0: a hierarchical, functionally and phylogenetically annotated orthology resource based on 5090 organisms and 2502 viruses. *Nucleic Acids Res* 2019;47(D1):D309-D314.

72. **Cantalapiedra CP, Hernandez-Plaza A, Letunic I, Bork P, Huerta-Cepas J**. eggNOG-mapper v2: Functional Annotation, Orthology Assignments, and Domain Prediction at the Metagenomic Scale. *Mol Biol Evol* 2021;38(12):5825-5829.

73. **Benjamini Y, Hochberg Y**. Controlling the False Discovery Rate: A Practical and Powerful Approach to Multiple Testing. *Journal of the Royal Statistical Society Series B (Methodological)* 1995;57(1):289-300.

74. **Collins C, Didelot X**. A phylogenetic method to perform genome-wide association studies in microbes that accounts for population structure and recombination. *PLoS Comput Biol* 2018;14(2):e1005958.

75. **Mather AE, Reid SW, Maskell DJ, Parkhill J, Fookes MC et al.** Distinguishable epidemics of multidrug-resistant Salmonella Typhimurium DT104 in different hosts. *Science* 2013;341(6153):1514-1517.

76. **Treangen TJ, Ondov BD, Koren S, Phillippy AM**. The Harvest suite for rapid core-genome alignment and visualization of thousands of intraspecific microbial genomes. *Genome Biol* 2014;15(11):524.

77. **Bruen TC, Philippe H, Bryant D**. A simple and robust statistical test for detecting the presence of recombination. *Genetics* 2006;172(4):2665-2681.

78. **Letunic I, Bork P**. Interactive Tree Of Life (iTOL) v5: an online tool for phylogenetic tree display and annotation. *Nucleic Acids Res* 2021;49(W1):W293-W296.

79. **Vangay P, Fugett EB, Sun Q, Wiedmann M**. Food microbe tracker: a web-based tool for storage and comparison of food-associated microbes. *J Food Prot* 2013;76(2):283-294.

80. **Page AJ, Cummins CA, Hunt M, Wong VK, Reuter S et al.** Roary: rapid large-scale prokaryote pan genome analysis. *Bioinformatics* 2015;31(22):3691-3693.

81. **Oksanen J, Blanchet FG, Friendly M, Kindt R, Legendre P et al.** vegan: Community Ecology Package. https://CRAN.R-project.org/package=vegan.

82. **Kuhbacher A, Cossart P, Pizarro-Cerda J**. Internalization assays for *Listeria monocytogenes*. *Methods Mol Biol* 2014;1157:167-178.

83. **Horlbog JA, Kent D, Stephan R, Guldimann C**. Surviving host - and food relevant stresses: phenotype of *L. monocytogenes* strains isolated from food and clinical sources. *Sci Rep* 2018;8(1):12931.

84. **Fausa O**. Duodenal bile acids after a test meal. *Scand J Gastroenterol* 1974;9(6):567-570.

85. **Guariglia-Oropeza V, Orsi RH, Guldimann C, Wiedmann M, Boor KJ**. The *Listeria monocytogenes* Bile Stimulon under Acidic Conditions Is Characterized by Strain-Specific Patterns and the Upregulation of Motility, Cell Wall Modification Functions, and the PrfA Regulon. *Front Microbiol* 2018;9:120.

86. **Barrett T, Clark K, Gevorgyan R, Gorelenkov V, Gribov E et al.** BioProject and BioSample databases at NCBI: facilitating capture and organization of metadata. *Nucleic Acids Res* 2012;40(Database issue):D57-63.
